# Characteristics of Speech Sound Development in Preterm‐Born Children: A Scoping Review

**DOI:** 10.1111/1460-6984.70347

**Published:** 2026-10-16

**Authors:** Sam Burr, Sam Harding, Sarah Rudd

**Affiliations:** ^1^ Bristol Speech and Language Therapy Research Unit North Bristol NHS Trust Bristol UK; ^2^ Cardiff School of Sport and Health Sciences Cardiff Metropolitan University Cardiff UK; ^3^ Research and Development North Bristol NHS Trust Bristol UK; ^4^ Library and Knowledge Service North Bristol NHS Trust Bristol UK

**Keywords:** preterm‐born children, scoping review, speech sound development

## Abstract

**Background:**

There is substantial evidence for the detrimental impact of preterm birth on language development compared to children born at term. However, little is known regarding speech sound development of preterm infants. Speech development precedes language development and requires investigation in its own right. A clear picture of the evidence for speech sound development in preterm‐born children is important to enable clinical services in early identification and intervention for these children.

**Aim:**

To understand the extent and type of evidence relating to typical and atypical speech sound development in children born before 37 weeks gestation (preterm).

**Methods:**

A search of 5 MEDLINE, CINAHL, PsycInfo, EMBASE, EMCARE was undertaken in April 2026. Grey literature searching in Campbell collaboration, COSMIN, PROSPERO, FigShare and Open Science Framework, Google (advanced search), and SpeechBite was undertaken. Articles including samples of children reporting speech sound development (typical or atypical) were included in the review. Studies focusing on outcomes for reading and literacy were excluded, as were animal studies and those signed languages. Population demographics, assessment and outcome measures, methods of analysis and findings were extracted from each study.

**Main contribution:**

28 studies from 14 different countries were included in the review. Twenty‐five of the 28 studies reported differences in speech outcomes of PT children and FT children between the ages of 3 months and 12 years.

**Conclusions:**

This review found differences in how PT and FT children develop sounds for speech from birth through into childhood. Further large‐scale, longitudinal research is needed to clarify what typical and atypical speech development look like in PT children.

**WHAT THIS PAPER ADDS:**

*What is already known on this subject*

- The findings of this scoping review provide evidence of the differences between speech sound development between preterm born children and children born at term in the first 12 years of life. This information could contribute to a more informed approach to care and early intervention.

*What this study adds to the existing knowledge?*

- Understanding the impact of preterm birth on early speech sound development can help speech and language therapists to build a picture of a child's individual risk profile for speech sound disorder later in childhood. This can lead to earlier intervention for the children who need it most.

**Abbreviations**
BPD: bronchopulmonary dysplasia
BW: birth weight
CP: cerebral palsy
EPT: extremely preterm‐born/birth
FT: full‐term born/birth
GA: gestational age
IVH: intraventricular haemorrhage
JBI: Joanna Briggs Institute
MPT‐LPT: moderate to late preterm‐born/birth
PCC: Percentage Consonants Correct score
PRISMA_ScR: Preferred Reporting Items for systematic Reviews and Meta Analysis Scoping Review
PT: preterm‐born/birth
SLP: Speech and Language Pathology/Pathologist
SSD: Speech Sound Disorder
VPT: very preterm‐born/birth
WHO: World Health Organisation

## 1 Background

The aim of this paper is to understand the extent and type of evidence in relation to typical and atypical speech sound development in preterm‐born (PT) children (before 37 weeks’ completed gestation).

### 1.1 Terminology and Prevalence

Speech sound disorder (SSD) is defined as a “persistent difficulty with speech sound production that interferes with speech intelligibility or prevents verbal communication” (Harding et al. 2024). Estimates indicate an SSD prevalence of 2.3%–24.6% among children aged 4–8 years (Eadie et al. 2015; Shriberg et al. 1999; Wren et al. 2016). A minority of children with SSD have identifiable causes, including cleft palate, cerebral palsy (CP), or hearing impairment. The majority of SSD cases lack a known etiology, and there is limited evidence to advocate intervention in this demographic. If untreated, SSD can have a significant adverse effect on a person's mental health, career, and education (BEITCHMAN et al. 2001; Eadie et al. 2015; McAllister et al. 2023; McCormack et al. 2011; Shriberg et al. 1999; Wren et al. 2023; Wren et al. 2021).

Globally, around one in 10 children (approximately 13.4 million) is born preterm every year (World Health Organisation 2023). PT birth is defined as live birth before 37 weeks’ completed gestation and includes the sub‐categories extremely preterm (EPT) (< 28 weeks), very preterm (VPT) (28–32 weeks) and moderate (MPT) to late preterm (LPT) (32–37 weeks) (World Health Organisation 2023).

### 1.2 Motivation for the Review

PT birth increases the risk of neurodevelopmental problems in children, including in speech, language, and communication (National Institute for Health and Care Excellence (NICE) 2017; World Health Organisation 2023). It is well established that compared to full‐term (FT) born children, PT delivery has a detrimental impact on language development (Barre et al. 2011; Carter and Msall 2017; Capobianco and Cerniglia 2017; Putnick et al. 2017; Sanchez et al. 2020; Vandormael et al. 2019; Wolke and Meyer 1999). PT birth interrupts critical brain development and, while there is considerable heterogeneity within the population, the impact on early neuroplasticity is not yet fully established (DeMaster et al. 2019; Sahinoglu et al. 2026; Thalhammer et al. 2025). Speech sound development precedes language development, with the former widely understood to influence the latter (Bishop and Adams 1990; Overby et al. 2012; van Noort‐van der Spek et al. 2022). However, little is known about how PT children develop their speech sounds and the specific nature of the influence of prematurity on speech development is unclear (Wren et al. 2016). Recent studies have revealed a number of important risk factors for SSD, including exclusive bottle feeding during the first 2 years of life and inadequate sucking at 4 weeks of age (Wren et al. 2016; Burr 2022). Compared to FT infants, PT babies are more likely to experience these factors, which may increase their risk of developing SSD later. PT children are doubly affected by SSD, because prematurity disproportionately affects children from non‐white ethnic groups and experience greater social, economic and health inequalities (Campbell et al. 2003; Eadie et al. 2015; Locke 2000; McLeod and Harrison 2009, Office for National Statistics (ONS) 2022). The early life experiences of PT babies in the initial days and weeks following delivery are very different from FT children in relation to contact, care and communication. After birth, many PT babies spend long periods of time in neonatal intensive care units rather than in the home environment, and experience barriers to natural interaction with family members due to medical care needs (e.g., infection control measures). The early speech sound development of these children may also be impacted by these events.

### 1.3 Implications for Clinical Practice

One of the most important clinical indicators of speech and language problems for children in the general population is poor speech sound development in the first few years of life, such as delayed babbling and first words (Lang et al. 2019; McGillion et al. 2017; Moore et al., 2017; Oller et al. 1998; Oller et al. 1999; UK House of Commons Health and Social Care Committee, 2019; UNICEF, 2024). There is a wealth of data about the development of speech sounds in the general population, but little is known about if and how this varies in PT infants. Given the potential impact that the different life factors of PT children may have on their speech development, we need to know what ‘typical’ speech development in PT children looks like and how and if it differs from speech sound development in children born at term. This information will help clinicians to determine which children are more at risk for SSD so their development can be closely monitored to inform further care. Robust evidence on the characteristics of speech sound development among PT children is needed to inform clinical care and service development to ensure the needs of this highly vulnerable and disadvantaged group of children are identified and met as early as possible.

### 1.4 Reviews to Date

Preliminary searches on MEDLINE, the Cochrane Database of Systematic Reviews and Joanna Briggs Institute (JBI) Evidence Synthesis, showed that there are currently no systematic or scoping reviews on the topic being addressed within the current review.

This review uses a scoping review methodology to explore and map the evidence that has been published on the development of children born preterm who have SSD. Scoping reviews are recommended where a topic is complex or of a heterogeneous nature. Furthermore, where key concepts and outcome measures may vary, a scoping review is useful in identifying research gaps and informing future evidence synthesis (Arksey and O'Malley 2005; Munn et al. 2018; Peters et al. 2015). They are not intended to assess the effectiveness of interventions or to provide guidelines for best practice. Other searches conducted at the outset of this review revealed significant variation in terms of study design, age at time of assessment, use of gestational age and/or post‐conceptual age, use of ‘corrected’ versus ‘chronological’ age and a variety of different speech outcome measures. A scoping review methodology was, therefore, considered appropriate.

### 1.5 Review Objective

The objective of this scoping review is to explore the literature on speech sound development in PT children. Preliminary searches indicate that there are few publications specifically on speech sound development in PT children and that participant samples often include, but do not separately examine or evaluate PT children. The outcome of this review will provide critical evidence and synthesize existing knowledge to inform future research in this area, which could underpin critical service developments to improve outcomes for PT children.

### 1.6 Review Question

- What are the characteristics of speech sound development in PT children?

## 2 Methods and Analysis

This review was undertaken following the Joanna Briggs Institute (JBI) guidelines and reported in line with the Preferred Reporting Items for systematic Reviews and Meta Analysis Scoping Reviews (PRISMA‐ScR) (Tricco et al. 2018). An a priori protocol was registered with the Open Science Framework in April 2026 (https://osf.io/g5hq3).

The Population, Concept, Context framework was used to define the scoping review question (Aromataris 2020). The search strategy was developed, piloted, and refined with the support of a library knowledge specialist. (Supplementary material 1).

## 3 Inclusion Criteria

### 3.1 Participants

Children and young people aged under 18 years with a history of PT birth (born before 37 weeks completed gestation) and/or low BW (< 2500 g) (United Nations Children's Fund and World Health Organization 2004). A criterion for low BW, as defined by the WHO (United Nations Children's Fund and World Health Organization 2004), was used to ensure the search results included reports which may not specifically focus on GA, but which were highly likely to include PT children.

### 3.2 Concept

To be included in the scoping review, studies had to have assessed children's speech sound development (as defined in Participants section). This could include information about or descriptions of typical or atypical speech sound development, which may be described as articulation disorder, childhood apraxia of speech (formerly known as developmental verbal dyspraxia) or phonological disorder/delay. A broad range of quantitative and qualitative speech outcomes were eligible for inclusion in the review, including early speech categories (e.g., early vocalisations), parent reported measures (e.g., intelligibility), clinical metrics (e.g., percentage consonants correct) and research metrics (e.g., canonical babbling ratio (CBR). Excluded from the review were children with a known cause for their SSD, such as those with identified genetic or chromosomal anomalies, and congenital or acquired neurological conditions (e.g., cerebral palsy). Intraventricular hemorrhage (IVH) and bronchopulmonary dysplasia (BPD) are highly prevalent among PT children, and so studies which involved participants with these diagnoses were included to ensure maximal inclusion of children at the earliest extremes of prematurity (Burr 2022; Shukla et al. 2026). Information about speech input processing was not included in the review.

The child's age at assessment was an important consideration and included the child's corrected (adjusted) or chronological age of assessment. Corrected, or adjusted, age is calculated by subtracting the number of weeks a child was born from their chronological age. The use of chronological or corrected age has implications for outcome assessments, with more marked differences reported in early childhood compared with later childhood (D'Agostino 2010; Gould et al. 2021; Wilson 2004). Published evidence advocates for the use of corrected age in research due to the persistent bias resulting from preterm birth (D'Agostino 2010; Gould et al. 2021; Wilson 2004).

### 3.3 Context

This review included open context for reviews in that it considered studies taking place in any setting (e.g., home, clinic and nursery) and any geographical location.

#### 3.3.1 Types of Sources

The complete search included Ovid Medline, Ovid Embase, Cumulative Index to Nursing and Allied Health Literature (CINAHL), PsycInfo and Cochrane. These databases were selected because they cover a broad range of journals pertaining to medicine, psychology (including child development) and the allied health professions.

In addition to these standard journal databases, other platforms were integrated including Campbell collaboration, COnsensus‐based Standards for the selection of health Measurement INstruments, Figshare, JBI, Open Grey, Open Science Framework, PROSPERO, Google and Speechbite. Due to resource limitations, included studies were in English. Where a potentially relevant article could not be retrieved, direct contact with the study authors was made.

This scoping review considered both experimental and quasi‐experimental study designs including randomized controlled trials, non‐randomized controlled trials, before and after studies and interrupted time‐series studies. Analytical observational studies including prospective and retrospective cohort studies, case‐control studies and analytical cross‐sectional studies will be considered for inclusion. Descriptive observational study designs including case series, individual case reports and descriptive cross‐sectional studies were also considered for inclusion. Qualitative studies that focus on qualitative data including, but not limited to, designs such as phenomenology, grounded theory, ethnography, qualitative description, were considered. The bibliographies of systematic reviews that met the inclusion criteria were interrogated for relevant articles to be included.

#### 3.3.2 Search Strategy

The search strategies aimed to locate both published and unpublished studies. Published database searches were undertaken by a clinical librarian (WW) in consultation with subject experts (SB, SH), and grey literature by one of the authors (SH). The reference list of all included sources of evidence was screened for additional studies. Studies published since the year 1986 were included, in line with a large‐scale review in a related field (Harding et al. 2024). The searches were completed in April 2026 and are reported in line with the PRISMA‐ScR checklist.

#### 3.3.3 Study/Source of Evidence Selection

Following the search, all identified citations were collated and uploaded into EndNote X9 (Clarivate Analytics, PA, USA) and duplicates removed. Inclusion and exclusion criteria are presented in Table 1. The deduplicated references were then uploaded into the Rayyan reference management database (Ouzzani et al. 2016) allowing for blinded assessment of the papers between the authors. Following a pilot test, titles and abstracts were screened by two reviewers (SB, SH) for assessment against the inclusion criteria for the review. Potentially relevant sources were retrieved, and their citation details imported into Rayyan. The full texts of selected citations were assessed in detail against the inclusion criteria by two reviewers (SB, SH). Reasons for exclusion of sources of evidence at full text that did not meet the inclusion criteria were recorded and reported (Supplementary Material 1). Disagreements between the reviewers at each stage of the selection process were resolved through discussion. The numbers of articles at each stage of the search and the study inclusion process are reported in the PRISMA flow diagram (Tricco et al. 2018) [Figure 1].

**TABLE 1:** Inclusion and exclusion criteria.

Inclusion criteria	Exclusion criteria
‐ Children and young people aged under 18 years with a history of premature birth and/or birthweight below 2500 g ‐ English language paper ‐ Non‐animal study ‐ Report of speech sound development outcome measure (e.g. articulation, phonology, phoneme, phonetic, speech motor development, speech sound disorder of unknown origin)	‐ Non‐English language paper ‐ Focus on children with specific condition or syndrome known to impact speech development (e.g., cerebral palsy) ‐ Focus only on speech input processing ‐ Animal studies ‐ Intervention studies ‐ No focus on speech sound development outcomes ‐ Gestational age >37 weeks completed or GA not reported

**FIGURE 1:**
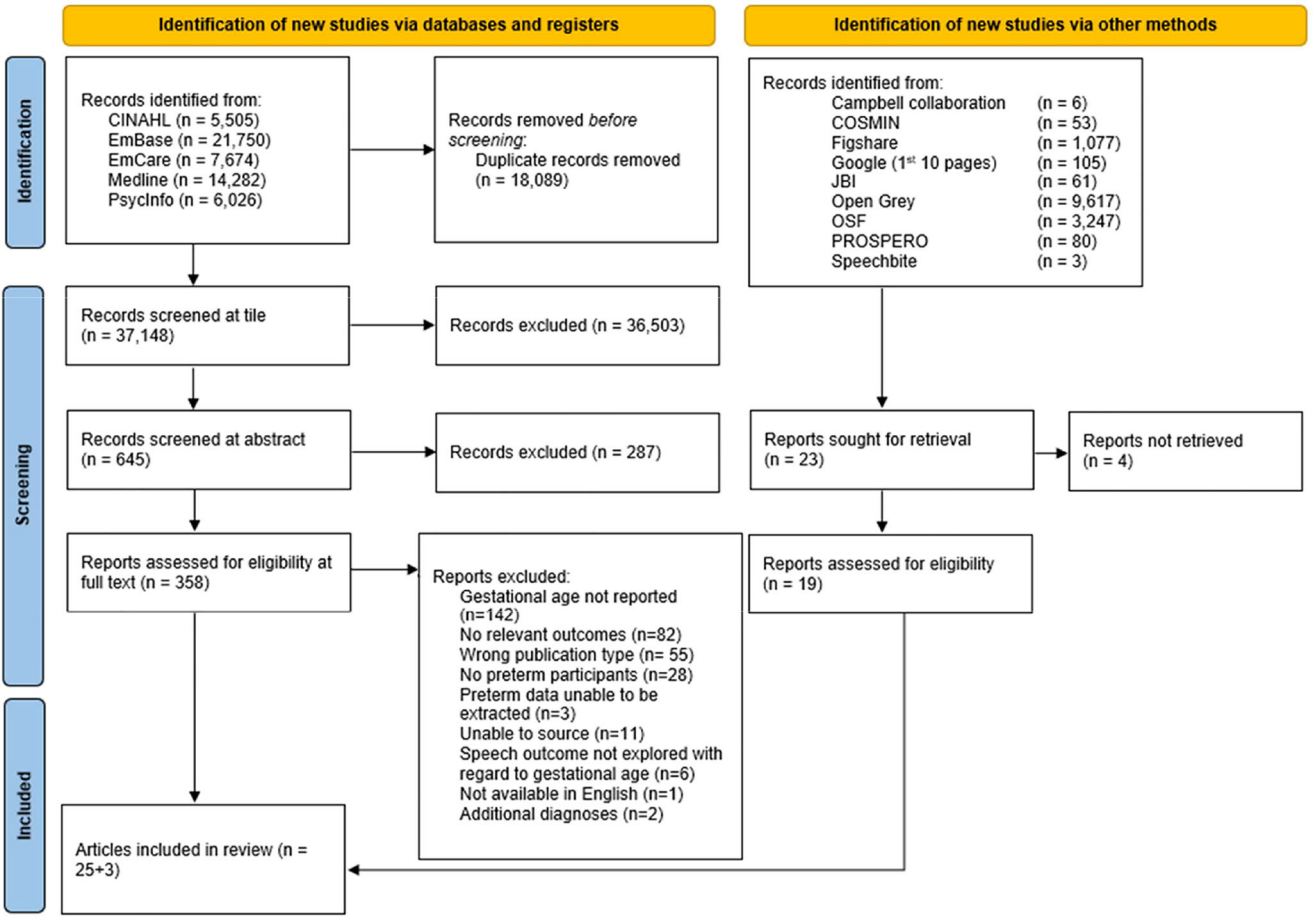
PRISMA ScR flow chart.

Relevant results from grey literature searches were captured in an Excel spreadsheet and agreement for inclusion in the review reached by SB and SH. Where other sources were identified (e.g., from the Google search) these were checked for academic citations and their bibliographies interrogated for relevant articles to be retained in the review.

### 3.4 Data Extraction

Data were extracted from included papers by two reviewers (SB, SH) using a data extraction tool developed by the authors and adapted from JBI guidance (Maggio et al. 2021). Table 2 presents a summary of the data extracted. Study design, sample size and population details including biological sex and gestational age, outcome measurement and measurement tools, context and key findings were extracted. Figure 1 presents the result of the search and selection of studies in the form of the PRISMA‐ScR.

**TABLE 2:** Data summary.

Author(s)	Study design	Aim(s) / purpose	Population and sample size	Inclusion criteria	Exclusion criteria
Brown et al. (1986) USA	Retrospective cross‐sectional study	Examine any differences in speech‐sound production between healthy PT and those who had suffered perinatal insult.	PT+IVH case group: *N* = 33, mean GA 30.64 (s.d. 1.69) wks, mean BW 1094 g (s.d. 161 g). IVH Grade I (*n* = 2), Grade I (*n* = 1), Grade III (*n* = 14), Grade IV (*n* = 4). PT‐IVH control group: ( *N* = 12), mean GA 29.7 (s.d. 1.49), mean BW 1206 g (s.d. 150 g). SES but no ethnicity.	PT birth; admission to the Neonatal Intensive Care Unit of St. Peter's Medical Center, New Brunswick, between May 1982‐March 1984.	Severe hearing loss.
D'Odorico et al. (2011) Italy	Prospective cohort study	Explore whether variables, such as BW and GA influence phonetic development.	PT case group: *N* = 24 (female *n* = 11; GA 26 to ≤ 32 weeks). FT control group: *N* = 15 (female *n* = 6). SES: maternal education ≥high school. Italian speaking. No further diversity characteristics reported.	BW < 2000 g and GA < 32 weeks	History of major medical injuries at birth.
Filgueira de Souza e Souza et al. (2019) Brazil	Cross‐sectional study	Check the use of phonological processes in PT children.	PT case group: *N* = 20 (female *n* = 14; GA 26–36 weeks; BW 530–2310 g) median 1647.5 g) FT control group: *N* = 20 (matched for age, gender and SES). Brazilian Portuguese. No further diversity characteristics reported.	Available data on BW and GA.	Diagnosis of brain lesion, genetic syndrome and/or psychiatric alterations; visual or auditory impairment, or any condition prohibiting participation
Goldfield (2000) USA	Cross‐sectional study	Explore how infants may use their own serially ordered vowel productions during vocal play to explore the relation between vocal tract shape and variation in acoustic information	PT case group: *N* = 16 (GA 28–36+6 weeks), sex data not reported. FT control group: *N* = 16 (female *n* = 8): BW 850–2450 g. No further diversity characteristics reported.	BW < 2500 g; mild/no respiratory distress; <Grade1 IVH	PT: IVH >Grade I, >moderate respiratory distress in first week of life. FT group: medical complications at birth, BW < 2500 g.
Holm and Crosbie (2010) Australia	Retrospective cohort study	Investigate the relationship between PT birth and literacy development	PT case group: *N* = 196, 84 F, mean GA 28.9wks (s.d. 2.1) mean BW 1287 g (s.d. 401.8); FT case group: *N* = 168, 80 F, mean GA 39.8wks (s.d. 1.1).	GA ≤ 32 weeks; attending mainstream schools in south‐east Queensland; monolingual English language development	Identified major physical or sensorineural impairment
Hong et al. (2026) Republic of Korea	Retrospective cohort study	Identify speech sound development trajectories in PT children; examine associated clinical and developmental characteristics, analyze factors related to speech sound abilities, and identify predictors of speech sound normalization.	PT case group: *N* = 80, 35 F, mean GA 29.6wks (s.d. 3.5), mean BW 1290.6 g (s.d. 507.6 g). BPD *n* = 30, Grad I‐II IVH ( *n* = 34). Previous intervention, *n* = 65. No ethnicity or SES.	GA < 37wks; BW < 2500 g; attended the Department of Rehabilitation Medicine at Asan Medical Center in Seoul, South Korea, between January 2014‐December 2023;	Hearing impairment; congenital anomalies affecting speech; oral feeding difficulties, structural/functional oral–motor abnormalities; CP, reported dysarthric speech.
Imgrund et al. (2023) USA	Retrospective cohort study	Describe the conversational speech production abilities of PT vs FT children using speech sample analysis	PT case group: *N* = 29 (GA 24–30 weeks, mean 31weeks, *SD* = 18 days; BW 610–2,340 g, mean 1507 g, *SD* = 461 g). Age 4;0–4;11 at assessment. FT control group: *N* = 22 (female *n* = 10, no GA/BW data reported. Mainly English‐speaking homes, Standard American English. Maternal education >college. Race and ethnicity reported.	PT Group: recruited from another study. FT Group: recruited from community, typical speech development (PCC ≥85%). Normal hearing.	GA ≤ 37 weeks, a diagnosed neurodevelopmental, sensory, or behavioural disorder, and a history of language, speech, or hearing impairment.
Jennische and Sedin (1998) Sweden	Retrospective cohort study	Evaluate the spontaneous speech of children who had required NIC and healthy controls at age 6.5 y to determine whether the characteristics of spontaneous speech differed a) between different NIC groups and B) between NIC groups and the control group and c) to analyse the associations between the spontaneous speech and neonatal factors.	NIC case group: Group I, GA 23–31 wks (*n* = 81; 35 F); subgroup IA, GA 23–27 wks (*n* = 27; 9 F), subgroup IB, GA 28–31 wks (*n* = 54; 26 F); group II, GA 32–36 wks (*n* = 132; 48 F), and group III, GA 37+ wk (*n* = 71; 25 F). Control group: *N* = 40, 20 F. No ethnicity or SES reported.	VPT born 1980–1985; required NIC at the Department of Pediatrics of Uppsala University Children's Hospital. Age 6.5‐y‐old at the time of the study. Resident in Uppsala. Mechanical ventilation / CPAP at birth, Apgar ≤ 5 at 5 min.	Not stated.
Jennische and Sedin (1999a) Sweden	Retrospective cohort study	Determine the occurrence of babbling and the ages at which different early stages of language development were reached	NIC case group: (*N* = 284) Control group: (*N* = 40). No ethnicity or SES reported.	VPT born 1980–1985; required NIC at the Department of Pediatrics of Uppsala University Children's Hospital. Age 6.5‐y‐old at the time of the study. Resident in Uppsala. Mechanical ventilation / CPAP at birth, Apgar ≤ 5 at 5 min.	Not stated.
Jennische and Sedin (1999b) Sweden	Retrospective cohort study	Compare linguistic skills of children aged 6½ y who had required NIC with those of healthy control children of the same age with the purpose of identifying weaknesses in linguistic areas and to analyse the linguistic skills of the NIC children in relation to gestational age and neonatal factors.	NIC case group: Group I, GA 23–31 wks (*n* = 81; 35 F); subgroup IA, GA 23–27 wks (*n* = 27; 9 F), subgroup IB, GA 28–31 wks (*n* = 54; 26 F); group II, GA 32–36 wks (*n* = 132; 48 F), and group III, GA 37+ wk (*n* = 71; 25 F). Control group: *n* = 40, 20 F. No ethnicity or SES report	VPT born 1980–1985; required NIC at the Department of Pediatrics of Uppsala University Children's Hospital. Age 6.5‐y‐old at the time of the study. Resident in Uppsala. Mechanical ventilation / CPAP at birth, Apgar ≤ 5 at 5 min.	Not stated.
Jennische and Sedin (2001) Sweden	Retrospective cohort study	Determine whether there were differences in spontaneous speech between groups of NIC children and to compare the spontaneous speech in NIC children with that in a group of neonatally healthy children.	*N* = 230. PT case group: (*N* = 64): Group I, GA 23–31 weeks (subgroup IA, GA 23–27 week (*n* = 12; female *n* = 7); subgroup IB, GA 28–31 week (*n* = 52; female *n* = 21)); Group II, GA (32–36 weeks) (*n* = 81; female *n* = 30). FT control group: (*N* = 70): Group III, GA 37 weeks (female *n* = 34). Higher than average mean SES, Swedish speaking. No further diversity characteristics reported.	Surviving children born from January 1986‐April 1989, with history of NIC, ventilatory assistance, total parenteral nutrition, severe asphyxia with an Apgar score 4 at 5 min, VPT birth < 32 gestational weeks, and/or treatment of severe neonatal convulsions.	Not stated.
Largo et al. (1986) Switzerland	Retrospective cohort study	To describe language development during the first five years of life of PT children and to compare this with a group of FT children	PT case group: *n* = 114, GA range 27.1–36.8 wks, BW range 1050–3460 g; FT control group: *n* = 97, GA range 37.1–42.6 wks, BW range 2420–4330 g. SES, no ethnicity.	Born 01/10/1974–30/09/1978; Swiss; admitted to the NICU of the Kinderspital and Fraueuklinik Zurich	Not stated.
Largo et al. (1990) Switzerland	Retrospective cohort study	To describe the speech development of high risk PT children with age appropriate BW.	PT case group: (*n* = 97), 43 F; FT control group: (*n* = 93), 46 F. SES, no ethnicity.	Born 01/10/1974–30/09/1978; Swiss; admitted to the NICU of the Kinderspital and Fraueuklinik Zurich	Not stated
Lewis et al. (2002) USA	Prospective cohort study	To examine how BPD impacts speech and language development.	Matched groups (ethnicity, SES and birth period): VLBW+BPD: *n* = 89, GA 27wks (s.d. 2.1) BW 970 g (s.d. 254 g), 47% F; VLBW‐BPD: *n* = 71, GA 30wks (s.d. 2.2), BW 1251 g (s.d. 179 g), 59% F; FT control group: *n* = 93, GA 40wks 9s.d. 1.2), BW 3467 g (s.d. 556 g), 52% F. No group differences in race, gender, SES, maternal education, and marital status	Infants with VLBW + BPD admitted to NICUs in the Cleveland region. BPD group: BPD infants were PT, BW < 1500 g, Hx supplementary oxygen >28 days. Partial stratification sampling to include mixed SES. Term infants from local nurseries.	Neurologic problems other than grades I–II IVH. Major congenital malformations; drug exposure; maternal major psychiatric or physical illness, human immunodeficiency virus; mental retardation, located >2 h away
Luoma et al. (1998) Finland	Prospective cohort study	To examine factors thought to be related to the inconclusive and complicated findings reported in earlier studies.	PT case group: *n* = 55; 28 F (*n* = 46; 22 F without major disability e.g., CP); Matched FT control group: *n* = 55 (*n* = 46 without disability). Chronological age. SES, no ethnicity.	Children born GA < 32 weeks in Kuopio University Hospital, Finalnd between 1 April 1984 and 31 March 1986.	Not stated.
Milankov and Mikov (2009) Serbia	Prospective cohort study	Demonstrate basic parameters, establish differences, determine characteristics of disorder of sound articulation in full term and preterm children.	PT case group: *N* = 30 (female *n* = 17; GA ≤ 32 weeks (mean 29 weeks, *SD* = 1.52), BW ≤ 1500 g). FT control group: *N* = 31 (female *n* = 10; GA ≥38 weeks, mean 39 weeks, *SD* = 1.08, BW ≥3000 g). Mean assessment age 4 years. SES not reported, Serbian speaking. No further diversity characteristics reported.	BW and GA data, Serbian speaking	Major neurologic issues or disabilities.
Oller et al. (1994) USA	Prospective cohort study	To evaluate quantitatively a variety of speech‐like vocalizations in PT and FT infants.	PT case group: *n* = 20; 13 F, mean BW 1820 g (range 1446–2070 g) mean GA 33 wks (range 3.5–11.5wks), low SES *n* = 10. FT control group: *n* = 33; 11 F,	Participants in a longitudinal study recruited from the local area.	Significant health problems (e.g., intraventricular haemorrhage, respiratory distress syndrome, severe hyperbilirubinemia, or any other important complications requiring perinatal medical intervention), five‐min Apgar scores >7.
Rvachew et al. (2005) Canada	Retrospective cohort study	Describe the vocalizations produced by VLBW PT infants aged 8–18 months.	Healthy PT group (no BPD): *N* = 9 (GA 28–36 weeks; BW 560–1050 g). BPD group: *N* = 13. Healthy FT control group: *N* = 10. All group means for SES in middle category. All English speaking. No further diversity characteristics reported.	Infants scheduled for 8‐month follow‐up assessments between 1998–1999 at the Perinatal Follow‐up Clinic, Alberta Children's Hospital.	BW ≥1250 g; major medical complications, excepting BPD (e.g. severe IVH); mental retardation^*^; known neurologic abnormalities, oral structure deficits, or sensory disorders.
Sanchez et al. (2020) Australia	Retrospective cohort study	Explore the differences between language, speech, and voice of PT and FT children at (corrected) age 3 years. Explore predictors of language, speech, and voice outcomes in PT children aged 3 years.	PT case group: *N* = 112 (female *n* = 57; mean GA 27.83 weeks; SD 1.44). FT control group: *N* = 105 (female *n* = 53; mean GA 40 weeks). Range of SES, English. No further diversity characteristics reported.	GA < 30 weeks. Recruited at birth from the Royal Women's Hospital and Frances Perry House Private Hospital, Melbourne, between January 2011‐March 2013	Congenital abnormalities known to affect neurodevelopment; non‐English speaking parents.
Shahramnia et al. (2023) Iran	Retrospective cross‐sectional study	Compare PT and FT speech sound production in single word and story retelling scores, speech intelligibility, oral motor performance and performance on single word and story retelling test. Describe the associations among the demographic variables and performance of participants in single word test and story retelling test Describe the associations among the oral motor scores of participants and their speech intelligibility.	PT case group: *N* = 31 (female *n* = 14; mean GA 31.8 weeks (SD 2.0); mean BW 1864 g (SD 356.7 g); mean assessment age 48.1 ± 6.7 months). FT control group: *N* = 29 (female *n* = 17) mean GA 38.4 ± 0.7 weeks, mean BW 2944 g ± 424.6 g, mean assessment age 49.1 ± 6.3 months. SES maternal education >diploma. No further diversity characteristics reported.	Persian first language, no major cerebral damage, any congenital malformations, chromosomal anomalies, severe neonatal, normal hearing, visual, and gross motor skills. PT group BW ≥1500 g. FT BW ≥2500 g. PT recruited from the NIC of Aliasghar Hospital of Iran University of Medical Sciences, Tehran, Iran, between 2014–2016. FT recruited from Persian‐speaking nursery schools across Tehran.	Persistent OME, CP, history of speech therapy sessions, below cutoff score of the Age and Stage Questionnaire in each domain.
Törölä et al. (2012) Finland	Prospective cohort study	To describe the early vocalization stages of PT infants.	PT case group: *N* = 18 (female (*n* = 7); BW ≤ 1000 g (mean 880 g, range 670–1020 g); mean GA 27 weeks (range 23–30 weeks)). FT control group: *N* = 11 (female (*n* = 7). Maternal education mostly ≥college/university degree. Finnish speaking. No further diversity characteristics reported.	PT births at Oulu University Hospital in Northern Finland from October 2003‐April. Healthy FT infants with normal hearing, whose parents volunteered and whose families were geographically attainable, born at Oulu University Hospital August‐November 2003	Congenital/chromosomal anomalies and hearing loss.
Törölä et al. (2012) Finland	Prospective cohort study	To systematically describe the PT preverbal development from canonical babbling up to the first word and to compare it with FT infants	PT case group: *N* = 18 (female (*n* = 7); BW ≤ 1000 g (mean 880 g, range 670–1020 g); mean GA 27 weeks (range 23–30 weeks)). FT control group: *N* = 11 (female (*n* = 7). Maternal education mostly ≥college/university degree. Finnish speaking. No further diversity characteristics reported.	PT births at Oulu University Hospital in Northern Finland from October 2003‐April. Healthy FT infants with normal hearing, whose parents volunteered and whose families were geographically attainable, born at Oulu University Hospital August‐November 2003	Congenital/chromosomal anomalies and hearing loss.
van Blankenstein et al. (2024) UK	Retrospective cohort study	Examine the completeness and results of 2‐year‐old neurodevelopmental records in the NNRD and report the applied assessment tools.	PT children: *N* = 41,505 infants (GA 26–29 weeks). *N* = 23,782 with complete NDFU data on NNRD @ 2y. Ethnicity recorded, varied SES, language not reported. No further diversity characteristics reported.	PT infants GA ≤ 30 weeks born January 2008‐31 December 2018 admitted to NIC and survived to discharge from neonatal care.	Missing GA or birth year, children who were deceased prior to assessment
van Noort‐van der Spek et al. (2010) Netherlands	Cross‐sectional study	Gain knowledge about phonological development in young VLBW children with no major neurosensory impairment	VLBW case group: *N* = 20 (female *n* = 13); FT control group: *N* = 20 (female *n* = 13) Broad SES spread, Dutch speaking. No further diversity characteristics reported.	VLBW recruited from the Neonatal Follow‐up Clinic at the Erasmus Medical Center, Sophia Children's Hospital in Rotterdam. FT children: aged 1;11–2;1 years; GA 38–42 weeks; BW≥ 2800 g;	Major neurosensory impairment Neurobiological Risk Score ≥8; oral structure; bilingualism; and multiple birth
van Noort‐van der Spek et al. (2022a) Netherlands.	Prospective cohort study	Identify distinctive profiles of children born VPT at age 2 years based on neurodevelopmental outcomes. Describe the longitudinal course of the profiles at age 2 years.	VPT children *N* = 84 assessed at age 2 years (mean BW 1173 g ± 392 g; mean GA 29 ± 2 weeks). *N* = 61 completed follow‐up assessment at age 10 years. Maternal education ≥high school. Dutch‐speaking. No further diversity characteristics reported.	GA< 32 weeks, admitted to level 3 NICU in Rotterdam, home language Dutch.	Cerebral palsy, severe sensory issues, congenital abnormalities involving speech organs, multiple birth.
van Noort‐van der Spek et al. (2022b) Netherlands.	Prospective cohort study	Present a detailed description of early speech sound production and its trajectories in VPT children from 2–4 years of age.	VPT children *N* = 84 assessed at age 2 years (mean BW 1173 g ± 392 g; mean GA 29 ± 2 weeks). *N* = 63 completed follow‐up assessment at age 4 years. Broad SES. Dutch‐speaking. No further diversity characteristics reported.	GA< 32 weeks, admitted to level 3 NICU in Rotterdam, home language Dutch.	Cerebral palsy, severe sensory issues, congenital abnormalities involving speech organs, multiple birth.
Wolke and Meyer (1999)	Retrospective cohort study	Investigate the expression of VPT children, matched FT controls, and a representative sample of children of the same cohort living in a geographically defined area in the south of Germany.	VPT case group: (*n* = 264) Matched control group (*n* = 264) Normative sample (*n* = 311) inc. 6% PT and 51% male. SES. Ethnicity not reported.	Participants were selected from the Bavarian Longitudinal Study with available assessment recorded data at age 6 years and 3 months.	Children without data at age 6 years 3 months.
Wolke et al. (2008) UK	Retrospective cohort study	To determine whether language and educational problems are specific or due to general cognitive deficits in children born at ≤ 25 weeks' gestation.	PT case group: *N* = 241, assessment age 5;2–7;3 years. FT control group: *N* = 160. No SES or diversity characteristics reported.	All births, between March 1995‐December 1995, GA 20‐25 weeks.	Children born GA ≥25;6 weeks; outside of UK and outside of date range. Children with incomplete/unobtainable data were excluded.

Abbreviations: AGA, average for gestational age; BPD, Bronchopulmonary dysplasia; BW, birth weight in grams (g); CP, cerebral palsy; FT, full term birth (≥37 weeks’ completed gestational age); GA, gestational age in weeks; IVH, intraventricular hemorrhage; LGA, large for gestational age; N, Number of participants; NIC, Neonatal intensive care; NICU, Neonatal Intensive Care Unit; NNRD, National Neonatal Research Database (UK); OME, otitis media with effusion; PCC, Percentage Consonants Correct; PT, preterm birth (≤37 weeks’ completed gestational age); RANOPS, Respiratory and Neurological Outcomes of Children Born Preterm Study; SD, standard deviation; SES, socioeconomic status; SGA, small for gestational age; UK, United Kingdom; USA, United States of America; VLBW, very low birth weight; VPT, Very preterm

* authors’ language choice.

### 3.5 Critical Appraisal

As a scoping review, the aim of this study was not to evaluate the quality of evidence or to generate graded recommendations for practice. Consistent with methodological guidance, no formal critical appraisal was undertaken (Tricco et al. 2018). This review provides an overview of the extent and nature of the evidence, highlights areas of consistency and uncertainty, and identifies priorities for future research (Munn et al. 2018; Peters et al. 2015).

### 3.6 Analysis of the Evidence

A narrative synthesis of the overall study findings is reported. In line with a scoping review methodology, analysis is largely descriptive, with frequency counts relating to the concept and context of studies where appropriate.

## 4 Results

A total of 28 reports met eligibility criteria and were retained for the review (Figure 1). Papers excluded at full text are listed in Supplementary Materials 2. The retained studies included 23 cohort studies (D'Odorico et al. 2011; Rvachew et al. 2005; Törölä et al. 2012a, 2012b; van Noort‐van der Spek et al. 2010) (retrospective, *n* = 14 (D'Odorico et al. 2011; Hong et al. 2026; Imgrund et al. 2023; Jennische and Sedin, 1998; Jennische and Sedin, 1999a, 1999b; Largo et al. 1986; Maggio et al. 2021; Peters et al. 2015; Sanchez et al. 2020; UNICEF 2024; van Blankenstein et al. 2024; Wilson, 2004; Wolke and Meyer, 1999)); prospective, *n* = 9 (Lewis et al. 2002; Luoma et al. 1998; McGillion et al. 2017; Milankov and Mikov, 2009; Oller et al. 1994; Törölä et al. 2012a, 2012b; van Noort‐van der Spek et al. 2022; Wolke et al. 2008) and five cross‐sectional studies (Filgueira de Souza e Souza et al. 2019; Goldfield 2000; Shahramnia et al. 2023; van Noort‐van der Spek et al. 2010) (retrospective, *n* = 2 (Oller et al. 1994; van Noort‐van der Spek et al. 2010); prospective, *n* = 3 (Filgueira de Souza e Souza et al. 2019; Goldfield, 2000; Shahramnia et al. 2023) (Table 2). The following sections describe the sample and methodological characteristics of the retained reports.

### 4.1 Study Characteristics

The publication year range of the retained papers was 1986–2025, with almost a third (*n* = 9) (Aromataris 2020; Brown et al. 1986; Gould et al. 2021; Jennische and Sedin 1999a; Jennische and Sedin 1999b; Luoma et al. 1998; Largo et al. 1990; Oller et al. 1994; Wolke and Meyer 1999) of the reports published before the year 1986 (Goldfield, 2000; Jennische and Sedin, 1999a; van Blankenstein et al. 2024) (Table 2). The papers originated from 14 countries (the UK (Jee et al. 2023; van Blankenstein et al. 2024; Wolke et al. 2008) and The Netherlands (Törölä et al. 2012; van Noort‐van der Spek et al. 2022a; van Noort‐van der Spek et al. 2022b; van Noort‐van der Spek et al. 2010); USA (Brown et al. 1986; Goldfield 2000; Imgrund et al. 2023; Lewis et al. 2002; Oller et al. 1994) and Finland (Luoma et al. 1998; Törölä et al. 2012a; Törölä et al. 2012b); Iran (Shahramnia et al. 2023), Serbia (Milankov and Mikov 2009), Italy (D'Odorico et al. 2011), Brazil (Filgueira de Souza e Souza et al. 2019), Canada (Rvachew et al. 2005), Australia (Sanchez et al. 2020; Holm and Crosbie 2010), Republic of Korea (Hong et al. 2026), Switzerland (Largo et al. 1986; Largo et al. 1990), Germany (Wolke and Meyer 1999) and Sweden (Jennische and Sedin 1998; Jennische and Sedin 1999a, 1999b; Jennische and Sedin 2001)).

FT control groups were included in 23 of the retained studies. Of the remaining five studies, three presented secondary analysis of extant clinical data (Brown et al. 1986; Hong et al. 2026; van Blankenstein et al. 2024) and two undertook longitudinal follow‐up of a PT cohort (van Noort‐van der Spek et al. 2022a, 2022b).

### 4.2 Sample Characteristics

Eight studies reported on the same population of children (Jennische and Sedin 1998; Jennische and Sedin 1999a, 1999b; Jennische and Sedin 2001; Törölä et al. 2012a, 2012b; van Noort‐van der Spek et al. 2022a, 2022b), resulting in a total of 25,759 PT participants across the 28 retained reports (range: 16–23,782). Nine of the retained studies (D'Odorico et al. 2011; Filgueira de Souza e Souza et al. 2019; Goldfield 2000; Imgrund et al. 2023; Oller et al. 1999; Oller et al. 1994; Törölä et al. 2012a, 2012b; van Noort‐van der Spek et al. 2010) included samples < 30 participants and, excluding the study with the largest cohort (van Blankenstein et al. 2024), the average sample size was 73 participants.

PT birth subcategorization followed the World Health Organisation (WHO) (World Health Organisation 2023) published criteria in all except one of the retained studies (D'Odorico et al. 2011). All except one study (Wolke et al. 2008) included VPT children and eight (Aromataris 2020; Filgueira de Souza e Souza et al. 2019; Imgrund et al. 2023; Jee et al. 2023; Jennische and Sedin 1999a, 1999b; Largo et al. 1990; Largo et al. 1986; Munn et al. 2018; Wolke et al. 2008) included participants across the three PT sub‐categories (Figure 2).

**FIGURE 2:**
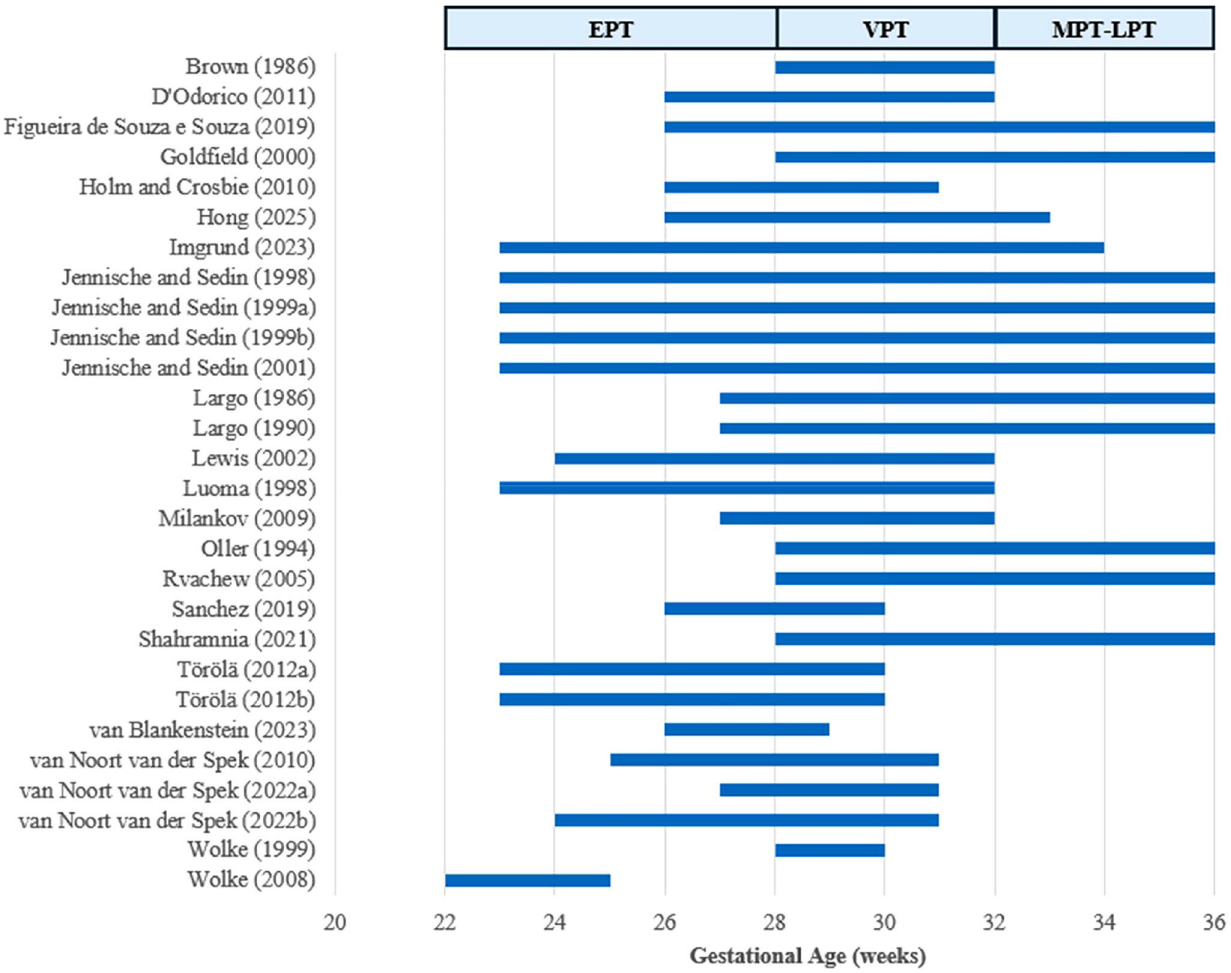
Preterm categories of samples from included studies.

Across the retained reports, the assessment age of participants ranged from 3 months to 12 years (Table 3). The majority (*n* = 22) of the studies assessed children at specific age points (e.g., age 2 years). Six papers assessed participants within an age range (e.g., age 2–4 years) (Brown et al. 1986; Filgueira de Souza e Souza et al. 2019; Holm and Crosbie 2010; Oller et al. 1994; Shahramnia et al. 2023; Wolke et al. 2008). Eleven studies assessed PT children at their corrected age (Brown et al. 1986; D'Odorico et al. 2011; Largo et al. 1986; Rvachew et al. 2005; Sanchez et al. 2020; Törölä et al. 2012a; Törölä et al. 2012b; van Noort‐van der Spek et al. 2022; van Blankenstein et al. 2024; van Noort‐van der Spek et al. 2010), one reported assessment of participants at their chronological age (Hong et al. 2026; Jennische and Sedin 1998; Jennische and Sedin 1999; Jee et al. 2023) and two reported both corrected and chronological ages of participants (Jennische and Sedin 2001; Oller et al. 1994). Fourteen studies failed to specify their applied age criteria (Filgueira de Souza e Souza et al. 2019; Goldfield 2000; Holm and Crosbie 2010; Imgrund et al. 2023; Jennische and Sedin 1998; Jennische and Sedin 1999a, 1999b; Largo et al. 1990; Lewis et al. 2002; Luoma et al. 1998; Milankov and Mikov 2009; Shahramnia et al. 2023; Wolke and Meyer 1999; Wolke et al. 2008).

**TABLE 3:** Analysis and findings.

Author(s)	Methods	Outcome	Analysis	Findings
Brown et al. (1986) USA	Assessment at 9–22m corrected age. Speech ratings completed by SLT and a medical student in three observation contexts (free play, visual stimulation and speech‐language assessment).	Speech Age calculated from binary scores for prevalence of each of 22 speech variables from babble to word combinations. Speech Quotient (Speech Age divided by corrected age).	Mann‐Whitney U test for group differences. Chi square to test association between speech produced across contexts.	Speech delay shown in both groups. PT+IVH infants showed a more severe delay and more variable performance than control group.
D'Odorico et al. (2011) Italy	Phonological assessment at 12 and 18m (Corrected Age)	Mean Babbling Level; phoneme inventory; descriptive analysis of syllable structures; word type count		No group difference on no. utterances at 12m. Significant difference at 18m.
Filgueira de Souza e Souza et al. (2019) Brazil	Phonology assessment at 2–4 years. ABFW—Child Language Test protocol, validated in Brazil. Chronological age vs corrected age not stated.	Presence of atypical PP (syllable reduction, consonantal harmony, fricative stopping, velar backing, palatal backing, velar fronting, palatal fronting, liquid reduction, cluster reduction, final consonant deletion).	Phonological analysis: 14 PP were analyzed according to their occurrence (inc. 10 phonological deviations). Productive PP = occurrence >25% of in each test. NPPP = < 25% occurrence. Association analysis: Mann‐Whitney test for production averages in the phonological processes. Chi‐squared test for association between prematurity and phonological deviation.	A significant number of phonological processes persisting for PT potentially related to prematurity. PT >atypical phonological processes regardless of the degree of prematurity. Systematic PT follow‐up needed to monitor speech sound acquisition over time, to prevent future disorders and avoid late diagnosis.
Goldfield (2000) USA	Chron age vs corrected age not stated. Vowel audio recordings at ages 3,6,9,12 months as part of larger study.	Individual and sequenced vowels	A one way ANOVA indicated that FT infants produced sequences that were significantly longer than those produced by preterm infants, F (1, 16) 4.51, *p* = .041. A two‐way ANOVA with group (PT, FT) and condition (sequential, individual) was conducted on F1 and F2.	FT infants produced mostly sequences of four vowels (*M* = 4.43, *SD* = 1.20), while PT infants produced mostly sequences of three vowels (*M* = 3.68, *SD* = 0.89). The longest sequence produced by a FT infant was seven, and by a PT infant was 6. This analysis indicated there was a greater difference in F2 between vowels produced in sequence versus those produced individually for FT infants than for PT infants. The results suggest a difference between the groups in the way that tongue anterior‐posterior position is varied as vowels are produced in sequence, compared to its variation for individual vowels. The data suggest that the FT infants begin with an open vocal tract and use tongue position to gradually reduce its cross‐sectional area, but that PT infants do just the opposite: they gradually open an initially closed vocal tract. The ability to produce sequences of jaw opening or closure that are coordinated with respiratory expirations and phonation may be delayed in PT infants.
Holm and Crosbie (2010) Australia	DEAP screening assessment aged 5‐12 years.	Speech ability category 0 (average range) or 1 (speech difficulty).	One‐sample *t*‐tests	More PT detected to have speech difficulties on the DEAP screening assessment. The DEAP screener normative sample found 17% of children were identified (including false positives) with speech difficulties in comparison to the 30% PT children identified. DEAP speech skills (*p* < .01). DEAP (speech difficulties) PT *n*=59 (30%) compared with FT *n*=28 (17%)
Hong et al. (2026) Republic of Korea	Retrospective analysis of medical records. Speech sound evaluation at age 3 and 4 years (chronological age) inc. oral–motor structure and functions; U‐TAP (Kim & Shin, 2005), a standardized articulation and phonology test (picture naming) for Korean‐speaking children aged 3–6 years.	PCC score	Pearson correlation analyses. Binary logistic regression analysis to identify predictors of typical speech sound production at age 4y in children with <normal speech sound production at age 3y.	Four speech sound development trajectories were identified: normal (Trajectory I: *n*=21, 26.2%), abnormal (Trajectory II: *n*=41, 51.3%), catch‐up trajectory (Trajectory III: *n*=16, 20%), growing‐into deficit trajectory (Trajectory IV: *n*=2, 2.5%). Significant differences in sex distribution (χ2 = 8.462, *p* = .015) across trajectories. Trajectory II status associated with significantly lower ELQ at age 3y (*p* = .012) and age 4y (*p* = .008), and lower RLQ at age 4y (*p* = .012) compared to those in Trajectory I. No significant differences were observed across trajectories for GA (*p* = .188), BW(*p* = .218), history of BPD (*p* = .190), IVH (*p* = .304). PCC age 3y: significantly correlated with ELQ at age 3y (r = .500, *p* < .01). PCC at age 4y: significantly correlated with RLQ at age 3y (r = .273, *p* < .05). Strong correlation between PCC at age 3y and PCC at age 4y (r =.654, *p* < .01). No statistically significant predictors for achieving typical speech sound production at age 4y.
Imgrund et al. (2023) USA	Chron age vs corrected age not stated. Examiner was an SLT (Imgrund). A naturalistic sample 100 complete utterances) was obtained from each of the participants to assess both conversational speech and language abilities of the children. The examiner used primarily open‐ended prompts and questions and following the child's lead during the collection of the samples (audio recorded). Transcribed by a trained graduate student in speech–language pathology using Phon (version 3.0; Hedlund & Rose, 2019), a software program designed for building and analyzing phonological data.	Three measures of phonological ability were derived from the speech samples: PCC, pMLU, and PWP.	Independent samples t tests were conducted to determine whether the PT and FT groups differed with respect to PCC, pMLU, and PWP. Pearson's correlation coefficients were also calculated to determine the association between speech and language skills.	No significant differences were found between the PT and FT groups on any of the speech variables. Gestational age at birth and birthweight were not statistically significantly correlated with any of the variables obtained from the speech samples (i.e., PCC, pMLU, or PWP). The correlations ranged from essentially no correlation (r = .07 for PCC and birthweight) to a weak correlation (r = −.2 for gestational age at birth and pMLU). PCC and PWP were not significantly associated with any of the language variables in the PT group.
Jennische and Sedin (1998) Sweden	Evaluation at age 6.5years (chronological age) of spontaneous speech (audio recorded) led by an SLT.	SLT evaluation of speech motor function and sound pattern.	Kruskal‐Wallis one‐way ANOVA for group differences. Wilcoxon two‐tailed p test for specific group differences.	PT scored lower than controls in speech motor function and sound patterns.
Jennische and Sedin 1999a) Sweden	Parent interview administered by an SLT when child aged 6.5years (chronological age).	Parent reported presence of babble, age of first words, intelligible speech.	Kruskal‐Wallis one‐way ANOVA for group differences. Wilcoxon two‐tailed p testfor specific group differences.	PT children produced intelligible speech later than FT (*p* < 0.05). FT less likely to babble than VPT or LPT (*p* < 0.05). EPT less likely to babble than VPT and LPT (LPT < EPT, *p* < 0.05; VPT < EPT, *p* < 0.01). Girls more likely to babble than boys. No gender differences for age at first words or intelligibility. Deficits in fine motor function associated with absence of babbling (*p* < 0.001).
Jennische and Sedin (1999b) Sweden	Assessment at age 6.5 years (chronological age). Linguistic skill assessment protocol administered by an SLT.	Speech imitation skills and phoneme inventory.	Kruskal‐Wallis one‐way ANOVA for group differences. Wilcoxon two‐tailed p test for specific group differences.	Compared with 5% of controls, 40% of PT (GA< 32wks) scored < 10th percentile for articulation.
Jennische and Sedin (2001) Sweden	Report results by both chron and corrected age. Assessment by speech pathologist. A, an evaluation of spontaneous speech, B, an assessment of linguistic skills and C, an interview with the parents. Chron/adj age both reported.	Likert scale (0–5) outcome measures for 'speech motor function' and 'sound pattern'.	IWCM group were removed from analyses.	<32week children had poorer speech motor function than matched controls. 35%–45% of PT had poor speech outcomes compared with 20% of FT (matched controls).
Largo et al. (1986) Switzerland	Assessment at (corrected) age 1, 3, 6, 9, 12, 18, 24, 36, 48 and 60m. Parent‐reported language stages. Laboratory observations of language development. Swiss‐German articulation assessment at age 5 years.	Articulation defects (omission or distortions of syllables and words) (count).	Differences in group means were examined using t and Wilcoxon test.	Age 5y: frequency of misarticulations, and no. different syllable errors, was significantly higher for PT girls than FT girls. PT boys more misarticulations more frequently, than FT boys. Most articulation defects occurred more frequently among boys than girls in both groups. FT and PT children who were slow in their early language development had significantly more articulation problems at age 5y. Between 5y and 9y the frequency of articulation defects decreased considerably. Most frequent articulation defects: /s/, /sh/ and /r/.
Largo et al. (1990) Switzerland	Swiss‐German articulation assessment at age 5 years.	Articulation defects (omission or distortions of syllables and words) (count).	Differences in group means were examined using t and Wilcoxon test.	Articulation errors more frequent in PT than FT children and in boys more than girls.
Lewis et al. (2002) USA	Assessed age 8y. GFTA and TOSF.	GFTA percentile score and TOSF scaled score at age 8 years.	ANOVAs and chi‐square for group comparison. To control for Type I errors, alpha levels were determined by Bonferroni corrections	Children with BPD showed reduced articulation skills compared to the other two groups. Articulation in VLBW children did not differ significantly from matched FT controls. BPD group differed from FT but not the VLBW on the oral motor measures
Luoma et al. (1998) Finland	Assessment at age 5 years. Psychologist administered the assessments.	Repetition of phonological sequences and phonologically complex words / non‐words.	Frequency estimates. To assess the statistical differences between PT and control groups, a two‐tailed t test for matched pairs was used when the variables were normally distributed in both of the groups	PT children performed statistically more poorly than controls.
Milankov and Mikov (2009) Serbia	Global Articulation Test (Kostikj and Vladisavljevikj) (3) was used for screening of sound articulation. Oral exam. Chron/adj age note stated.	Presence of speech disorders	The multivariant analyses of variance (MANOVA) was used for scaled data, as well as the discriminative analysis. Amongst univariant procedures, ANOVA *t*‐test, Roy's test, Pearson's coefficient contingency (c), and multiple correlation coefficient (R), were also conducted.	PT children, being a high‐risk group, need to be followed up by age two, and provided timely professional help at pre‐school age, since numerous adverse factors affect their overall development. Sound distortions more common among PT group.
Oller et al. (1994) USA	Parent questionnaire and lab‐based monthly and bi‐monthly speech recordings at ages 0;4 to 1;6 (chronological and corrected ages reported).	Proportion of total speechlike syllables (canonical babbling ratio (CBR), full vowel ratio(FVR) and quasi‐vowel ratio (QVR)) and volubility (utterances per minute).	Four mixed ANOVAs for four dependent variables, canonical babbling ratio (CBR), full vowel ratio (FVR), quasi‐vowel ratio (QVR) and utterances per minute (U/M) were conducted. Each design had two between‐subject factors (SES, term) and one within‐subject factor (age).	FT exhibit the more mature speech behaviour to a reliably greater extent than PT. FT have reliably higher CBRs (*p* < 0–05) and FVRs (*p* < 0–05) and reliably lower QVRs (*p* < 0.05). A reduction in the differences between the groups is noted when corrected age is used instead of chronological age. FT are significantly more likely (chi2 = 8.6, d.f. = 3, *p* < 005) than PT to exceed the criterion of 0–15 CBR.
Rvachew et al. (2005) Canada	**Adjusted age**. Speech assessed at 8, 12 and 18 months of age. 50 to 100 utterances was audio recorded from each infant. An utterance was defined as a non‐cry, non‐reflexive, non‐vegetative vocalization produced within a single breath unit. Meaningful and non‐meaningful utterances were not differentiated. Phonetic transcription combined with the acoustic analyses described above were used to identify each canonical syllable in the sample. Canonical syllables were identified according to criteria developed by Oller and described most recently in Oller (2000). The canonical syllable ratio (CSR) was determined by dividing the total number of canonical syllables identified by the total number of utterances produced.	Canonical syllable babbling ratio; description of underlying variables that determine CSR; measure of vowel space along F2 dimension; expressive vocab size at 18m (parent report)	A detailed examination of intra‐rater, inter‐rater, and test‐retest reliability was conducted for 18 of the recorded samples.	No major differences between HPT and FT groups. CSR difference between FT and HPT not significant. The HPT group achieved a CSR equivalent to that of the FT group by 18 months despite demonstrating a CSR as low as that produced by the BPD group at 8 and 12 months of age. The canonical babbling stage (i.e. CSR.0.20) was achieved by 8 of 13 BPD children, 7 of 9 HPT children, and all 10 FT children at or before 12 months of age. No between‐group differences were observed for the number of consonant phones used at least twice in the context of a syllable containing at least one consonant and a vowel (F(2,29)51.62, p50.22). No between‐group differences were observed for the number of consonant phones used at least twice in the context of a syllable containing at least one consonant and a vowel (F(2,29)51.62, p50.22)
Sanchez et al. (2020) Australia	**Corrected age**. DEAP articulation and phonology subtests at age 3y. Speech assessments administered by SLTs	Continuous variables used for predictor analyses were: percentage of consonants correct standard score, number of consonants produced (including stimulability—a measure of articulation), and number of phonological processes (a measure of phonology). The number of children scoring more than one standard deviation (SD) below the mean on the DEAP Oro‐Motor subtest was also reported.	REDCap used for data entry. STATA for analysis. Group differences (aim 1) and potential predictors (aim 2) of language, speech, and voice outcomes were evaluated via linear regression fitted using generalized estimating equations to account for multiple births or ordered logistic regression clustered for multiple births. Number of consonants produced and number of phonological processes were not normally distributed. Logarithmic transformation did not result in normal distribution, and so robust standard errors were used to compensate for non‐normality. In analysing potential predictors, univariable analysis was conducted first for all predictors, with only significant predictors in the univariable analysis then added to the multivariable analysis. A significance level of *p* < 0.05 was adopted. No adjustments for multiple comparisons were made. Hypothesized predictors were selected on the basis of having been significantly predictive of language, speech, or voice outcomes in previous studies. Sensorineural hearing loss was a planned predictor for analysis but was omitted owing to low numbers (*n*=2). Predictors of interest included sex, gestational age at birth, birthweight z‐score, chronic lung disease (oxygen at 36wks), high social risk (score ≥2 on Social Risk Index), multilingualism, neurodevelopmental diagnosis (as reported by parents), and oromotor feeding impairment at 12 months (defined as failing one or more food challenges on the Schedule for Oral Motor Assessment).	**On univariable analysis, neither PCC score nor phonological processes were associated with gestational age**. Speech sound difficulties were present in 32.7% of the PT group, and 29.2% of the term group. Of the children with speech sound difficulties, 29 (16 PT, 13 term) had both articulation and phonological difficulties; nine (four PT, five term) had phonological difficulties but no articulation difficulties; and 20 (10 PT, 10 term) had articulation difficulties but no phonological difficulties.
Shahramnia et al. (2023) Iran	**Chron/adj age not specified**. The experimenter was an MSc student in Speech Therapy who was experienced in working with children with speech and language impairments. The data of PT participants were collected in the quiet room at AmirAlam Hospital. For FT children, data was obtained in the nursery schools. Within the single assessment session that lasted approximately 45 min and a short break in between, the child's speech was recorded using the audio‐recording device (Model: SONY‐ICD‐UX560F, China) for data transcription. The session was divided into four stages: first, the experimenter communicated with the child using some toys and stickers. Second, he administrated the single word test, and after a short break, the oral‐motor test was conducted. Finally, he administrated the story retelling test. This test was administrated to children aged four years or higher according to the test manual. Persian version of Ages and Stages Questionnaire (ASQ‐I), is a developmental screening tool. Persian test of speech sound production (Ahmadi et al., 2018; 2019). This test consists of two main subscales: (1) single word test, which includes 70 items, and (2) story retelling test, consisting of a story that examines speech sound production of all of the Persian speech sounds. Oral‐motor functions were measured in three main domains involving diadochokinesis, isolated movements, and sequenced movements using the oral‐motor test developed by Zarifian et al. (2014) for Persian speaking children.	Speech sound assessment and oral motor scores.	SPSS. The normal distribution of the data was examined using Kolmogorov Smirnov test. Then, we ran independent sample *t*‐test to compare the total score of single word test, total score of story retelling test, and oralmotor scale in the two groups and Mann Whitney U test to compare speech intelligibility between the two groups. Interrater reliability was assessed using ICC. The effect of sex on total scores of single word and story retelling tests was calculated using independent sample *t*‐test. Furthermore, Spearman's correlation coefficient was applied to measure the correlation between performance of participants in single word and story retelling tests with each of demographic variables including GA, BW, paternal and maternal education. The relationships of speech intelligibility and oral motor measures comprising isolated movements, sequenced movements, and diachocokinetic movements (/patukejk/and/patuku/) were investigated using Spearman's correlation coefficient.	PT children had significantly poorer performance in speech measures, including single word and story retelling tests, compared with FT children. PT had poorer intelligibility rating. The two groups showed statistically significant differences for oral‐motor skills like diadochokinesis for producing patuku, sequenced, and isolated movements.
Törölä et al. (2012) Finland	**Corrected age**. Video recordings for early vocalisations. The PT infants were video‐recorded for the first time at the gestational age of 37 weeks at the hospital. The second recording took place at 40 weeks of gestational age, and after that at home every 2 weeks up to the corrected age of 3 months, and then once a month up to the end of the expansion stage. The first video‐recording of the FT infants took place at the hospital before discharge. The infants were followed at home every second week up to the age of 3 months and after that once a month up to the end of data collection. The total number of recordings was 329. The median number of recordings was 12 for the PT infants and 11 for the FT infants.	An individual skill was accepted when it first appeared. The infant was defined to have reached a certain developmental stage of early vocalization if at least 20% of the vocalization acts belonged to it (Nathani et al., 2006). In some infants that 20% consisted of only one vocalization type, in others it may have consisted of five different vocalization types belonging to the stage. The time lag between the first appearance of a vocalization and the time to reach the 20% criterion was reported in both groups. Because the vocalizations in the phonation stage are reflexive by nature, the calculation was started since the first skills from the primitive articulation stage were heard. The number of utterances produced during each video‐recording was calculated in order to define whether there was a quantitative difference in vocalization between the PT and FT infants. The ages of emergence of vocalization skills are presented as medians and the ages that describe the skills of the PT infants are all given in corrected ages.	SLT analysed the data. The vocalization of the PT and FT infants was analyzed quantitatively according to the categorical stages created by Oller. A descriptive analysis of all the vocalizations produced by the infants was performed. An experienced speech and language therapist (SLT) analyzed 10% of the randomly chosen vocalizations of the infants. The inter‐rater agreement in analyzing the vocalizations was 97% in the PT and 100% in the FT infants. In defining individual vocalization acts, the inter‐rater agreement between the first author and the SLT was 96% in the PT and 97% in the FT infants. Because the sample size was quite small and distributions of most of the variables were skewed, the Mann–Whitney U test was used in order to compare the ages of entering stages and the number of skills the infants failed to produce at each stage between PT and FT infants.	The PT infants entered the primitive articulation stage later than the FT infants and failed to produce more skills during that stage. According to this sample, there was no difference in entering the expansion stage, but the PT infants failed to produce more skills than the FT infants. The number of vocalization acts varied differently by age between the groups. There was wider age variation among the PT infants in attaining the various skills included in each stage of vocalization. In the primitive articulation and expansion stages they developed fewer skills compared with the FT infants,
Törölä et al. (2012) Finland	Corrected age. Video recordings for early vocalisations. The PT infants were video‐recorded for the first time at the gestational age of 37 weeks at the hospital. The second recording took place at 40 weeks of gestational age, and after that at home every 2 weeks up to the corrected age of 3 months, and then once a month up to the end of the expansion stage. The first video‐recording of the FT infants took place at the hospital before discharge. The infants were followed at home every second week up to the age of 3 months and after that once a month up to the end of data collection. The total number of recordings was 329. The median number of recordings was 12 for the PT infants and 11 for the FT infants.	Oller's (2000) model was used to describe the development of the vocalization. The vocalizations were categorized based on the perceptual auditory analysis of the video‐recordings. The infant was defined as having reached a certain developmental stage if at least 20% of vocalization acts could be categorized as characteristic of a certain stage. Canonical babbling (including variegated), first word phase. A detailed descriptive analysis of the vocalization was made by defining the syllable types and combinations and other vocalization heard and building subgroups of the stages defined by Oller (2000).	A qualified speech and language therapist (SLT) studied the theory of preverbal vocalization and consequently conducted an analysis of the infants’ vocalizations separately. The first author knew who was a PT or a FT infant, but the SLT, who double‐checked the vocalizations for inter‐rater agreement, was blinded. The inter‐rater agreement concerning the categorization of vocalization between the first author and the SLT was 100%, while in defining the vocalization manners, syllables and syllable combinations, the inter‐rater agreement was 90.2% in the PT and FT groups. Because of the small sample size and because the distributions of most variables were skewed, the non‐parametric Mann–Whitney U‐test was used in order to compare the ages of entering stages and the number of skills that the infants failed to produce at each stage between the PT and FT infants. Video‐recording durations varied by infant and by recording session, thus vocalization frequency was constituted by dividing the number of utterances by the duration of the recording. Vocalization frequency was observed from the age of 6 months onwards, when the infants first entered the canonical stage, up to the first word. A mixed model was used to assess the age‐dependent difference between the vocalization of PT and FT infants (McCulloch & Searle, 2001). In addition to infant group, age and their interaction, squared age was included in the model as a fixed explanatory variable to allow for a quadratic trend in vocalization by age. Interaction between quadratic age and infant group was not required in the model, thus the final model was restricted to having an equal quadratic age coefficient for both groups. Infant was treated as a random explanatory variable. Residual variance was allowed to vary by age. Statistical analyses were performed using the IBM SPSS statistical software (version 19.0, SPSS Inc.,Chicago, IL, USA).	The development of preverbal vocalization before variegated babbling did not differ between the groups. Instead, the PT infants failed to produce more different kinds of canonical syllable types than the FT infants. However, they showed a larger variance of variegated babbling skills and remained in the babbling phase longer before reaching the first meaningful word compared with the FT infants. Following the onset of canonical babbling, the PT infants produced fewer vocalizations than the FT infants and they reached the first word later than the FT infants.
van Blankenstein et al. (2024) UK	Corrected age. Data extraction from NNRD	Presence of a 2‐year neurodevelopmental assessment record in the NNRD, use of standardised assessment tools, results of functional 2‐year neurodevelopmental assessments (visual, auditory, neuromotor, communication, overall development).	SAS V.9.4 and Stata V.16.0 software. Index of Multiple Deprivation (IMD) decile, a socioeconomic measure, as derived using maternal postcode at the time of delivery.	23,782 had a 2‐year neurodevelopmental assessment recorded. ∼13% had < 5 meaningful words, vocalisations or signs and ∼26% had < 10. The majority (≥87%) of infants reviewed were assessed using functional (non‐validated) neurodevelopmental tools; however, only half had an assessment using a validated, standardised tool. Rates of impairment were higher for the earlier gestation group across all domains, and there was no improvement seen over time.
van Noort‐van der Spek et al. (2010) Netherlands	Corrected age at 2y	Phonological development was assessed (Dutch standard assessment) by the number of acquired consonants, number of phonological error patterns, and phonological mean length of utterance (pMLU).	SPSS. We used Mann–Whitney U tests to compare continuous variables between the VLBW and term group. A two‐tailed *p* value of less than 0.05 was considered significant. To investigate whether group differences on phonological outcomes persist after correction for developmental and behavioural outcomes, (backwards) linear regression analyses were performed using the method of least squares. Variables with a *p* value of greater than 0.20 in the (backwards) linear regression analyses or variables that were strongly correlated with another variable were not included in the final linear regression models.	VLBW children had significantly fewer acquired consonants (median 9, *p* = 0.02) and a significantly lower phonological mean length of utterance (pMLU; median 4.1, *p* < 0.01) than term children (median acquired consonants 10, median pMLU 5.0).
van Noort‐van der Spek et al. (2022a) Netherlands.	Data obtained from assessments at 2 years (corrected age) and 10 years.	At age 2, speech production was defined by the number of acquired, syllable initial consonants measured by the “Fonologische Analyse van het Nederlands,” the Dutch standard assessment of phonological development in children (Beers, 1995). number of acquired consonants was derived from a speech sample obtained from 20 min of child–parent play interaction. By convention, a consonant was considered acquired if it was attempted at least three times in meaningful words and the percentage of correct production was ≥75%. In the absence of norm‐referenced data we considered six or less acquired consonants as abnormal. Abnormal speech production was defined as six or less acquired consonants based on a spontaneous speech sample of at least 50 different word realizations or a spontaneous speech sample of less than 50 words combined with a delayed word production (i.e., a score less than 1.5 SD below the mean) based on the parent checklist or the standardized test. Non‐classifiable speech production was defined as six or less acquired consonants based on analysis of a speech sample of less than 50 words combined with a normal word production score (i.e., a score more than 1 SD below the mean) on the parent checklist and the standardized test.	The statistical analyses were performed using IBM SPSS Statistics version 25 and R version 3.6. Student's *T*‐test, Pearson's chi‐square test or Fisher's exact test was used to compare perinatal and demographic factors between the study group and the group of non‐participating children and between each profile. The outcome variables were tested for normality using the Kolmogorov–Smirnov test, with *p* < .05 indicating that the tested variable distribution differed from a normal distribution. Continuous outcome measures were compared across the clusters using univariate analysis of variance with the Tukey post hoc test (for normally distributed data) or the Kruskal–Wallis test with the Dunn–Bonferroni post hoc test (for non‐normally distributed data). Normally distributed data are presented as mean ± standard deviation. Data that were not normally distributed are presented as median with interquartile range. Regarding the outcome measure for speech production at age 2, inter‐rater reliability was established. An independent experienced clinical linguist re‐transcribed the spontaneous language of 13 randomly selected children (15%) and measured the number of acquired consonants for each child. A good reliability was found. The average measure ICC was .872with a 95% confidence interval from .581 to .961; F(12,12) = 7.817, *p* < .01. Tukey's test for non‐additivity showed no interaction; F(1,12) = 1.027, *p* = .33.	A multidisciplinary profile‐oriented approach might be important in children born VPT to gain insight in which of these children are at risk for long‐term adverse neurodevelopmental outcomes and to improve counselling and provide targeted treatment for at risk children.

Author(s)	Methods	Outcome	Analysis	Findings
van Noort‐van der Spek et al. (2022b) Netherlands.	**Corrected age at 2y but not specified at 4y**. The VPT children underwent a comprehensive multidisciplinary assessment at 2 years of corrected age of cognitive, motor, speech, language, behavior, and auditory function. For this study, only the speech, language, and auditory function outcomes obtained at this age were included. At 4 years of age, the VPT children underwent a follow‐up assessment of speech, language, and auditory function. The speech and language assessments were performed in the same clinical setting at the Erasmus MC Sophia Children's Hospital, Rotterdam, by a clinical specialist with more than 10 years of experience in diagnosing speech and language problems in children.	**Age 2 years**: The **primary speech sound production outcome** was defined as the **number of acquired consonants** (NAC) measured by the Fonologische Analyse van het Nederlands, the Dutch standard assessment of phonological development in children (Beers, 1995). **Secondary outcomes**: PCC, defined by the number of correctly produced consonants divided by all consonants produced and multiplied by 100. The PCC is computed by averaging the score over 25 meaningful words (Shriberg et al., 1997). The PCC index establishes four degrees of severity: mild (more than 85%), mild–moderate (65%–85%), moderate– severe (50%–64%), and severe (below 50%; Shriberg et al., 1997). **Phonological mean length of utterance (PMLU)**. **Age 4 years:** The primary speech sound production outcome was defined as the **PCCI**, which was calculated by dividing the number of correctly produced consonants in syllable‐initial position by the total number of consonants elicited. PCCI scores were compared to the norm data obtained from the picture naming task of the CAI (van Haaften et al., 2019). **RedClus**, which was defined by the percentage of reduction of initial consonant clusters from two consonants to one divided by the total number of initial consonant clusters of two consonants. Percentage consonants and consonant clusters correct based on 90% norm, which was defined as the percentage correct produced consonants and consonant clusters of those acquired in 90% of the typically developing children at 4 years of age (Stes & Elen, 1991). Percentage consonants and consonants clusters correct based on 75% norm, which was defined as the percentage corrected produced consonants and consonants clusters of those acquired in 75% of the typically developing children at 4 years of age (Stes, 1991).	SPSS. Pearson's chi‐square test (or Fisher's exact test; in case of expected cell frequencies of < 5) and Student's t test were used to compare perinatal and socioenvironmental characteristics between the participating group and the nonparticipating group of VPT children at the assessment at 2 years of age and the follow‐up assessment at 4 years of age, respectively. Chi‐square tests and ANOVA with Tukey's post hoc tests were used to compare characteristics and language outcomes among the distinctive speech trajectory groups.	Speech sound production was found to be abnormal in 49% of the VPT children at 2 years of age and in 19% at 4 years of age. Compared to the general population, an alarmingly high proportion of VPT children showed speech production problems at 2 years of age. About half of these children showed persistent speech problems at 4 years of age. Moreover, these problems were associated with expressive language problems at the age of 4 years. Early speech production, defined by the number of acquired consonants at 2 years of age, significantly predicted the word production score at 4 years of age and the sentence production score at 4 years of age.
Wolke and Meyer (1999)	Speech ax (novel articulation picture test) age 6y administered by postgraduate clinical psychologists and psychometric assistants.	Item responses scored as either correct or incorrect (either replacement, omission or no response). WI, WM, WF positions scored individually and total score across subtests. Consensus rating on quality of speech index consisted of five items: intelligibility of speech, voice quality, melody of speech, volume modulation of speech, and tempo.	Comparisons between VPT and matched controls. They included parametric analyses using sequential ANOVA for interval data assessing mean differences between VPT and controls, (allowing for sex and SES).	VPT had poorer word articulation outcomes (VPT, 24.1%; controls, 4.9%, *P* < 0.001). for example, ‘Sch’ sounds (< 10th centile: VPT, 25.7%; controls, 7.6%, *P* < 0.001), WI sounds (< 10th centile: VPT, 18.0%; controls, 7.6%, *P* < 0.01) or WM sounds <10th centile: VPT, 18.0%; controls, 4.2, *P* < 0.001).
Wolke et al. (2008) UK	Children assessed at age 6 years. Paediatrician and developmental psychologists completed the assessments. 19 item word imitation as part of the PLS and PAT.	Articulation screen score. PAT score (below 10th centile as clinical presentation).	Ch‐square for categorical outcomes. *T*‐tests for continuous outcomes.	EPT children 3.8x more likely to have serious articulation impairment. Boys were >2x likely than girls to have serious articulation impairment. EPT group ≤4x likely to have phonological awareness difficulties at age 6 years.

Abbreviations: ABFW, Child Language Test; ANOVA, One Way Analysis of Variance; BPD, Bronchopulmonary dysplasia; CSR, Canonical Syllable Ratio; EPT, Extremely preterm birth; FT, Full term; HPT, healthy preterm; M, Mean; MANOVA, multivariant analyses of variance; NAC, number of acquired consonants; NPPP, number of productive phonological processes; PAT, phonological articulation test; PCCI, percentage consonants correct syllable‐initial; PMLU, phonological mean length of utterance; PP, phonological processes; PPC, Percentage of consonants correct; PT, Preterm; PWP, phonological word proximity; RANOPS, Respiratory and Neurological Outcomes of Children Born Preterm Study; SD, Standard Deviation; SLT, Speech and language therapist; UK, United Kingdom; USA, United States of America; VLBW, Very low birth weight; VPT, Very preterm born.

### 4.3 Speech Outcome Measures

Outcome measures, measurement tools and assessors varied across the included studies (Table 3) [Supplementary Materials 3–5].

Thirteen different speech sound related outcome variables were reported across the 28 included studies [Supplementary Material 4]. Phonetic inventory (D'Odorico et al. 2011; Jennische and Sedin 1999; Sanchez et al. 2020; Shahramnia et al. 2023; Wolke and Meyer 1999; van Noort‐van der Spek et al. 2022; van Noort‐van der Spek et al. 2010; Wolke et al. 2008) and phonological processes (Filgueira de Souza e Souza et al. 2019; Jennische and Sedin 1998; Jennische and Sedin 2001; Largo et al. 1990; Milankov and Mikov 2009; Sanchez et al. 2020; Shahramnia et al. 2023; van Noort‐van der Spek et al. 2010; Wolke et al. 2008) were the most common, followed by babble and early syllable structure (D'Odorico et al. 2011; Hong et al. 2026; Jennische and Sedin 2001; Törölä et al. 2012a, 2012b; van Blankenstein et al. 2024; van Noort‐van der Spek et al. 2022a), speech motor function (Hong et al. 2026; Jennische and Sedin 1998; Jennische and Sedin 2001; Jennische and Sedin 1999a; Luoma et al. 1998; Sanchez et al. 2020; Shahramnia et al. 2023) and first words (D'Odorico et al. 2011; Jennische and Sedin 1999b; Largo et al. 1986; Oller et al. 1999; Törölä et al. 2012a; van Blankenstein et al. 2024). Less commonly used outcomes included percentage consonants correct (PCC) (Hong et al. 2026; Imgrund et al. 2023; Sanchez et al. 2020; van Noort‐van der Spek et al. 2022a), articulation errors (Jennische and Sedin 2001; Largo et al. 1986; Lewis et al. 2002; Luoma et al. 1998; Milankov and Mikov 2009; Sanchez et al. 2020; Shahramnia et al. 2023; Wolke and Meyer 1999), vowels (Goldfield 2000; Oller et al. 1994; Rvachew et al. 2005), phonological awareness (Johnson et al. 2008) and (Wolke et al. 2008), intelligibility (Jennische and Sedin 1999; Jee et al. 2023). Two studies (Brown et al. 1986; Holm and Crosbie 2010) reported derived novel outcomes. Brown et al. assigned each child an estimated ‘speech age’ based on binary ratings (+ present /—not present) of 22 speech variables (Brown et al. 1986). Holm and Crosbie applied a binary ‘speech ability category’ (average range/speech difficulty) based on the outcome of the Diagnostic Evaluation of Articulation and Phonology (DEAP) Diagnostic Screen (Holm and Crosbie 2010).

Fifteen of the 28 studies reported audio/video recording of spontaneous speech, with subsequent data transcription (D'Odorico et al. 2011; Goldfield 2000; Imgrund et al. 2023; Jennische and Sedin 2001; Jennische and Sedin 1998; Jennische and Sedin 1999a; Largo et al. 1986; Oller et al. 1994; Rvachew et al. 2005; Sanchez et al. 2020; Shahramnia et al. 2023; Törölä et al. 2012a, 2012b; van Noort‐van der Spek et al. 2022b; van Noort‐van der Spek et al. 2010). Fourteen studies used standardised clinical assessments (Filgueira de Souza e Souza et al. 2019; Holm and Crosbie 2010; Hong et al. 2026; Largo et al. 1990; Lewis et al. 2002; Luoma et al. 1998; Milankov and Mikov 2009; Sanchez et al. 2020; Shahramnia et al. 2023; van Blankenstein et al. 2024; van Noort‐van der Spek et al. 2022a, 2022b; van Noort‐van der Spek et al. 2010; Wolke et al. 2008), thirteen studies reported using non‐standard assessment protocols (Brown et al. 1986; D'Odorico et al. 2011; Goldfield 2000; Imgrund et al. 2023; Jennische and Sedin 1999a; Largo et al. 1986; Wolke and Meyer 1999), and one study employed a parent‐reported measure (Jennische and Sedin 1999).

Across the 28 studies, speech outcome assessment was undertaken by individuals with varied backgrounds, although in a third (*n* = 9) of retained studies the assessor was unspecified (D'Odorico et al. 2011; Filgueira de Souza e Souza et al. 2019; Goldfield 2000; Largo et al. 1986; Luoma et al. 1998; Milankov and Mikov 2009; Oller et al. 1994; van Blankenstein et al. 2024). Speech and language therapists completed assessments in more than half of the studies (*n* = 16) (Brown et al. 1986; Hong et al. 2026; Imgrund et al. 2023; Jennische and Sedin 2001; Jennische and Sedin 1998; Jennische and Sedin 1999a, 1999b; Lewis et al. 2000; Peters et al. 2015; Rvachew et al. 2005; Sanchez et al. 2020; Tricco et al. 2018; Törölä et al. 2012a, 2012b; van Noort‐van der Spek et al. 2022a, 2022b), with psychologists (Wolke and Meyer 1999; Wolke et al. 2008), paediatricians (Largo et al. 1990; Wolke et al. 2008) and students (Brown et al. 1986; Shahramnia et al. 2023) each used in two studies.

### 4.4 RQ: What Are the Characteristics of Speech Sound Development in Preterm‐born Children?

The majority of studies in this review report findings compared to FT children or published developmental norms. The characteristics of speech sound development in PT children aged 3 months to 6 years from studies which assessed PT participants at a specified age point (e.g., age 2 years) are reported in Supplementary Materials 5 and 6 and summarized in the following sections. Five studies examined speech sound development across a broad age range. Oller et al. (1994) found that in the first 18 months of life, PT children produced more immature speech patterns than their FT peers. Compared with developmental norms, Brown et al. (1986) reported delays in speech development among PT children aged 11–19 months, and more marked outcomes for those with history of IVH. Filgueira de Souza e Souza et al. (2019) reported that PT children aged 2‐to‐4 years were six times (95% CI:1.34;26.81) more likely to develop a “phonological deviation” than a FT control. Shahramnia et al. (2023) found that children aged 3‐to‐5 years were less intelligible and had poorer oromotor skills than FT children. Holm and Crosbie (2010) assessed children age 5–12 years and found that twice as many PT children had speech difficulties compared to FT children (PT: *n* = 59, 30%; FT: *n* = 28, 17%).

#### 4.4.1 PT Speech Sound Development Age 0–2 Years

Goldfield (2000) reported that, compared with FT children, PT participants presented with shorter and different vowel sequences, while Töröla et al. 2012a observed fewer skills in the early primitive articulation and expansion stages of babble development among PT children. In a related study by the same authors, canonical babbling was observed from age 8 months, along with fewer vocalizations, and slower increase in vocalizations, as part of a longer babbling phase and protoword period (Törölä et al. 2012). Jennische and Sedin (1999) reported that absence of babbling was more common among EPT than VPT or MLPT children. Rvachew et al. (2005) reported 78% of healthy PT children producing canonical babbling by 12‐months‐old, compared with 100% of FT controls (Rvachew et al. 2005). At this age, D'Odorico et al. (2011) observed similar amounts of babble and word production between PT and FT participants, although the PT group produced fewer utterances with reduplicated or variegated syllables (D'Odorico et al. 2011). They found that PT children had more vowel‐only utterances and 50% fewer stable phones at this age compared with FT controls (D'Odorico et al. 2011).

Two studies (D'Odorico et al. 2011; Rvachew et al. 2005) reported that, at 18‐months‐old, PT children produced equivalent amounts of canonical syllables as FT children, with some reduction in reduplicated or variegated syllable production observed (D'Odorico et al. 2011). D'odorico et al. noted that PT children produced fewer utterances and had smaller vocabularies with fewer different word types, compared with FT children (D'Odorico et al. 2011).

Van Noort van der Spek et al. (2010) and Van Noort van der Spek et al. (2022b) found that, at 2‐years‐old, PT children had fewer consonants and more error patterns in their speech, compared with FT children (van Noort‐van der Spek et al. 2022a; van Noort‐van der Spek et al. 2010). One study reported that half (49%, *n* = 41) of PT participants had fewer than six syllable initial consonants at age 2 years and 31% (*n* = 26) achieved PCC scores in the moderate‐severe or severe range, indicating clinical concern (van Noort‐van der Spek et al. 2022). Reduced vocabulary at age 2 years was evident for PT children (D'Odorico et al. 2011; van Blankenstein et al. 2024), and one study reported that 13% (*n* = 3,092) of PT children had five or fewer meaningful words, signs or vocalizations and 26% (*n* = 6,183) had 10 or fewer words or signs (van Blankenstein et al. 2024).

#### 4.4.2 PT Speech Sound Development Age 3–5 Years

Mixed findings were reported for pre‐school aged PT children (Supplementary Materials 7). One study reported low PCC scores (>‐2 standard deviations) for almost a third (28.75%, *n* = 23) of children at age 3 years, increasing to almost half (46.25%, *n* = 37) of 4‐year‐olds born preterm (Hong et al. 2026). PT 3‐and‐4‐year‐olds were found to have similar speech sound development as FT born participants (Imgrund et al. 2023; Sanchez et al. 2020). Evidence of increased sound distortions among 4‐year‐old PT children (Milankov and Mikov 2009), and evidence of fewer consonants and consonant clusters at this age were described (van Noort‐van der Spek et al. 2022), along with a high proportion of children who obtained PCC scores below the typical range (Hong et al. 2026). Wolke and colleagues found that, at age 5 years, school‐aged PT children had four times greater risk of articulation impairment and phonological difficulties (Jennische and Sedin 2001; Wolke et al. 2008). For children at the same age, Largo et al. (1986) reported higher rates of speech sound errors among PT children compared to FT children with multiple errors more common among PT children. In contrast, Luoma and colleagues (1998) reported poor speech articulation outcomes among only the PT children who had neurological disabilities.

#### 4.4.3 PT Speech Sound Development Age 6+ Years

Wolke and Meyer (1999) examined data from the Bavarian Longitudinal Study and found that, at age 6 years, a quarter (*n* = 62) of VPT children made articulation errors, compared with only 8% (*n* = 26) of FT children. In their follow‐up of PT children in Sweden, Jennische and Sedin reported poorer speech motor outcomes for children at age 6.5 years (Jennische and Sedin 2001). The same study reported that between 60%–70% (LPT *n* = 50, EPT/VPT *n* = 45) PT children scored grade 4 (slight deviation) or below for speech sound production compared with 55% (*n* = 39) FT. MLPT children scored higher for accuracy of articulatory positions than VPT and EPT children (Jennische and Sedin 1999). Lewis and colleagues reported that, at the age of 8 years, VPT children had comparable speech to their FT peers when those with BPD were excluded from the analysis. Largo et al. (1990) reported that around one in seven PT children made articulation errors at age 9 years.

### 4.5 Speech Sound Difficulties in PT Children

Over one third (*n* = 10) studies reported criteria for identifying participants with speech sound difficulties. Sanchez et al. (2020) applied the scoring criteria from the DEAP (Dodd et al. 2006) and identified 31% (*n* = 24) PT children as having speech difficulties at age 3 years. Using the same assessment, Holm and Crosbie (2010) applied the DEAP Diagnostic Screen (Dodd et al. 2006) and, using a simplified classification approach, categorized children as either ‘average range’ or ‘speech difficulty’. Consistent with Sanchez et al. (2020), they also identified speech difficulties in 30% of PT children (*n* = 59) compared with 17% (*n* = 28) FT children. Van Noort van der Spek (2022a, 2022b) defined abnormal speech production as ≤ 6 acquired consonants and found that 49% (*n* = 41) VPT children had atypical speech sound development at age 2 years. Based on assessment with the Urimal Test of Articulation and Phonology (U‐TAP) (Kim and Shin 2005), Hong et al. (2026) applied a PCC cut‐off score of ≤ 2 s.d. and found that 71% (*n* = 57) of PT two‐year‐olds and 53% (*n* = 43) of PT four‐year‐olds presented with SSD (Goldman and Fristoe 1986). Jennische and Sedin (1998), Jennische et al. (1992) graded children's spontaneous speech at age 6.5 years and identified that, compared with 2.5% (*n* = 1) of FT controls, 19.7% of PT children had either an obvious articulation difficulty or no speech at all (≤grade 3). In a related study (Jennische and Sedin 1999), the authors found that 25% (*n* = 33) of MLPT and 44% of children born < 32 weeks (VPT *n* = 24, 44.2%; EPT *n* = 12, 44.9%) scored below the 10^th^ percentile threshold for typical speech development at age 6.5 years, compared with 5% (*n* = 4) of FT controls. Similarly, Lewis et al. (2002) found that 15% (*n* = 24) of PT children aged 8 years had a speech disorder compared with 3% (*n* = 3) FT controls, based on the criteria (percentile < 16) from the Goldman‐Fristoe Test of Articulation (GFTA) (Goldman and Fristoe 1986). In their study of EPT children, Wolke et al. (2008) found that, at age 6.5 years, children born ≤ 25 weeks had four times greater odds (OR = 4.4; 95% CI 3–7) of meeting DSM‐IV (American Psychiatric Association 1994) criteria for phonological disorder compared with their FT peers.

The retained studies varied in the reporting of participants with a history of SLT intervention. In their assessment of speech outcome in PT children at age 10 years, van Noort‐van der Spek et al. (2022) reported 32/61 PT participants had received SLT, though the nature and timing of this was not specified. Holm and Crosbie (Holm and Crosbie 2010) collected data on previous SLT intervention for their sample but examined this only for language and literacy outcomes, not for speech. Hong et al. (2026) reported that 81% (*n* = 65) of participants had received language intervention but did not further specify what this entailed. In their study, over half (*n* = 38) of PT children who received prior intervention had an ‘abnormal trajectory’ while 20% (*n* = 13) showed a ‘normal trajectory’ (Hong et al. 2026). Groups were not differentiated elsewhere in the analysis. Jennische and Sedin (2001, 1999a, 1999b), Aromataris (2020) excluded FT controls who had received prior SLT, but these data were not reported for the PT group (Jennische and Sedin 1998; Jennische et al. 1992). Lewis et al. (2002) reported ‘enrolment in SLT classes’ but did not specify nature of this (e.g., speech VS language therapy). Between group analysis of SLT input was undertaken but it is unclear whether SLT input status was included in analysis of speech outcome at age 8 years. Thirteen studies (Brown et al. 1986; Imgrund et al. 2023; Jennische and Sedin 1999a; Jennische and Sedin 1998; Jennische and Sedin 1999a, 1999b; Largo et al. 1990; Lewis et al. 2002; Luoma et al. 1998; Oller et al. 1994; Sanchez et al. 2020; van Blankenstein et al. 2024; van Noort‐van der Spek et al. 2022a; Wolke and Meyer 1999) sampled children within an age range likely to be eligible for SLT intervention, but collection and/or analysis of these data was not reported.

### 4.6 Specific Speech Sound Errors

A small number of studies reported specific speech sound errors among PT children. Filgueira de Souza e Souza et al. (2019) reported that within their sample of 20 Brazilian Portuguese speaking participants, PT children produced more syllable reduction, consonant harmony, velar backing and liquid reduction than expected, processes the authors report should be absent by age 3;6, however the children were assessed between the ages of 2 and 4 years, and individual age at assessment of the 20 PT children is not provided. In their study of 24 Italian speaking children, D'Odorico et al. (2011) found PT children had fewer fricative sounds at age 12 months compared to FT children. Wolke and Meyer (1999) examined data from 264 VPT German speaking children and found that, compared to their FT counterparts, they made significantly more errors with palatal fricative /ʃ/.

### 4.7 Trajectories for PT Speech Sound Development

Two longitudinal studies (Munn et al. 2018; Wolke and Meyer 1999) reported evidence of speech development trajectories among preterm born children. van Noort‐van der Spek et al. (2022) identified four different trajectories of speech development among VPT Dutch speaking children (*n* = 61) in their study between the ages of 2 and 4 years. All but one of the 32 children who presented with age‐appropriate speech at age 2 years retained this classification at age 4 years, with the outlier classified as ‘growing into deficit’. Of the VPT children (*n* = 19) who presented with atypical speech at age 2 years, 10 had persistent speech difficulties at age 4 years (abnormal trajectory’), while nine made progress at age 4 and were classified as having a ‘catch‐up trajectory’. The application of these trajectories was further investigated in a recent study by Hong and colleagues (2026) in South Korea, who assessed children across the full spectrum of prematurity at ages 3 and 4 years. They found half of PT children presented with ‘abnormal trajectory’ (*n* = 41, 51%), of which 41% were VPT (*n* = 17), much higher than the 16% of VPT children identified with this trajectory in the Dutch study (van Noort‐van der Spek et al. 2022). ‘Abnormal trajectory’ was the most common presentation (EPT *n* = 10, 42%; LPT *n* = 14, 58%), followed by ‘normal’ (*n* = 21, 26%: EPT *n* = 7, 29%; VPT *n* = 9, 28%; LPT *n* = 5, 21%) and then ‘catch‐up trajectory’ (*n* = 16, 20%: EPT *n* = 6, 25%; VPT *n* = 6, 18%; LPT *n* = 4, 17%). Only two of the 80 children assessed presented as ‘growing into deficit’, similar to the findings of the Dutch study (van Noort‐van der Spek et al. 2022).

## 5 Discussion

The findings of this review synthesize existing evidence that speech sound development in PT children differs from speech development in FT children. Some studies reported that, compared with FT children, those born PT have poorer performance on a range of key clinical speech measures. Evidence of reduced verbal output and reduced complexity of speech patterns in the early stages of speech development is reported (D'Odorico et al. 2011; Goldfield 2000; Rvachew et al. 2005; Törölä et al. 2012a, 2012b; van Noort‐van der Spek et al. 2022a, 2022b; van Noort‐van der Spek et al. 2010). The findings indicate that PT children may be more likely to achieve key speech milestones later than FT children (Brown et al. 1986; D'Odorico et al. 2011; Largo et al. 1986; Oller et al. 1994; Törölä et al. 2012a, 2012b).

In the general population, delayed babbling and first word production are recognized risk factors for future SSD later in childhood (Bishop and Adams 1990; Lang et al. 2019; McGillion et al. 2017; Overby et al. 2012; Oller et al. 1998; Oller et al. 1999; Wren et al. 2016). In contrast, the near‐unanimous findings of this review suggest that delays in speech milestones during the first 12–18 months may be characteristic of early speech development in PT children. What is not yet clear is the extent to which these variations constitute ‘typical’ speech development among PT children, and which factors may indicate atypical speech development in this population. For example, once a PT child has achieved a given milestone, albeit at a later age to their FT peers, do they go on to develop ‘typical’ speech which enables them to participate in their daily activities? Although evidence from two studies (Hong et al. 2026; van Noort‐van der Spek et al., 2022a) included in this review provides encouraging insights into potential trajectories of speech sound development in PT children, the small sample sizes and methodological heterogeneity undermine the replicability of the findings and constrain their applicability to clinical practice. The complexity and heterogeneity of multifaceted individual factors, which influence speech sound development, even before birth, challenge the aspiration that it might one day be possible to predict a child's outcome (van Noort‐van der Spek et al., 2010); Davis and Bedore, 2013). The potential to leverage neuroplasticity to support early speech development and mitigate poor outcomes through early identification of PT children at highest risk is an emerging focus in clinical research (DeMaster et al. 2019; Sahinoglu et al. 2026; Vandormael et al. 2019)

Evidence of speech sound development in PT children over time is needed, with outcomes measured early in childhood through to school age and ideally beyond. Only eight of the papers included in this scoping review reported longitudinal studies, three (D'Odorico et al. 2011; Hong et al. 2026; Törölä et al. 2012a, 2012b) of which reported final outcomes at around 18 months, at a time when speech sound development is known to be highly variable (Broomfield and Dodd 2004; Dodd et al. 2003; Grunwell 1987; Stoel‐Gammon and Dunn 1985; Vihman 1996). Two papers (Hong et al. 2026; van Noort‐van der Spek et al. 2022a) explored outcomes at pre‐school age and identified different trajectories for PT speech development (typical, atypical, catch‐up and growing‐into‐deficit) between the ages of 2 and 4 years. One of the research teams (van Noort‐van der Spek et al. 2022b), followed up with the same participants at 10‐years‐old, but the aggregation of speech sound outcomes with language measures meant the specificity for speech development was lost. While the early evidence from these studies is encouraging, the small sample and group sizes constrain the impact of the findings. Further research is required with larger samples and outcomes measured throughout childhood to inform how these trajectories evolve for the duration of speech development. This is particularly important given recently published evidence on the impact of persistent speech sound disorder on adolescent mental health and wellbeing (McAllister et al. 2023) and the growing body of evidence which suggests that the neurological effects of preterm birth persist into adulthood (Stegmann‐Woessner et al. 2025; Wolke et al. 2019).

The prevalence of speech sound assessment by qualified and experienced speech and language therapists reported in this review is encouraging and enhances the clinical relevance and credibility of the findings. In addition, the majority of outcome variables reported in the retained reviews (e.g., PCC score, early speech and language milestones, phonetic inventory and phonological processes) represent established measures, and mirror those commonly applied in both the wider speech sound development literature and in routine clinical practice around the world (Harding et al. 2024).

The findings of this review add weight to the growing call for robust, large‐scale longitudinal studies of PT children to inform clinical support beyond current survival‐focused intensive care (Axford et al. 2025). Research into typical and atypical speech sound development in PT children is needed to inform identification of those at highest risk of SSD. This review supports the need for speech outcomes to be measured from the first months of life through to school age and early adolescence to elucidate developmental trajectories for different groups of PT children. The learning gained from this future research will underpin the evaluation of current clinical speech sound assessment tools to determine their appropriacy for use with PT children. Necessary refinements or potential new measures can then be developed to support accurate and reliable assessment of PT speech sound development based on normative data linked to gestational age.

### 5.1 Clinical Implications

The findings of this review strengthen the current evidence base for the consistent collection of comprehensive data, including gestational age and early speech milestones during initial clinical assessment as part of routine care. Anecdotal evidence from clinical services indicates that, within and across SLT services, data on gestational age and prematurity is not consistently collected at initial assessment. This means that the potential influence of prematurity is not always considered as part of a child's wider risk profile for SSD. Furthermore, if gestational age is not collected, then the SLT is not able to analyse a child's assessment scores using their corrected age. This review found inconsistency in the reporting and use of age measures for PT children. Due to the magnitude of the differences between corrected and uncorrected scores in the first years of life (Gould et al. 2021), evidence indicates that correction of age is important for evaluating the test scores of younger children, particularly those born before 29 weeks’ completed gestation (D'Agostino 2010; Wilson‐Ching et al. 2014; Wilson 2004). A comprehensive review by Wilson and Cradock (2004) recommended that clinicians and researchers report both corrected and chronological ages (Wilson 2004). Only two of the included studies (Jennische and Sedin 2001; Oller et al. 1994) reported both of these factors, highlighting an established weakness in the majority of research regarding speech and language development and outcomes in PT children. The findings of this review demonstrate the importance of considering both the child's corrected and chronological age in PT children, particularly in early childhood, when assessing any aspect of neurodevelopment (D'Agostino 2010; Gould et al. 2021; Wilson 2004).

Intervention planning should be informed by an understanding that PT children may present with different patterns and rates of speech sound acquisition. Regular monitoring is essential to track developmental progress and distinguish delayed but progressing development from persistent SSD. Such approaches can support timely adjustment of intervention intensity and goals as the child's profile evolves, meaning care is responsive to changing clinical need. Clinicians can use this synthesised evidence to inform clinical practice with regard to the assessment, monitoring and clinical care of PT children with SSD.

### 5.2 Clinical Research Implications

From a clinical research perspective, the findings of this review suggests that speech sound assessment norms and intervention evidence, which are often derived predominantly from FT populations, may warrant further evaluation regarding their suitability for PT children. Research is needed to determine whether observed differences represent delays, different trajectories, or may be indicators of persistent SSD. Clinical research should include sufficiently powered PT cohorts, to enable the development of population‐specific normative data, and the evaluation of intervention effectiveness within PT populations. Generating this evidence is essential to ensure future clinical recommendations accurately reflect the developmental profiles and needs of PT children (National Institute for Health and Care Excellence (NICE) 2017, Storey 2019) and support the implementation of early intervention approaches. These developments have the potential to reduce the longer‐term health and social care costs associated with persistent developmental difficulties (Geelhoed et al. 2020).

### 5.3 Limitations of Retained Studies

Evidence synthesis within this review was challenged by a number of factors, most notably a high degree of methodological heterogeneity across the retained studies.

The range in sample sizes across the studies is an important consideration in the interpretation of the results.

Outcome measurements varied across the papers and some studies included derived scores. Holm et al. (Holm and Crosbie 2010) reported only the presence/absence of speech difficulty from the DEAP Diagnostic Screen and, despite the identification of high proportion of children with difficulties, further detailed assessment with the DEAP was not reported. Brown et al. (Brown et al. 1986) assessed 22 speech variables, including vowels and consonants, but reported only the ‘Speech Age’ derived from the presence of the most advanced skill.

Not all studies reported prior SLT input for participants. The majority of studies which did not report these data included children under age 2 and so clinical input communication for this age group would not be routinely expected (Hong et al. 2026; Holm and Crosbie 2010; Jennische and Sedin 1998; Milankov and Mikov 2009)

Differences in definitions of atypical speech sound development challenged the comparison of findings across the retained studies. In addition, some studies focused only on PT speech development, while others compared PT and FT speech sound development.

A range of assessment approaches was observed. Findings from studies which report outcomes from specific age points or smaller age ranges (e.g., 12 months) present potentially the greatest utility in informing future research and developments to practice. This is because they provide more precise estimates of development at given stages and reduce the age‐related heterogeneity that arises from aggregated data. For example, Holm and Crosbie (2010) reported speech outcomes on 169 PT children across a seven‐year age range, including primary and secondary school aged children. Speech development in a child aged 5 years is not directly comparable to that of a 12‐year‐old.

Studies varied in application and reporting of chronological age vs corrected age, which is an important consideration for neurodevelopmental research (Gould et al. 2021; Rickards et al. 1989; Wilson‐Ching et al. 2014). Published evidence advocates for the use of corrected age in research, particularly in the early years, due to the persistent bias resulting from preterm birth (D'Agostino 2010; Gould et al. 2021; Wilson 2004).

Diversity characteristics beyond sex (ethnicity, socioeconomic measure and primary language) were reported to some extent in all but seven (Goldfield 2000; Hong et al. 2026; Jennische and Sedin 1998; Jennische and Sedin 1999a, 1999b; Milankov and Mikov 2009; Wolke et al. 2008) studies (Table 2). Precise and consistent reporting of these data is required if robust research practices are to be followed.

### 5.4 Strengths and Limitations of this Review

This study is a scoping review and therefore not intended to develop graded recommendations for practice or policy, nor undertake formal critical appraisal of the included studies (Peters et al. 2015; Tricco et al. 2018). However, the study provides important preliminary data and maps out the patterns of delayed and atypical speech sound development that occur at varying ages. These findings provide a useful platform for future targeted studies of the evidence, undertaken as systematic reviews or primary longitudinal studies, focused on specific gestational ages and using consistent outcome measures.

A limitation of the present review is that only papers written in English were retained, potentially overlooking valuable research published in other languages. This must be considered when applying the findings to clinical practice, as it may affect the comprehensiveness and generalizability of the review's findings. However, the included papers represented geographical spread across 14 countries. The retained papers reported on a range of languages beyond English, which is a further strength of this paper due to the evidence for cross‐linguistic patterns observed in child speech development (Sharynne and Kathryn 2018). This review included papers where children with medical conditions common among the PT population, such as BPD and IVH were recruited. However, papers were excluded where the population included PT children with conditions known to impact speech sound development, such as CP and Down Syndrome. This approach was taken to avoid, where possible, under‐representation of children born at the earliest extremes of prematurity.

## 6 Conclusions

This review provides evidence that there are differences in how PT and FT children develop sounds for speech from birth through to childhood. These differences have implications for PT children's language development, education, social integration and wellbeing as well as wider life chances. These findings are of relevance to health, care and education professionals, as well as carers and families. Future large‐scale, longitudinal research with PT children is needed to inform identification of a profile of risk factors and atypical characteristics of speech development in this group of children, so that early identification as part of routine neurodevelopmental follow‐up can take place.

## Funding

Dr Sam Burr held a National Institute for Health and Care Research (NIHR) Development and Skills Enhancement Award (NIHR305708), through which Dr Harding's authorship of this review was also funded.

## Conflicts of Interest

The authors have no conflicts of interest to disclose.

## Role of Funder/Sponsor

The NIHR had no role in the design and conduct of the study.

## Article Summary

This scoping review brings together current knowledge on the differences in speech sound development between preterm and term‐born children.

## Supporting information




**Supporting Information**: jlcd70347‐sup‐0001‐SuppMat.docx

**Supporting Information**: jlcd70347‐sup‐0002‐SuppMat.docx

**Supporting Information**: jlcd70347‐sup‐0003‐SuppMat.docx

**Supporting Information**: jlcd70347‐sup‐0004‐SuppMat.docx

**Supporting Information**: jlcd70347‐sup‐0005‐SuppMat.docx

**Supporting Information**: jlcd70347‐sup‐0006‐SuppMat.docx

**Supporting Information**: jlcd70347‐sup‐0007‐SuppMat.docx

## Data Availability

Data sharing is not applicable to this article as no new data were created or analyzed in this study.
