# Supplementary material for: Characteristics of Speech Sound Development in Preterm‐Born Children: A Scoping Review
Source: Int J Lang Commun Disord. 2026 Oct 16;61(6):e70347. doi: 10.1111/1460-6984.70347 (PMC13674721; doi:10.1111/1460-6984.70347)
Supplement: Supplementary file 1 [file JLCD-61-0-s001.docx]

**Supplementary Material 2. Excluded papers**

| **Reference** | **Exclusion reason** |
| --- | --- |
| Abolfotouh MA, Al Saif S, Altwaijri WA, Al Rowaily MA. Prospective study of early and late outcomes of extremely low birthweight in Central Saudi Arabia. BMC Pediatr. 2018 Aug 22;18(1):280. doi: 10.1186/s12887-018-1248-y. PMID: 30134865; PMCID: PMC6106812. | No relevant outcomes reported (speech sound / intelligibility) |
| Adanikin A, Lawlor DA, Pell JP, Nelson SM, Smith GCS, Iliodromiti S. Association of birthweight centiles and early childhood development of singleton infants born from 37 weeks of gestation in Scotland: A population-based cohort study. PLoS Med. 2022 Oct 11;19(10):e1004108. doi: 10.1371/journal.pmed.1004108. PMID: 36219591; PMCID: PMC9553050. | No relevant outcomes reported (speech sound / intelligibility) |
| Adegbiji WA, Olajide GT, Olubi OO, Ali A, Adeniyi AM. Communication disorders among children in a developing country, Nigeria. International Journal of Biomedical Science. 2019;15(4):98-103. doi: 10.59566/IJBS.2019.15098 | Outcome not explored in relation to GA |
| Agarwal PK, Xie H, Sathyapalan Rema AS, Meaney MJ, Godfrey KM, Rajadurai VS, Daniel LM. Concurrent validity of the ages and stages questionnaires with Bayley Scales of Infant Development-III at 2 years - Singapore cohort study. Pediatr Neonatol. 2024 Jan;65(1):48-54. doi: 10.1016/j.pedneo.2023.03.013. Epub 2023 Jul 18. PMID: 37544806. | Gestational age of participants not reported |
| Ahn SH, Kim SA. Assessment of Preterm Infants Using the Bayley-III Scales in Korea. Ann Rehabil Med. 2017 Oct;41(5):843-850. doi: 10.5535/arm.2017.41.5.843. Epub 2017 Oct 31. PMID: 29201824; PMCID: PMC5698672. | No relevant outcomes reported (speech sound / intelligibility) |
| Aiken C. Long-term neurodevelopmental outcomes in small babies. Obstetrics, Gynaecology & Reproductive Medicine. 2020;30(6):163-166. doi: 10.1016/j.ogrm.2020.03.002 | No relevant outcomes reported (speech sound / intelligibility) |
| Alhaidary A, Rvachew S. Cross-linguistic differences in the size of the infant vowel space. Journal of Phonetics. 2018;71:16-34. doi: 10.1016/j.wocn.2018.07.003 | Gestational age of participants not reported |
| Amayreh MM, Dyson AT. (2000). Phonetic inventories of young Arabic-speaking children. *Clinical Linguistics & Phonetics*, *14*(3), 193–215. https://doi.org/10.1080/026992000298823 | Gestational age of participants not reported |
| Amr Rey O, Sanchez-Delgado P, Salvador Palmer M, Cibrian Ortiz De Anda RM, Paredes Gallardo V. Exploratory study on the prevalence of speech sound disorders in a group of Valencian school students belonging to 3rd grade of infant school and 1st grade of primary school. PsicologÃ­a Educativa. 2022;28(2):195-207. doi: 10.5093/psed2022a1 | Gestational age of participants not reported |
| André V, Durier V, Henry S, Nassur F, Sizun J, Hausberger M, Lemasson A. The vocal repertoire of preterm infants: Characteristics and possible applications. Infant Behav Dev. 2020 Aug;60:101463. doi: 10.1016/j.infbeh.2020.101463. Epub 2020 Jun 28. PMID: 32610173. | No relevant outcomes reported (speech sound / intelligibility) |
| Araujo DM, Cabrera Santos DC, Marconi Pinheiro Lima MC. Cognitive, language and motor development of infants exposed to risk and protective factors. Int J Pediatr Otorhinolaryngol. 2020 Nov;138:110353. doi: 10.1016/j.ijporl.2020.110353. Epub 2020 Sep 5. PMID: 32920449. | No relevant outcomes reported (speech sound / intelligibility) |
| Arora NK, Nair MKC, Gulati S, Deshmukh V, Mohapatra A, Mishra D, Patel V, Pandey RM, Das BC, Divan G, Murthy GVS, Sharma TD, Sapra S, Aneja S, Juneja M, Reddy SK, Suman P, Mukherjee SB, Dasgupta R, Tudu P, Das MK, Bhutani VK, Durkin MS, Pinto-Martin J, Silberberg DH, Sagar R, Ahmed F, Babu N, Bavdekar S, Chandra V, Chaudhuri Z, Dada T, Dass R, Gourie-Devi M, Remadevi S, Gupta JC, Handa KK, Kalra V, Karande S, Konanki R, Kulkarni M, Kumar R, Maria A, Masoodi MA, Mehta M, Mohanty SK, Nair H, Natarajan P, Niswade AK, Prasad A, Rai SK, Russell PSS, Saxena R, Sharma S, Singh AK, Singh GB, Sumaraj L, Suresh S, Thakar A, Parthasarathy S, Vyas B, Panigrahi A, Saroch MK, Shukla R, Rao KVR, Silveira MP, Singh S, Vajaratkar V. Neurodevelopmental disorders in children aged 2-9 years: Population-based burden estimates across five regions in India. PLoS Med. 2018 Jul 24;15(7):e1002615. doi: 10.1371/journal.pmed.1002615. PMID: 30040859; PMCID: PMC6057634. | Gestational age of participants not reported |
| Athari P, Dey R, Rvachew S. Vocal imitation between mothers and infants. Infant Behav Dev. 2021 May;63:101531. doi: 10.1016/j.infbeh.2021.101531. Epub 2021 Feb 11. PMID: 33582572. | No preterm participants |
| Babies Who Hear Their Own Babbling May Babble More. ASHA Leader. 2016;21(2):17. doi: 10.1044/leader.RIB2.21022016.17 | Wrong publication type |
| Ballot, D. E., Rothberg, A. D., & Katz, B. J. (1992). Speech and hearing problems in a high-risk population. South African Medical Journal, 1992; 82(1), 23 EP - 26. | No relevant outcomes reported |
| Bartolomei B, Ferretti E, Peretti A, De Angelis F, Pannunzi CP, D'Ascenzo R, Carnielli VP. Neurodevelopmental outcome of preterm infants of less than 32 weeks gestation: A 6-year retrospective cohort study of the Marche Neonatal Network. Italian Journal of Pediatrics. 2019;45. doi: 10.1186/s13052-019-0729-4 | Wrong publication type |
| Bauer, H. R., & Robb, M. P. (1992). The ethologic model of phonetic development: III. The phonetic product. Clinical Linguistics and Phonetics, 1992; 6(4), 317 EP - 327. | Gestational age of participants not reported |
| Beers M, Rodenburg-Van Wee M, Gerrits E. Whole-word measures and the speech production of typically developing Dutch children. Clin Linguist Phon. 2019;33(12):1149-1164. doi: 10.1080/02699206.2019.1596163. Epub 2019 Apr 15. PMID: 30987469. | Gestational age of participants not reported |
| Ben-David A. (2006). On the acquisition of Hebrew #sC onsets. *Journal of Multilingual Communication Disorders*, *4*(3), 205–217. https://doi.org/10.1080/14769670601110531 | Gestational age of participants not reported |
| Benders T, Altvater-Mackensen N. Before the word: Acquiring a phoneme inventory. Early word learning. 2018. doi: 10.4324/9781315730974-1 | No preterm participants |
| Bergelson E, Soderstrom M, Schwarz IC, Rowland CF, Ramírez-Esparza N, R Hamrick L, Marklund E, Kalashnikova M, Guez A, Casillas M, Benetti L, Alphen PV, Cristia A. Everyday language input and production in 1,001 children from six continents. Proc Natl Acad Sci U S A. 2023 Dec 26;120(52):e2300671120. doi: 10.1073/pnas.2300671120. Epub 2023 Dec 12. PMID: 38085754; PMCID: PMC10756310. | Gestational age of participants not reported |
| Blake, J., & de Boysson-Bardies, B. (1992). Patterns in babbling: a cross-linguistic study. Journal of Child Language, 1992; 19(1), 51 EP - 74. | Gestational age of participants not reported |
| Borisova MA, Belova EG, Belova LV. Delayed speech development in preschool children. causes, principles of treatment. Archives of Disease in Childhood. 2017;102:A148-A149. doi: 10.1136/archdischild-2017-313273.386 | Wrong publication type |
| Borjon JI, Abney DH, Yu C, Smith LB. Infant vocal productions coincide with body movements. Dev Sci. 2024 Jul;27(4):e13491. doi: 10.1111/desc.13491. Epub 2024 Mar 3. PMID: 38433472; PMCID: PMC11161311. | Gestational age of participants not reported |
| Bouza H, Anatolitou F, Lipsou N, Petropoulou C, Dassopoulou M, Naoum E, Pilitsidou E, Anagnostakou M. Follow up of very low birth weight neonates: Neurodevelopmental outcome and intervention programs. Early Human Development. 2010;86:S87-S88. doi: 10.1016/j.earlhumdev.2010.09.230 | Wrong publication type |
| Boyer J, Flamant C, Boussicault G, Berlie I, Gascoin G, Branger B, N'Guyen The Tich S, Rozé JC. Characterizing early detection of language difficulties in children born preterm. Early Hum Dev. 2014 Jun;90(6):281-6. doi: 10.1016/j.earlhumdev.2014.03.005. Epub 2014 Apr 13. PMID: 24726534. | No relevant outcomes reported (speech sound / intelligibility) |
| Broomfield J, Dodd B. (2004). The nature of referred subtypes of primary speech disability. Child Language Teaching and Therapy, 20(2), 135-151. https://doi.org/10.1191/0265659004ct267oa (Original work published 2004) | Gestational age of participants not reported |
| Cabbage KL. The Role of Speech Perception in Persistent Speech Sound Disorder. Perspectives on School-Based Issues. 2015;16(20:18-24. doi: 10.1044/sbi16.2.18 | Unable to source |
| Calame, A., Fawer, C. L., Claeys, V., Arrazola, L., Ducret, S., & Jaunin, L. Neurodevelopmental outcome and school performance of very-low-birth-weight infants at 8 years of age. European Journal of Pediatrics, 1986; 145(6), 461-466. https://doi.org/doi:https://dx.doi.org/10.1007/BF02429043 | Participants has multiple additional diagnosis |
| Canault M, Yamaguchi N, Kern S. (2022). Early Development of Syllable Structure in French. *Language Learning and Development*, *19*(4), 420–436. https://doi.org/10.1080/15475441.2022.2138401 | No preterm participants |
| Canault M, Yamaguchi N, Paillereau N, Krzonowski J, Roy JP, Dos Santos C, Kern S. Syllable duration changes during babbling: a longitudinal study of French infant productions. J Child Lang. 2020 Nov;47(6):1207-1227. doi: 10.1017/S030500092000015X. Epub 2020 Apr 29. PMID: 32347197. | No preterm participants |
| Carson CP, Klee T, Carson DK, Hime LK. Phonological profiles of 2-year-olds with delayed language development: predicting clinical outcomes at age 3. Am J Speech Lang Pathol. 2003 Feb;12(1):28-39. doi: 10.1044/1058-0360(2003/050). PMID: 12680811. | Gestational age of participants not reported |
| Carter A, Gerken L. Do children's omissions leave traces? J Child Lang. 2004 Aug;31(3):561-86. doi: 10.1017/s030500090400621x. PMID: 15612390. | Gestational age of participants not reported |
| Carter A, Maher C, Carlisle H, Broom M. Growth and development follow up: Making a difference in a baby's future! Journal of Paediatrics and Child Health. 2014;50:75. | Wrong publication type |
| Cassar C, McCabe P, Cumming S. "I still have issues with pronunciation of words": A mixed methods investigation of the psychosocial and speech effects of Childhood Apraxia of Speech in adults. Int J Speech Lang Pathol. 2023 Apr;25(2):193-205. doi: 10.1080/17549507.2021.2018496. Epub 2022 Jan 15. PMID: 35034534. | Gestational age of participants not reported |
| Castro Conde JR, González Campo C, González González NL, Reyes Millán B, González Barrios D, Jiménez Sosa A, Quintero Fuentes I. Assessment of neonatal EEG background and neurodevelopment in full-term small for their gestational age infants. Pediatr Res. 2020 Jul;88(1):91-99. doi: 10.1038/s41390-019-0693-0. Epub 2019 Dec 10. PMID: 31822017; PMCID: PMC7326702. | No relevant outcomes reported (speech sound / intelligibility) |
| Cattani A, Bonifacio S, Fertz M, Iverson JM, Zocconi E, Caselli MC. Communicative and linguistic development in preterm children: a longitudinal study from 12 to 24 months. Int J Lang Commun Disord. 2010 Mar-Apr;45(2):162-73. doi: 10.3109/13682820902818870. PMID: 22748029. | No relevant outcomes reported (speech sound / intelligibility) |
| Ceron MI, Simoni SN, Keske-Soares M. Phonological acquisition of Brazilian Portuguese: Ages of customary production, acquisition and mastery. Int J Lang Commun Disord. 2022 Mar;57(2):274-287. doi: 10.1111/1460-6984.12689. Epub 2021 Dec 26. PMID: 34957652. | Gestational age of participants not reported |
| Charkaluk ML, Rousseau J, Benhammou V, Datin-Dorrière V, Flamant C, Gire C, Kern S, Pierrat V, Kaminski M, Marret S. Association of Language Skills with Other Developmental Domains in Extremely, Very, and Moderately Preterm Children: EPIPAGE 2 Cohort Study. J Pediatr. 2019 May;208:114-120.e5. doi: 10.1016/j.jpeds.2018.12.007. Epub 2019 Mar 14. PMID: 30879730. | No relevant outcomes reported (speech sound / intelligibility) |
| Charollais A, Stumpf MH, De Quelen R, Rondeau S, Pasquet F, Marret S; Perinatal Network of Haute-Normandie. Delayed language development at two years of age in very preterm infants in the Perinatal Network of Haute-Normandie. Early Hum Dev. 2014 Dec;90(12):891-2. doi: 10.1016/j.earlhumdev.2014.10.001. Epub 2014 Oct 31. PMID: 25463837. | No relevant outcomes reported (speech sound / intelligibility) |
| Chaudhari, S., Bhalerao, M. R., Chitale, A., Pandit, A. N., & Nene, U. Pune low birth weight study--a six year follow up. Indian pediatrics, 1999; 36(7), 669 EP - 676. https://doi.org/doi: | No relevant outcomes reported |
| Chen, C.-R. A language development scale of young children. Dissertation Abstracts International Section A: Humanities and Social Sciences, 1991; 59(8), 2943-2943. | No relevant outcomes reported |
| Chen IC, Chen CL, Wong MK, Chung CY, Chen CH, Sun CH. Clinical analysis of 1048 children with developmental delay. Chang Gung medical journal. 2002;25(11):743-750. | No relevant outcomes reported (speech sound / intelligibility) |
| Chen LM, Kent RD. Consonant-vowel co-occurrence patterns in Mandarin-learning infants. J Child Lang. 2005 Aug;32(3):507-34. doi: 10.1017/s0305000905006896. PMID: 16220633. | Gestational age of participants not reported |
| Cheong J, Spittle A, Potter C, Walsh J, Anderson P, Doyle LW. Delayed language development at 2 years in children born moderate to late preterm. Journal of Paediatrics and Child Health. 2015;51:42. doi: 10.1111/jpc.12884-3 | Wrong publication type |
| Cheong JL, Doyle LW, Burnett AC, Lee KJ, Walsh JM, Potter CR, Treyvaud K, Thompson DK, Olsen JE, Anderson PJ, Spittle AJ. Association Between Moderate and Late Preterm Birth and Neurodevelopment and Social-Emotional Development at Age 2 Years. JAMA Pediatr. 2017 Apr 3;171(4):e164805. doi: 10.1001/jamapediatrics.2016.4805. Epub 2017 Apr 3. PMID: 28152144. | No relevant outcomes reported (speech sound / intelligibility) |
| Cheong JL, Olsen J, Opie G, Stewart A, Burnett A, Anderson P, Spittle A, Doyle L. Two-year neurodevelopment in extremely preterm infants: Changes over 25 years in victoria. Journal of Paediatrics and Child Health. 2020;56:8. doi: 10.1111/jpc.14831 | Wrong publication type |
| Choo LCW, Daniel LM. An audit of developmental follow-up in infants with birth weights between 1,251 and 1,500g. Proceedings of Singapore Healthcare. 2010;19:S324. doi: 10.1177/20101058100190S201 | Wrong publication type |
| Christensen R, Chau V, Synnes A, Guo T, Grunau RE, Miller SP. Preterm Neurodevelopmental Trajectories from 18 Months to 4.5 Years. J Pediatr. 2023 Jul;258:113401. doi: 10.1016/j.jpeds.2023.113401. Epub 2023 Apr 3. PMID: 37019331. | No relevant outcomes reported (speech sound / intelligibility) |
| Cochet H, Byrne RW. Communication in the second and third year of life: Relationships between nonverbal social skills and language. Infant Behav Dev. 2016 Aug;44:189-98. doi: 10.1016/j.infbeh.2016.07.003. Epub 2016 Jul 21. PMID: 27450099. | Gestational age of participants not reported |
| Craig, H. K., Evans, J. L., Meisels, S. J., & Plunkett, J. W. Linguistic production abilities of 3-year-old children born premature with low birth weight. Journal of Early Intervention, 1991; 15(4), 326-337. https://doi.org/doi:https://doi.org/10.1177/105381519101500402 | No relevant outcomes reported |
| Creighton D, Lodha A, Majnemer A, Moddemann D, Church P, Lee D, Lefebvre F. Bayley-III outcomes at 18-21 months corrected age for a Canadian cohort of infants born <29 weeks gestation. Paediatrics and Child Health (Canada). 2014;19(6):e59-e60. | Wrong publication type |
| Culloty AM, O'Toole C, Gibbon FE. Longitudinal Study of Expressive Language and Speech of Twins at 3 and 5 Years: Outgrowing a Twinning Effect. J Speech Lang Hear Res. 2019 Jul 15;62(7):2425-2437. doi: 10.1044/2019_JSLHR-L-18-0333. Epub 2019 Jun 27. PMID: 31251680. | Gestational age of participants not reported |
| Curtin S, Zamuner TS. Understanding the developing sound system: interactions between sounds and words. Wiley Interdiscip Rev Cogn Sci. 2014 Sep;5(5):589-602. doi: 10.1002/wcs.1307. Epub 2014 Aug 13. PMID: 26308747. | Wrong publication type |
| Cuttini M, Ferrante P, Mirante N, Chiandotto V, Fertz M, Dall'Oglio AM, Coletti MF, Johnson S. Cognitive assessment of very preterm infants at 2-year corrected age: performance of the Italian version of the PARCA-R parent questionnaire. Early Hum Dev. 2012 Mar;88(3):159-63. doi: 10.1016/j.earlhumdev.2011.07.022. Epub 2011 Sep 8. PMID: 21862246. | No relevant outcomes reported (speech sound / intelligibility) |
| Dall'oglio AM, Rossiello B, Coletti MF, Bultrini M, DE Marchis C, Ravà L, Caselli C, Paris S, Cuttini M. Do healthy preterm children need neuropsychological follow-up? Preschool outcomes compared with term peers. Dev Med Child Neurol. 2010 Oct;52(10):955-61. doi: 10.1111/j.1469-8749.2010.03730.x. Epub 2010 Aug 16. PMID: 20722666. | No relevant outcomes reported (speech sound / intelligibility) |
| Davis, B. L., & MacNeilage, P. F. Acquisition of correct vowel production: a quantitative case study. Journal of Speech and Hearing Research, 1990; 33(1), 16-27. https://doi.org/doi:https://dx.doi.org/10.1044/jshr.3301.16 | Gestational age of participants not reported |
| Davis, B. L., & MacNeilage, P. F. The articulatory basis of babbling. Journal of Speech and Hearing Research, 1995; 38(6), 1199 EP - 1211. https://doi.org/doi:https://dx.doi.org/10.1044/jshr.3806.1199 | Gestational age of participants not reported |
| Davis B, VAN DER Feest S, Yi H. Speech sound characteristics of early words: influence of phonological factors across vocabulary development. J Child Lang. 2018 May;45(3):673-702. doi: 10.1017/S0305000917000484. Epub 2017 Dec 4. PMID: 29198209. | Gestational age of participants not reported |
| De Boysson-Bardies, B., Halle, P., Sagart, L., & Durand, C. A crosslinguistic investigation of vowel formants in babbling. Journal of Child Language, 1989; 16(1), 1 EP - 17. | Gestational age of participants not reported |
| de Goes FV, Meio MDBB, de Mello RR, Morsch D. Evaluation of neurodevelopment of preterm infants using Bayley III scale. Revista Brasileira de Saude Materno Infantil. 2015;15(1):47-55. doi: 10.1590/S1519-38292015000100004 | Wrong publication type |
| de Koning HJ, de Ridder-Sluiter JG, van Agt HM, Reep-van den Bergh CM, van der Stege HA, Korfage IJ, Polder JJ, van der Maas PJ. A cluster-randomised trial of screening for language disorders in toddlers. J Med Screen. 2004;11(3):109-16. doi: 10.1258/0969141041732229. PMID: 15333268. | No relevant outcomes reported (speech sound / intelligibility) |
| De Maesschalck D, Van Der Velde K, Hollemans T, Dereu I, Cloet E, Jansen AC. Neurodevelopmental profiles in preterm children attending primary school. Developmental Medicine and Child Neurology. 2014;56:50-51. doi: 10.1111/dmcn.12485 | Wrong publication type |
| Demuth K, McCullough E. The longitudinal development of clusters in French. J Child Lang. 2009 Mar;36(2):425-48. doi: 10.1017/S0305000908008994. Epub 2008 Oct 21. PMID: 18937877. | Gestational age of participants not reported |
| Demuth K. Crosslinguistic perspectives on the development of prosodic words. Lang Speech. 2006;49(Pt 2):129-35. doi: 10.1177/00238309060490020101. PMID: 17037119. | Wrong publication type |
| Demuth K. Interactions between lexical and phonological development: cross-linguistic and contextual considerations--a commentary on Stoel-Gammon's 'Relationships between lexical and phonological development in young children'. J Child Lang. 2011 Jan;38(1):69-74. doi: 10.1017/S0305000910000449. Epub 2010 Oct 18. PMID: 20950503. | Wrong publication type |
| Demuth K. Perception, production, and individual differences. Applied Psycholinguistics. 2018;39(4):735-741. doi: 10.1017/S0142716418000176 | Wrong publication type |
| DePaolis RA, Vihman MM, Nakai S. The influence of babbling patterns on the processing of speech. Infant Behav Dev. 2013 Dec;36(4):642-9. doi: 10.1016/j.infbeh.2013.06.007. Epub 2013 Jul 31. PMID: 23911593. | Gestational age of participants not reported |
| Dinnsen DA, O'Connor KM. Typological predictions in developmental phonology. J Child Lang. 2001 Oct;28(3):597-628. doi: 10.1017/s0305000901004780. PMID: 11797541. | Wrong publication type |
| Duncan AF, Matthews MA. Neurodevelopmental Outcomes in Early Childhood. Clin Perinatol. 2018 Sep;45(3):377-392. doi: 10.1016/j.clp.2018.05.001. PMID: 30144844. | Wrong publication type |
| Durrant C, Wong HS, Cole TJ, Hutchon B, Collier L, Wright A, George C, De Haan M, Huertas Ceballos A. Developmental trajectories of infants born at less than 30 weeks' gestation on the Bayley-III Scales. Arch Dis Child Fetal Neonatal Ed. 2020 Nov;105(6):623-627. doi: 10.1136/archdischild-2019-317810. Epub 2020 May 3. PMID: 32366516. | No relevant outcomes reported (speech sound / intelligibility) |
| Dworkin JP, Meleca RJ, Stachler RJ. More on the role of the mandible in speech production: clinical correlates of Green, Moore, and Reilly's (2002) findings. J Speech Lang Hear Res. 2003 Aug;46(4):1016-9; author reply 1020-1. doi: 10.1044/1092-4388(2003/079). PMID: 12959477. | Wrong publication type |
| Ejiri, K. Relationship between rhythmic behavior and canonical babbling in infant vocal development. Phonetica, 1998; 55(4), 226 EP - 237. https://doi.org/doi: | No preterm participants |
| Ejiri K, Masataka N. Co-occurrence of preverbal vocal behavior and motor action in early infancy. Developmental Science. 2001;4(1):40-48. doi: 10.1111/1467-7687.00147 | No preterm participants |
| Elgen I, Sommerfelt K, Ellertsen B. Cognitive performance in a low birth weight cohort at 5 and 11 years of age. Pediatr Neurol. 2003 Aug;29(2):111-6. doi: 10.1016/s0887-8994(03)00211-x. PMID: 14580653. | No relevant outcomes reported (speech sound / intelligibility) |
| El-Mazahi MM, El-Mahdi MAEF, El-Khaleegy HAEA, Gomaah GHA. Cognitive and behavioural outcome of preterm versus full term infants, at school entry age. Trends in Medical Research. 2014;9(1):44-52. doi: 10.3923/tmr.2014.44.52 | No relevant outcomes reported (speech sound / intelligibility) |
| Engstrand O, Williams K, Lacerda F. Does babbling sound native? Listener responses to vocalizations produced by Swedish and American 12- and 18-month-olds. Phonetica. 2003 Jan-Mar;60(1):17-44. doi: 10.1159/000070452. PMID: 12802087. | Gestational age of participants not reported |
| Farquharson K, Hogan TP, Hoffman L, Wang J, Green KF, Green JR. A longitudinal study of infants' early speech production and later letter identification. PLoS One. 2018 Oct 10;13(10):e0204006. doi: 10.1371/journal.pone.0204006. PMID: 30304048; PMCID: PMC6179191. | No preterm participants |
| Fasolo M, Majorano M, D'Odorico L. Babbling and first words in children with slow expressive development. Clin Linguist Phon. 2008 Feb;22(2):83-94. doi: 10.1080/02699200701600015. PMID: 17896213. | Gestational age of participants not reported |
| Fernandes DMZ, Lima MCMP. Child development: Assessment of receptive and expressive language in preschoolers. Estudos de Psicologia. 2024;41:e210045-e210045. doi: 10.1590/1982-0275202441e210045 | Gestational age of participants not reported |
| Flabiano FC, Bühler KE, Limongi SC. Expressive language and cognitive development: diversity and complexity of children's productions. Pro Fono. 2010 Oct-Dec;22(4):525-30. English, Portuguese. doi: 10.1590/s0104-56872010000400028. PMID: 21271111. | Not available in English |
| Fort M, Lammertink I, Peperkamp S, Guevara-Rukoz A, Fikkert P, Tsuji S. Symbouki: a meta-analysis on the emergence of sound symbolism in early language acquisition. Dev Sci. 2018 Sep;21(5):e12659. doi: 10.1111/desc.12659. Epub 2018 Mar 15. PMID: 29542266. | Wrong publication type |
| Forrest, K., & Morrisette, M. L. Feature analysis of segmental errors in children with phonological disorders. Journal of speech, language, and hearing research : JSLHR, 1999; 42(1), 187-194. https://doi.org/doi:https://dx.doi.org/10.1044/jslhr.4201.187 | Gestational age of participants not reported |
| Forslund, M., & Bjerre, I. Follow-up of preterm children: II. Growth and development at four years of age. Early Human Development, 1990; 24(2), 107 EP - 118. | No relevant outcomes reported |
| Foster-Cohen S, Edgin JO, Champion PR, Woodward LJ. Early delayed language development in very preterm infants: evidence from the MacArthur-Bates CDI. J Child Lang. 2007 Aug;34(3):655-75. doi: 10.1017/s0305000907008070. PMID: 17822143. | No relevant outcomes reported (speech sound / intelligibility) |
| Fotuhi M, Yadegari F, Teymouri R. Vowels Development in Babbling of typically developing 6-to-12-month old Persian-learning Infants. Logoped Phoniatr Vocol. 2017 Oct;42(3):118-125. doi: 10.1080/14015439.2016.1221446. Epub 2016 Sep 6. PMID: 27597230. | Gestational age of participants not reported |
| Fritz J, Lamadrid-Figueroa H, Muñoz-Rocha TV, Huerta-García Y, Martínez-Silva G, Trejo-Valdivia B, Martínez-Medina S, Hernandez-Chavez C, Osorio-Valencia E, Burris HH, Peterson KE, Wright RO, Téllez-Rojo MM. Cesarean birth is associated with lower motor and language development scores during early childhood: a longitudinal analysis of two cohorts. Sci Rep. 2024 Oct 8;14(1):23438. doi: 10.1038/s41598-024-73914-7. PMID: 39379499; PMCID: PMC11461664. | No relevant outcomes reported (speech sound / intelligibility) |
| Gaines BR. Clinical focus: screening programs. Screening for childhood speech-language problems. Canadian Nurse. 2002;98(5):15-16. | No relevant outcomes reported (speech sound / intelligibility) |
| Gallagher, T. M., & Watkin, K. L. Prematurity and language developmental risk: too young or too small? Topics in Language Disorders, 1998; 18(3), 15-96. https://doi.org/doi:10.1097/00011363-199805000-00005 | Review/Discussion paper |
| Gerhold K, Torrington Eaton C, Newman RS, Bernstein Ratner N. Early Phonological Predictors of Toddler Language Outcomes. Folia Phoniatr Logop. 2020;72(6):442-453. doi: 10.1159/000503230. Epub 2019 Oct 22. PMID: 31639816. | Gestational age of participants not reported |
| Gibson V, Somogyi E, Nomikou I, Taylor D, López B, Mulenga IC, Davila-Ross M. Preverbal infants produce more protophones with artificial objects compared to natural objects. Sci Rep. 2023 Jun 20;13(1):9969. doi: 10.1038/s41598-023-36734-9. PMID: 37339994; PMCID: PMC10282070. | Gestational age of participants not reported |
| Gilbers D. Conflicting phonologically based and phonetically based constraints in the analysis of liquid-nasal substitutions. Clin Linguist Phon. 2001;15(1-2):23-8. doi: 10.3109/02699200109167625. PMID: 21269093. | Wrong publication type |
| Gildersleeve-Neumann CE, Davis BL, Macneilage PF. Syllabic patterns in the early vocalizations of Quichua children. *Applied Psycholinguistics*. 2013;34(1):111-134. doi:10.1017/S0142716411000634 | Gestational age of participants not reported |
| Gildersleeve-Neumann CE. Constraints on infant speech acquisition: A cross-language perspective. Dissertation Abstracts International: Section B: The Sciences and Engineering. 2002;62(11):5075. | Gestational age of participants not reported |
| Giulivi S, Whalen DH, Goldstein LM, Nam H, Levitt AG. An Articulatory Phonology Account of Preferred Consonant-Vowel Combinations. Lang Learn Dev. 2011;7(3):202-225. doi: 10.1080/15475441.2011.564569. Epub 2011 Jul 18. PMID: 23505343; PMCID: PMC3596049. | Gestational age of participants not reported |
| Glaspey AM, Wilson JJ, Reeder JD, Tseng WC, MacLeod AAN. Moving Beyond Single Word Acquisition of Speech Sounds to Connected Speech Development With Dynamic Assessment. J Speech Lang Hear Res. 2022 Feb 9;65(2):508-524. doi: 10.1044/2021_JSLHR-21-00188. Epub 2022 Jan 20. PMID: 35050702. | Gestational age of participants not reported |
| Goldstein B, Gildersleeve-Neumann C. Typical phonological acquisition in bilinguals. Perspectives on Language Learning & Education. 2007;14(2). doi: 10.1044/lle14.2.11 | Unable to source |
| Gonzalez LS, Robison D. A comparison of linguistic and behavioral performance of children born preterm and full term. Infant-Toddler Intervention. 2001;11(3):159-179. | Unable to source |
| Gordon S. As they grow: 1 year. Hey, can we talk? Parents. 2004;79(4):175-176. | Unable to source |
| Graves G. As they grow. 1 year: toddler talk. Parents.2003;78(4):163-164 | Unable to source |
| Grigos MI, Saxman JH, Gordon AM. Speech motor development during acquisition of the voicing contrast. J Speech Lang Hear Res. 2005 Aug;48(4):739-52. doi: 10.1044/1092-4388(2005/051). PMID: 16378470. | Gestational age of participants not reported |
| Grigos MI. Changes in articulator movement variability during phonemic development: a longitudinal study. J Speech Lang Hear Res. 2009 Feb;52(1):164-77. doi: 10.1044/1092-4388(2008/07-0220). Epub 2008 Jul 29. PMID: 18664683. | Unable to source |
| Grunau, R. V., Kearney, S. M., & Whitfield, M. F. Language development at 3 years in pre-term children of birth weight below 1000 g. The British journal of disorders of communication, 1990; 25(2), 173-182. https://doi.org/doi:https://dx.doi.org/10.3109/13682829009011972 | No relevant outcomes reported |
| Guarini A, Sansavini A, Fabbri C, Alessandroni R, Faldella G, Karmiloff-Smith A. Reconsidering the impact of preterm birth on language outcome. Early Hum Dev. 2009 Oct;85(10):639-45. doi: 10.1016/j.earlhumdev.2009.08.061. Epub 2009 Sep 11. PMID: 19748193. | No relevant outcomes reported (speech sound / intelligibility) |
| Guarini A., Sansavini A. (2012). Language, executive functions, short-term memory and literacy in preterm children: A longitudinal study. RIVISTA DI PSICOLINGUISTICA APPLICATA, XII(3), 101-115. | Unable to source |
| Guedes DZ, Primi R, Kopelman BI. Bayley infant neurodevelopmental screener (bins) in brazilian preterm children under risk conditions. Archives of Disease in Childhood. 2012;97:A356. doi: 10.1136/archdischild-2012-302724.1247 | No relevant outcomes reported (speech sound / intelligibility) |
| Haapsamo H, Kuusikko-Gauffin S, Ebeling H, Larinen K, Penninkilampi-Kerola V, Soini H, Moilanen I. (2012). Communication development and characteristics of influencing factors: a follow-up study from 8 to 36 months. *Early Child Development and Care*, *183*(2), 321–334. https://doi.org/10.1080/03004430.2012.674523 | Gestational age of participants not reported |
| Hambly H, Wren Y, McLeod S, Roulstone S. The influence of bilingualism on speech production: a systematic review. Int J Lang Commun Disord. 2013 Jan;48(1):1-24. doi: 10.1111/j.1460-6984.2012.00178.x. Epub 2012 Jul 30. PMID: 23317381. | Wrong publication type |
| Han MJ, Kim SJ. Characteristics of Functional Speech Sound Disorders in Korean Children. Annals of Child Neurology. 2022;30(1):8-16. doi: 10.26815/acn.2021.00535 | Outcome not explored in relation to GA |
| Harold MP, Barlow SM. Effects of environmental stimulation on infant vocalizations and orofacial dynamics at the onset of canonical babbling. Infant Behav Dev. 2013 Feb;36(1):84-93. doi: 10.1016/j.infbeh.2012.10.001. Epub 2012 Dec 20. PMID: 23261792; PMCID: PMC3580028. | No preterm participants |
| Hassanzadeh Rad A, Hassanzadeh Rad Z. The relationship between preterm birth and language impairment in children. Iranian Journal of Reproductive Medicine. 2013;11:102-103. | Wrong publication type |
| Hesketh E, White P, Simkiss D, Roulstone S. Outcomes from a community speech and language therapy service treatment waiting list: The natural history of 525 children with identified speech and language needs. Int J Lang Commun Disord. 2023 Sep-Oct;58(5):1510-1525. doi: 10.1111/1460-6984.12877. Epub 2023 May 15. PMID: 37189292. | Gestational age of participants not reported |
| Highman C, Hennessey NW, Leitão S, Piek JP. Early development in infants at risk of childhood apraxia of speech: a longitudinal investigation. Dev Neuropsychol. 2013;38(3):197-210. doi: 10.1080/87565641.2013.774405. PMID: 23573797. | Gestational age of participants not reported |
| Hille, E. T., den Ouden, A. L., Bauer, L., van den Oudenrijn, C., Brand, R., & Verloove-Vanhorick, S. P. School performance at nine years of age in very premature and very low birth weight infants: perinatal risk factors and predictors at five years of age. Collaborative Project on Preterm and Small for Gestational Age (POPS) Infants in The Netherlands. The Journal of pediatrics, 1994; 125(3), 426-434. https://doi.org/doi:https://dx.doi.org/10.1016/s0022-3476(05)83290-1 | No relevant outcomes reported |
| Holdgrafer, G. Relationships between measures of phonological performance in preterm children. Perceptual and motor skills, 1995; 81(1), 41 EP - 42. https://doi.org/doi:http://dx.doi.org/10.2466/pms.1995.81.1.41 | Conference abstract only |
| Hwa-Froelich DA, Matsuo H. Communication development and differences in children adopted from China and Eastern Europe. Lang Speech Hear Serv Sch. 2010 Jul;41(3):349-66. doi: 10.1044/0161-1461(2009/08-0085). Epub 2009 Aug 24. PMID: 19704113. | Gestational age of participants not reported |
| Iloeje, S. O., & Izuora, G. I. The pattern and prognosis of speech disorders among children in Enugu, Nigeria. Annals of tropical paediatrics, 1991; 11(1), 25-31. https://doi.org/doi:https://dx.doi.org/10.1080/02724936.1991.11747474 | Gestational age of participants not reported |
| Islam UA, Poole KL, Schmidt LA, Ford J, Saigal S, Van Lieshout RJ. Childhood language skills and adolescent self-esteem in preterm survivors. J Child Health Care. 2018 Mar;22(1):34-45. doi: 10.1177/1367493517739158. Epub 2017 Nov 6. PMID: 29110533. | No relevant outcomes reported (speech sound / intelligibility) |
| Iyer SN, Ertmer DJ. Relationships between vocalization forms and functions in infancy: preliminary implications for early communicative assessment and intervention. Am J Speech Lang Pathol. 2014 Nov;23(4):587-98. doi: 10.1044/2014_AJSLP-13-0091. PMID: 25029461. | Gestational age of participants not reported |
| Jeba A, Suganthirababu P, Sosale S, Alagesan J, Srinivasan V, Sebastian N. Neurodevelopmental Outcome Of Very Preterm And Moderate To Late Preterm Babies At The Corrected Age Of First Year. European Journal of Molecular and Clinical Medicine. 2022;9(8):280-290. | No relevant outcomes reported (speech sound / intelligibility) |
| Jenkins C, Fineran KR, Lange A. Birth and infancy: Physical and cognitive development. Human growth and development across the lifespan: Applications for counselors. 2016: 113-149. | No preterm participants |
| Jennische, M., Sedin, G., Johnsen, B., & Sundelin, C. Assessment of speech and language skills in children. Upsala journal of medical sciences, 1992; 97(3), 229 EP - 250. | Gestational age of participants not reported |
| Jensen, T. S., Bøggild-Andersen, B., Schmidt, J., Ankerhus, J., & Hansen, E. Perinatal risk factors and first-year vocalizations: Influence on preschool language and motor performance. Developmental Medicine & Child Neurology, 1988; 30(2), 153-161. https://doi.org/doi:https://doi.org/10.1111/j.1469-8749.1988.tb04746.x | Gestational age of participants not reported |
| Jhang Y, Oller DK. Emergence of Functional Flexibility in Infant Vocalizations of the First 3 Months. Front Psychol. 2017 Mar 24;8:300. doi: 10.3389/fpsyg.2017.00300. PMID: 28392770; PMCID: PMC5364184. | Gestational age of participants not reported |
| Johnstone A. The developmental outcomes of late preterm infants. Dissertation Abstracts International: Section B: The Sciences and Engineering. 2013;74(4). | Wrong publication type |
| Jois RS. Neurodevelopmental outcome of late-preterm infants: A pragmatic review. Australian Journal of General Practice. 2018;47(11):776-785. doi: 10.31128/AJGP-03-18-4539 | Wrong publication type |
| Karousou A, López-Ornat S. Prespeech vocalizations and the emergence of speech: a study of 1005 Spanish children. Span J Psychol. 2013;16:E32. doi: 10.1017/sjp.2013.27. PMID: 23866227. | No preterm participants |
| Keating D, Turrell G, Ozanne A. Childhood speech disorders: reported prevalence, comorbidity and socioeconomic profile. J Paediatr Child Health. 2001 Oct;37(5):431-6. doi: 10.1046/j.1440-1754.2001.00697.x. PMID: 11885704. | Gestational age of participants not reported |
| Kehoe M. Relationships between lexical and phonological development: a look at bilingual children--a commentary on Stoel-Gammon's 'Relationships between lexical and phonological development in young children'. J Child Lang. 2011 Jan;38(1):75-81. doi: 10.1017/S0305000910000474. Epub 2010 Oct 18. PMID: 20950499. | Wrong publication type |
| Kehoe MM, Lleó C. Intervocalic consonants in the acquisition of German: onsets, codas or something else? Clin Linguist Phon. 2002 Apr-May;16(3):169-82. doi: 10.1080/02699200110112213. PMID: 12064026. | Gestational age of participants not reported |
| Kehoe MM, Lleó C. The acquisition of nuclei: a longitudinal analysis of phonological vowel length in three German-speaking children. J Child Lang. 2003 Aug;30(3):527-56. doi: 10.1017/s030500090300566x. PMID: 14513467. | Gestational age of participants not reported |
| Kehoe MM, Stoel-Gammon C. Development of syllable structure in English-speaking children with particular reference to rhymes. J Child Lang. 2001 Jun;28(2):393-432. doi: 10.1017/s030500090100469x. PMID: 11449945. | Gestational age of participants not reported |
| Keren-Portnoy T, Majorano M, Vihman MM. From phonetics to phonology: the emergence of first words in Italian. J Child Lang. 2009 Mar;36(2):235-67. doi: 10.1017/S0305000908008933. Epub 2008 Sep 15. PMID: 18789180. | Gestational age of participants not reported |
| Kim SH, Jung E, Lee HN, Lee JM, Park SH, Jeong J, Lee BS, Kim EA, Kim KS. Outcomes at 18-24 Months of Infants with Birth Weight under 500 g Born in Korea during 2013-2017: A Nationwide Cohort Study. Neonatology. 2024;121(1):74-80. doi: 10.1159/000534194. Epub 2023 Oct 20. PMID: 37866355. | No relevant outcomes reported (speech sound / intelligibility) |
| Kim SW, Youk T, Kim J. Maternal and Neonatal Risk Factors Affecting the Occurrence of Neurodevelopmental Disorders: A Population-Based Nationwide Study. Asia Pac J Public Health. 2022 Mar;34(2-3):199-205. doi: 10.1177/10105395211066383. Epub 2021 Dec 21. PMID: 34933590. | Gestational age of participants not reported |
| Klein HB. Reduplication revisited: functions, constraints, repairs, and clinical implications. Am J Speech Lang Pathol. 2005 Feb;14(1):71-83. doi: 10.1044/1058-0360(2005/009). PMID: 15962848. | Gestational age of participants not reported |
| Koc OO, Kavuncuoglu S, Aslan G, Koprulu A. Evaluation of neurodevelopmental condition of very low birth weight prematures during school age. Early Human Development. 2010;86:S142. doi: 10.1016/j.earlhumdev.2010.09.376 | Wrong publication type |
| Krägeloh-Mann I. The cognitive outcome of very preterm children: how to interpret results. Acta Paediatr. 2002;91(12):1285-7. doi: 10.1111/j.1651-2227.2002.tb02818.x. PMID: 12578279. | Wrong publication type |
| Kristoffersen KE, Simonsen HG. (2006) ‘The acquisition of #sC clusters in Norwegian’, *Journal of Multilingual Communication Disorders*, 4(3), pp. 231–241. doi: 10.1080/14769670601110556. | Gestational age of participants not reported |
| Krogh MT, Vaever MS. Bayley-III: Cultural differences and language scale validity in a Danish sample. Scand J Psychol. 2016 Dec;57(6):501-508. doi: 10.1111/sjop.12333. Epub 2016 Sep 26. PMID: 27716932. | No preterm participants |
| Kulikov, G. A., Andreeva, N. G., Lyakso, E. E., & Pavlikova, M. I. Speech-like elements in the sounds produced by children in the first months of life. Sensory Systems, 1999; 13(1), 61-70. | Unable to source article |
| Laing C, Bergelson E. From babble to words: Infants' early productions match words and objects in their environment. Cogn Psychol. 2020 Nov;122:101308. doi: 10.1016/j.cogpsych.2020.101308. Epub 2020 Jun 3. PMID: 32504852; PMCID: PMC7572567. | No preterm participants |
| Laing CE. (2019). Phonological Motivation for the Acquisition of Onomatopoeia: An Analysis of Early Words. *Language Learning and Development*, *15*(2), 177–197. https://doi.org/10.1080/15475441.2019.1577138 | Gestational age of participants not reported |
| Lamônica DAC, Becaro CK, Borba AC, Maximino LP, Costa ARAD, Ribeiro CDC. Communicative performance and vocabulary domain in preschool preterm infants. J Appl Oral Sci. 2018 Jul 16;26:e20170186. doi: 10.1590/1678-7757-2017-0186. PMID: 30020349; PMCID: PMC6089568. | No relevant outcomes reported (speech sound / intelligibility) |
| Lang S, Willmes K, Marschik PB, Zhang D, Fox-Boyer A. Prelexical phonetic and early lexical development in German-acquiring infants: canonical babbling and first spoken words. Clin Linguist Phon. 2021 Feb 1;35(2):185-200. doi: 10.1080/02699206.2020.1731606. Epub 2020 Mar 3. PMID: 32126852. | Gestational age of participants not reported |
| Lee CC, Jhang Y, Relyea G, Chen LM, Oller DK. Babbling development as seen in canonical babbling ratios: A naturalistic evaluation of all-day recordings. Infant Behav Dev. 2018 Feb;50:140-153. doi: 10.1016/j.infbeh.2017.12.002. Epub 2017 Dec 29. PMID: 29289753; PMCID: PMC5869132. | Gestational age of participants not reported |
| Lee CC. Vocal development in English- and Chinese-learning infants. Dissertation Abstracts International Section A: Humanities and Social Sciences. 2018;78(12) | Gestational age of participants not reported |
| Levitt, A. G., & Utman, J. A. From babbling towards the sound systems of English and French: A longitudinal two-case study. Journal of Child Language, 1992; 19(1), 19-49. https://doi.org/doi:https://doi.org/10.1017/S0305000900013611 | Gestational age of participants not reported |
| Li Y. Differential acquisition of phonemic contrasts by infant word-learners: Does production recapitulate perception? Dissertation Abstracts International Section A: Humanities and Social Sciences. 2007;68(6):2431. | Gestational age of participants not reported |
| Lian WB, Ho SK, Choo SH, Shah VA, Chan DK, Yeo CL, Ho LY. Children with developmental and behavioural concerns in Singapore. Singapore Medical Journal. 2012;53(7):439-445. | Outcome not explored in relation to GA |
| Lieberman M, Sand A, Lohmander A, Miniscalco C. Asking parents about babbling at 10 months produced valid answers but did not predict language screening result 2 years later. Acta Paediatr. 2022 Oct;111(10):1914-1920. doi: 10.1111/apa.16486. Epub 2022 Jul 28. PMID: 35837842; PMCID: PMC9541482. | Gestational age of participants not reported |
| Locke JL. Movement patterns in spoken language. Science. 2000 Apr 21;288(5465):449-51. doi: 10.1126/science.288.5465.449. PMID: 10798981. | Wrong publication type |
| Logrieco MG, Nicolì I, Spinelli M, Lionetti F, D'Urso G, Guerra GC, D'Aloia V, Toto G, Fasolo M. Early, typical, and late talkers: an exploratory study on predictors of language development in the first two years of life. F1000Res. 2024 Jul 16;13:798. doi: 10.12688/f1000research.145763.1. PMID: 39139467; PMCID: PMC11320034. | Gestational age of participants not reported |
| Long HL, Ramsay G, Griebel U, Bene ER, Bowman DD, Burkhardt-Reed MM, Oller DK. Canonical babbling during vocal turn taking and independent vocal play. bioRxiv. 2020. doi: 10.1101/2020.10.09.333872 | Gestational age of participants not reported |
| Long HL, Ramsay G, Griebel U, Bene ER, Bowman DD, Burkhardt-Reed MM, Oller DK. Perspectives on the origin of language: Infants vocalize most during independent vocal play but produce their most speech-like vocalizations during turn taking. PLoS One. 2022 Dec 30;17(12):e0279395. doi: 10.1371/journal.pone.0279395. PMID: 36584126; PMCID: PMC9803194. | Gestational age of participants not reported |
| Lowe JR, Nolen TL, Vohr B, Adams-Chapman I, Duncan AF, Watterberg K. Effect of primary language on developmental testing in children born extremely preterm. Acta Paediatr. 2013 Sep;102(9):896-900. doi: 10.1111/apa.12310. Epub 2013 Jun 19. PMID: 23735043; PMCID: PMC4108617. | No relevant outcomes reported (speech sound / intelligibility) |
| Luinge MR, Post WJ, Wit HP, Goorhuis-Brouwer SM. The ordering of milestones in language development for children from 1 to 6 years of age. J Speech Lang Hear Res. 2006 Oct;49(5):923-40. doi: 10.1044/1092-4388(2006/067). PMID: 17077206. | Gestational age of participants not reported |
| Lyakso EE, Frolova OV, Grigorev AS. (2014). Infant Vocalizations at the First Year of Life Predict Speech Development at 2 - 7 Years: Longitudinal Study. Psychology, 5, 1433-1445. http://dx.doi.org/10.4236/psych.2014.512154 | No preterm participants |
| Lyakso EE, Grigor’ev AS. Dynamics of the Duration and Frequency Characteristics of Vowels during the First Seven Years of Life in Children. *Neurosci Behav Physi* **45**, 558–567 (2015). https://doi.org/10.1007/s11055-015-0110-z | Gestational age of participants not reported |
| Määttä S, Laakso ML, Ahonen T, Tolvanen A, Westerholm J, Aro T. Continuity From Prelinguistic Communication to Later Language Ability: A Follow-Up Study From Infancy to Early School Age. J Speech Lang Hear Res. 2016 Dec 1;59(6):1357-1372. doi: 10.1044/2016_JSLHR-L-15-0209. Erratum in: J Speech Lang Hear Res. 2017 Jun 10;60(6):1661. doi: 10.1044/2017_JSLHR-L-17-0156. PMID: 27788278. | Gestational age of participants not reported |
| Määttä S, Laakso ML, Tolvanen A, Ahonen T, Aro T. Children with differing developmental trajectories of prelinguistic communication skills: language and working memory at age 5. J Speech Lang Hear Res. 2014 Jun 1;57(3):1026-39. doi: 10.1044/2014_JSLHR-L-13-0012. PMID: 24686441. | Gestational age of participants not reported |
| Määttä S, Laakso ML, Tolvanen A, Ahonen T, Aro T. Developmental trajectories of early communication skills. J Speech Lang Hear Res. 2012 Aug;55(4):1083-96. doi: 10.1044/1092-4388(2011/10-0305). Epub 2012 Jan 9. PMID: 22232414. | Gestational age of participants not reported |
| MacLeod AA, Sutton A, Trudeau N, Thordardottir E. The acquisition of consonants in Québécois French: a cross-sectional study of pre-school aged children. Int J Speech Lang Pathol. 2011 Apr;13(2):93-109. doi: 10.3109/17549507.2011.487543. Epub 2010 Sep 7. PMID: 20822378. | Gestational age of participants not reported |
| MacNeilage PF, Davis BL. Baby talk and the emergence of first words. *Behavioral and Brain Sciences*. 2004;27(4):517-518. doi:10.1017/S0140525X04380118 | Wrong publication type |
| MacNeilage, P. F., Davis, B. L., & Matyear, C. L. Babbling and first words: Phonetic similarities and differences. Speech Communication, 1997; 22(2), 269-277. https://doi.org/doi:https://doi.org/10.1016/S0167-6393(97)00022-8 | Gestational age of participants not reported |
| Macrae T. Lexical and child-related factors in word variability and accuracy in infants. Clin Linguist Phon. 2013 Jul;27(6-7):497-507. doi: 10.3109/02699206.2012.752867. Epub 2013 Jan 22. Erratum in: Clin Linguist Phon. 2017;31(3):250. doi: 10.1080/02699206.2017.1289330. PMID: 23339451. | Gestational age of participants not reported |
| Maekawa J, Storkel HL. Individual differences in the influence of phonological characteristics on expressive vocabulary development by young children. J Child Lang. 2006 Aug;33(3):439-59. doi: 10.1017/s0305000906007458. PMID: 17017275; PMCID: PMC1618766. | Gestational age of participants not reported |
| Magill-Evans J, Harrison MJ, Van der Zalm J, Holdgrafer G. Cognitive and language development of healthy preterm infants at 10 years of age. Phys Occup Ther Pediatr. 2002;22(1):41-56. PMID: 12174504. | No relevant outcomes reported (speech sound / intelligibility) |
| Mahurin-Smith J, DeThorne LS, Petrill SA. Children Born Prematurely May Demonstrate Catch-Up Growth in Pre-Adolescence. Lang Speech Hear Serv Sch. 2021 Apr 20;52(2):675-685. doi: 10.1044/2020_LSHSS-20-00111. Epub 2021 Mar 3. PMID: 33656921; PMCID: PMC8711712. | No relevant outcomes reported (speech sound / intelligibility) |
| Mariam Korah R, Paul S, Merry ER, Seby MM, Manjula R, Krishnan G. Effect of neighborhood density on speech sound acquisition in typically developing malayalam speaking children aged 1-3 years. Journal of the All India Institute of Speech & Hearing. 2011;30:23-32. | Gestational age of participants not reported |
| Maria-Mengel MR, Martins Linhares MB. Risk factors for infant developmental problems. Rev Lat Am Enfermagem. 2007 Sep-Oct;15 Spec No:837-42. doi: 10.1590/s0104-11692007000700019. PMID: 17934592. | Gestational age of participants not reported |
| Marlow, N., D'Souza, S. W., & Chiswick, M. L. Neurodevelopmental outcome in babies weighing less than 2001 g at birth. British Medical Journal, 1987; 294(6587), 1582 EP - 1586. | No relevant outcomes reported |
| Martin A, Utsugi A, Mazuka R. The multidimensional nature of hyperspeech: evidence from Japanese vowel devoicing. Cognition. 2014 Aug;132(2):216-28. doi: 10.1016/j.cognition.2014.04.003. Epub 2014 May 9. PMID: 24813573. | No preterm participants |
| Martins AC, Nascimento D, Pinto S, Alves D, Wertzner HF, Castro A. Phonological characteristics of European and Brazilian Portuguese in children with Speech Sound Disorders. Clin Linguist Phon. 2021 Nov 2;35(11):1076-1090. doi: 10.1080/02699206.2020.1866673. Epub 2021 Jan 17. PMID: 33459062. | Gestational age of participants not reported |
| Másdóttir T, Stokes SF. Influence of consonant frequency on Icelandic-speaking children's speech acquisition. Int J Speech Lang Pathol. 2016 Apr;18(2):111-21. doi: 10.3109/17549507.2015.1060525. Epub 2015 Jul 29. PMID: 26220368. | Gestational age of participants not reported |
| McAllister, L., Masel, C., Tudehope, D., O'Callaghan, M., Mohay, H., & Rogers, Y. Speech and language outcomes in preschool-aged survivors of neonatal intensive care. European Journal of Disorders of Communication, 1993; 28(4), 383-394. https://doi.org/doi:10.3109/13682829309041472 | Not possible to extract data for PT |
| McCathren, R. B., Yoder, P. J., & Warren, S. F. The relationship between prelinguistic vocalization and later expressive vocabulary in young children with developmental delay. Journal of speech, language, and hearing research : JSLHR, 1999; 42(4), 915-924. https://doi.org/doi:https://dx.doi.org/10.1044/jslhr.4204.915 | 4/58 participants pre-term and unable to differentiate in analysis |
| McConkey R, Swift A, Titterington J. Changes in Children's Speech and Language Difficulties from Age Five to Nine: An Irish National, Longitudinal Study. Int J Environ Res Public Health. 2021 Aug 11;18(16):8483. doi: 10.3390/ijerph18168483. PMID: 34444228; PMCID: PMC8392088. | Outcome not explored in relation to GA |
| McCune L, Vihman MM. Early phonetic and lexical development: a productivity approach. J Speech Lang Hear Res. 2001 Jun;44(3):670-84. doi: 10.1044/1092-4388(2001/054). PMID: 11407570. | Gestational age of participants not reported |
| McGowan JE, Alderdice FA, Holmes VA, Johnston L. Early childhood development of late-preterm infants: a systematic review. Pediatrics. 2011 Jun;127(6):1111-24. doi: 10.1542/peds.2010-2257. Epub 2011 May 29. PMID: 21624885. | Wrong publication type |
| McGowan RW, McGowan RS, Denny M, Nittrouer S. A longitudinal study of very young children's vowel production. Journal of speech, language, and hearing research : JSLHR. 2014;57(1):1-15. doi: 10.1044/1092-4388%282013/12-0112%29 | Gestational age of participants not reported |
| McGowan S. Neurodevelopmental Outcomes Associated with Prematurity from Infancy Through Early School Age: A Literature Review. Pediatric Nursing. 2022;48(5):223-247. doi: 10.62116/pnj.2022.48.5.223 | Wrong publication type |
| McGrath JM. How does prematurity affect the development of speech and language? Communicating Nursing Research. 2005;38:341-341. | Wrong publication type |
| McKean C, Reilly S, Bavin EL, Bretherton L, Cini E, Conway L, Cook F, Eadie P, Prior M, Wake M, Mensah F. Language Outcomes at 7 Years: Early Predictors and Co-Occurring Difficulties. Pediatrics. 2017 Mar;139(3):e20161684. doi: 10.1542/peds.2016-1684. Epub 2017 Feb 8. PMID: 28179482. | Gestational age of participants not reported |
| McLeod S, Crowe K. Children's Consonant Acquisition in 27 Languages: A Cross-Linguistic Review. Am J Speech Lang Pathol. 2018 Nov 21;27(4):1546-1571. doi: 10.1044/2018_AJSLP-17-0100. PMID: 30177993. | Gestational age of participants not reported |
| McLeod S, van Doorn J, Reed VA. Normal acquisition of consonant clusters. American Journal of Speech-Language Pathology.2001;10(2):99-110. doi: 10.1044/1058-0360(2001/011) | Wrong publication type |
| Mealings KT, Cox F, Demuth K. Acoustic investigations into the later acquisition of syllabic -es plurals. J Speech Lang Hear Res. 2013 Aug;56(4):1260-71. doi: 10.1044/1092-4388(2012/12-0163). Epub 2013 Jun 19. PMID: 23785190. | Gestational age of participants not reported |
| Ménard L, Schwartz JL, Boë LJ. Role of vocal tract morphology in speech development: perceptual targets and sensorimotor maps for synthesized French vowels from birth to adulthood. J Speech Lang Hear Res. 2004 Oct;47(5):1059-80. doi: 10.1044/1092-4388(2004/079). PMID: 15603462. | No relevant outcomes reported (speech sound / intelligibility) |
| Milligan DW. Outcomes of children born very preterm in Europe. Arch Dis Child Fetal Neonatal Ed. 2010 Jul;95(4):F234-40. doi: 10.1136/adc.2008.143685. PMID: 20576663. | Wrong publication type |
| Mitchell, P. R., & Kent, R. D. Phonetic variation in multisyllable babbling. Journal of Child Language, 1990; 17(2), 247 EP - 265. | Gestational age of participants not reported |
| Molad M, Gover A, Marai Z, Lavie-Nevo K, Kessel I, Shemer-Meiri L, Soloveichik M. Neurodevelopmental Outcome of Very Low Birth Weight Infants in the Northern District of Israel: A Cross-Sectional Study. Children (Basel). 2023 Jul 31;10(8):1320. doi: 10.3390/children10081320. PMID: 37628319; PMCID: PMC10453082. | No relevant outcomes reported (speech sound / intelligibility) |
| Moore, C. A., & Ruark, J. L. Does speech emerge from earlier appearing oral motor behaviors? Journal of Speech & Hearing Research, 1996; 39(5), 1034-1047. https://doi.org/doi:10.1044/jshr.3905.1034 | Gestational age of participants not reported |
| Morgan L, Wren YE. A Systematic Review of the Literature on Early Vocalizations and Babbling Patterns in Young Children. Communication Disorders Quarterly. 2018;40(1). doi: 10.1177/1525740118760215 | Wrong publication type |
| Morris SR. Clinical application of the mean babbling level and syllable structure level. Lang Speech Hear Serv Sch. 2010 Apr;41(2):223-30. doi: 10.1044/0161-1461(2009/08-0076). Epub 2009 Sep 15. PMID: 19755639. | No relevant outcomes reported (speech sound / intelligibility) |
| Mount, R., Reznick, J. S., Kagan, J., Hiatt, S., & Szpak, M. Direction of gaze and emergence of speech in the second year. Brain and language, 1989; 36(3), 406-410. https://doi.org/doi:https://dx.doi.org/10.1016/0093-934x(89)90076-x | Gestational age of participants not reported |
| Mukhopadhyay K, Malhi P, Kumar J, Singhi P. Cognitive, Language, and Visuomotor Abilities of Very Low Birthweight Infants at Corrected Age of Two Years. Indian Pediatr. 2020 Apr 15;57(4):296-300. Epub 2020 Feb 5. PMID: 32038037. | No relevant outcomes reported (speech sound / intelligibility) |
| Mulderry C, Jackson BN, Purdy SC. Children's speech, language and communication skills and parental knowledge in the growing up in New Zealand cohort. Int J Lang Commun Disord. 2024 Sep-Oct;59(5):1850-1864. doi: 10.1111/1460-6984.13035. Epub 2024 Apr 11. PMID: 38602295. | Gestational age of participants not reported |
| Murillo E, Capilla A. Properties of vocalization- and gesture-combinations in the transition to first words. J Child Lang. 2016 Jul;43(4):890-913. doi: 10.1017/S0305000915000343. Epub 2015 Jul 24. PMID: 26204930. | No preterm participants |
| Nagy BE, Kenyhercz F. Adaptive Behavioral, Social-Emotional, and Neurodevelopmental Outcomes at 2 Years of Age in Hungarian Preterm Infants Based on Bayley III. Dev Neurorehabil. 2021 Jan;24(1):18-24. doi: 10.1080/17518423.2020.1764651. Epub 2020 May 18. PMID: 32420782. | No relevant outcomes reported (speech sound / intelligibility) |
| Nathani S, Ertmer DJ, Stark RE. Assessing vocal development in infants and toddlers. Clin Linguist Phon. 2006 Jul;20(5):351-69. doi: 10.1080/02699200500211451. PMID: 16728333; PMCID: PMC3412408. | Gestational age of participants not reported |
| Nelson HD, Nygren P, Walker M, Panoscha R. Screening for speech and language delay in preschool children: systematic evidence review for the US Preventive Services Task Force. Pediatrics. 2006 Feb;117(2):e298-319. doi: 10.1542/peds.2005-1467. Erratum in: Pediatrics. 2006 Jun;117(6):2336-7. PMID: 16452337. | Wrong publication type |
| Newton C, Wells B. Between-word junctures in early multi-word speech. J Child Lang. 2002 May;29(2):275-99. doi: 10.1017/s0305000902005044. PMID: 12109372. | Gestational age of participants not reported |
| Nguyen TN, Spencer-Smith M, Zannino D, Burnett A, Scratch SE, Pascoe L, Ellis R, Cheong J, Thompson D, Inder T, Doyle LW, Anderson PJ. Developmental Trajectory of Language From 2 to 13 Years in Children Born Very Preterm. Pediatrics. 2018 May;141(5):e20172831. doi: 10.1542/peds.2017-2831. Epub 2018 Apr 9. PMID: 29632253; PMCID: PMC5914491. | No relevant outcomes reported (speech sound / intelligibility) |
| Nikoghosyan KV, Tovmasyan IT, Mazmanyan PA. Neurodevelopmental outcome of very low birth weight infants at 2 years of corrected age. New Armenian Medical Journal. 2015;9(4):61-68 | No relevant outcomes reported (speech sound / intelligibility) |
| Nip IS, Green JR, Marx DB. Early speech motor development: Cognitive and linguistic considerations. J Commun Disord. 2009 Jul-Aug;42(4):286-98. doi: 10.1016/j.jcomdis.2009.03.008. Epub 2009 Apr 7. PMID: 19439318; PMCID: PMC2892123. | No preterm participants |
| Nip IS, Green JR, Marx DB. The co-emergence of cognition, language, and speech motor control in early development: a longitudinal correlation study. J Commun Disord. 2011 Mar-Apr;44(2):149-60. doi: 10.1016/j.jcomdis.2010.08.002. Epub 2010 Sep 17. Erratum in: J Commun Disord. 2012 Nov;45(6):e1. PMID: 21035125; PMCID: PMC3057350. | Gestational age of participants not reported |
| Nip ISB. Early speech motor development: The influences of cognitive and linguistic factors. Dissertation Abstracts International: Section B: The Sciences and Engineering. 2008;68(11):7297. | Wrong publication type |
| Nixon JS, Tomaschek F. Prediction and error in early infant speech learning: A speech acquisition model. Cognition. 2021 Jul;212:104697. doi: 10.1016/j.cognition.2021.104697. Epub 2021 Mar 31. PMID: 33798952; PMCID: PMC8173624. | No relevant outcomes reported (speech sound / intelligibility) |
| Nosarti C, Rushe TM, Woodruff PW, Stewart AL, Rifkin L, Murray RM. Corpus callosum size and very preterm birth: relationship to neuropsychological outcome. Brain. 2004 Sep;127(Pt 9):2080-9. doi: 10.1093/brain/awh230. Epub 2004 Aug 2. PMID: 15289268. | No relevant outcomes reported (speech sound / intelligibility) |
| Ogneva A, Pérez-Pereira M. Communicative and linguistic factors influencing language development at 30 months of age in preterm and full-term children: a longitudinal study using the CDI. Front Psychol. 2023 Jul 24;14:1177161. doi: 10.3389/fpsyg.2023.1177161. PMID: 37554136; PMCID: PMC10406507. | No relevant outcomes reported (speech sound / intelligibility) |
| Oller, D. K., Eilers, R. E., Neal, A. R., & Cobo-Lewis, A. B. Late onset canonical babbling: A possible early marker for abnormal development. American Journal on Mental Retardation, 1998; 103(3), 249-263. https://doi.org/doi:https://doi.org/10.1352/0895-8017(1998)103<0249:LOCBAP>2.0.CO;2 | 22/56 participants pre-term and unable to differentiate in analysis |
| Oller DK, Griebel U, Bowman DD, Bene E, Long HL, Yoo H, Ramsay G. Infant boys are more vocal than infant girls. Curr Biol. 2020 May 18;30(10):R426-R427. doi: 10.1016/j.cub.2020.03.049. PMID: 32428468; PMCID: PMC8204662. | Gestational age of participants not reported |
| Oller DK, Ramsay G, Bene E, Long HL, Griebel U. Protophones, the precursors to speech, dominate the human infant vocal landscape. Philos Trans R Soc Lond B Biol Sci. 2021 Oct 25;376(1836):20200255. doi: 10.1098/rstb.2020.0255. Epub 2021 Sep 6. PMID: 34482735; PMCID: PMC8419580. | Gestational age of participants not reported |
| Oohashi H, Watanabe H, Taga G. Acquisition of vowel articulation in childhood investigated by acoustic-to-articulatory inversion. Infant Behav Dev. 2017 Feb;46:178-193. doi: 10.1016/j.infbeh.2017.01.007. Epub 2017 Feb 20. PMID: 28222332. | Outcome not explored in relation to GA |
| Oohashi H, Watanabe H, Taga G. Acquisition of vowel articulation in childhood investigated by acoustic-to-articulatory inversion. Infant Behav Dev. 2017 Feb;46:178-193. doi: 10.1016/j.infbeh.2017.01.007. Epub 2017 Feb 20. PMID: 28222332. | Outcome not explored in relation to GA |
| Orr E. (2022). The nature of infant vocal behaviour development between 8 and 17 months and its role in communication with mother. *Educational and Developmental Psychologist*, *39*(2), 219–230. https://doi.org/10.1080/20590776.2022.2079408 | Gestational age of participants not reported |
| Overby M, Belardi K, Schreiber J. A retrospective video analysis of canonical babbling and volubility in infants later diagnosed with childhood apraxia of speech. Clin Linguist Phon. 2020 Jul 2;34(7):634-651. doi: 10.1080/02699206.2019.1683231. Epub 2019 Oct 29. PMID: 31661338. | Gestational age of participants not reported |
| Park JY, Jung NH. Developmental Characteristics of High-Risk Infants According to the Bayley Scales of Infant and Toddler Development-Third Edition. Child Care Health Dev. 2025 Jan;51(1):e70018. doi: 10.1111/cch.70018. PMID: 39629855. | No relevant outcomes reported (speech sound / intelligibility) |
| Pascal A, Naulaers G, Ortibus E, Oostra A, De Coen K, Michel S, Cloet E, Casaer A, D'haese J, Laroche S, Jonckheere A, Plaskie K, Van Mol C, Delanghe G, Bruneel E, Van Hoestenberghe MR, Samijn B, Govaert P, Van den Broeck C. Neurodevelopmental outcomes of very preterm and very-low-birthweight infants in a population-based clinical cohort with a definite perinatal treatment policy. Eur J Paediatr Neurol. 2020 Sep;28:133-141. doi: 10.1016/j.ejpn.2020.06.007. Epub 2020 Jul 16. PMID: 32788055. | No relevant outcomes reported (speech sound / intelligibility) |
| Polka L, Bohn OS. Natural referent vowel (NRV) framework: An emerging view of early phonetic development. Journal of Phonetics. 2011;39(4):467-478. doi: 10.1016/j.wocn.2010.08.007 | No relevant outcomes reported (speech sound / intelligibility) |
| Pollock KE, Berni MC. Incidence of non-rhotic vowel errors in children: data from the Memphis Vowel Project. Clin Linguist Phon. 2003 Jun-Aug;17(4-5):393-401. doi: 10.1080/0269920031000079949. PMID: 12945615. | Gestational age of participants not reported |
| Pollock KE, Price JR. Phonological skills of children adopted from China: implications for assessment. Semin Speech Lang. 2005 Feb;26(1):54-63. doi: 10.1055/s-2005-864216. PMID: 15731970. | Unable to source |
| Potter NL. Examining speech of very-low-birthweight children during everyday activities. Dev Med Child Neurol. 2010 Jun;52(6):504-5. doi: 10.1111/j.1469-8749.2009.03469.x. Epub 2009 Jan 5. PMID: 20059522. | Wrong publication type |
| Preisser, D. A., Hodson, B. W., & Paden, E. P. Developmental phonology: 18-29 months. The Journal of speech and hearing disorders, 1988; 53(2), 125-130. https://doi.org/doi:https://dx.doi.org/10.1044/jshd.5302.125 | Gestational age of participants not reported |
| Pritchard VE, Bora S, Austin NC, Levin KJ, Woodward LJ. Identifying very preterm children at educational risk using a school readiness framework. Pediatrics. 2014 Sep;134(3):e825-32. doi: 10.1542/peds.2013-3865. Epub 2014 Aug 11. PMID: 25113296. | No relevant outcomes reported (speech sound / intelligibility) |
| Reilly S, Bavin EL, Bretherton L, Conway L, Eadie P, Cini E, … Wake M. (2009). The Early Language in Victoria Study (ELVS): A prospective, longitudinal study of communication skills and expressive vocabulary development at 8, 12 and 24 months. *International Journal of Speech-Language Pathology*, *11*(5), 344–357. https://doi.org/10.1080/17549500903147560 | No preterm participants |
| Reilly S, Cook F, Bavin EL, Bretherton L, Cahir P, Eadie P, Gold L, Mensah F, Papadopoullos S, Wake M. Cohort Profile: The Early Language in Victoria Study (ELVS). Int J Epidemiol. 2018 Feb 1;47(1):11-20. doi: 10.1093/ije/dyx079. PMID: 29040559. | Gestational age of participants not reported |
| Reilly S, Eadie P, Bavin EL, Wake M, Prior M, Williams J, Bretherton L, Barrett Y, Ukoumunne OC. Growth of infant communication between 8 and 12 months: a population study. J Paediatr Child Health. 2006 Dec;42(12):764-70. doi: 10.1111/j.1440-1754.2006.00974.x. PMID: 17096710. | Gestational age of participants not reported |
| Reuterskiöld C, Grigos MI. Nonword Repetition and Speech Motor Control in Children. Biomed Res Int. 2015;2015:683279. doi: 10.1155/2015/683279. Epub 2015 Oct 18. PMID: 26557688; PMCID: PMC4628733. | Gestational age of participants not reported |
| Ribeiro CD, Pachelli MR, Amaral NC, Lamônica DA. Development skills of children born premature with low and very low birth weight. Codas. 2017 Jan 30;29(1):e20160058. Portuguese, English. doi: 10.1590/2317-1782/20162016058. PMID: 28146204. | No relevant outcomes reported (speech sound / intelligibility) |
| Richards JA, Xu D, Gilkerson J, Yapanel U, Gray S, Paul T. Automated Assessment of Child Vocalization Development Using LENA. J Speech Lang Hear Res. 2017 Jul 12;60(7):2047-2063. doi: 10.1044/2017_JSLHR-L-16-0157. PMID: 28609511. | Gestational age of participants not reported |
| Richtsmeier PT. From perceptual learning to speech production: Generalizing phonotactic probabilities in language acquisition. Dissertation Abstracts International Section A: Humanities and Social Sciences. 2009;69(7):2696. https://repository.arizona.edu/handle/10150/194455 | Gestational age of participants not reported |
| Rios NVF, Fernandes LDC, Andrade CLO, Santiago AC, Alves CAD. Phonological productive processes in full-term schoolchildren and small for gestational age: a case-control study. Codas. 2021 Dec 20;34(2):e20200340. doi: 10.1590/2317-1782/20212020340. PMID: 34932658; PMCID: PMC9851191. | No preterm participants |
| Rios NVF, Fernandes LDC, Andrade CLO, Santiago AC, Alves CAD. Phonological productive processes in full-term schoolchildren and small for gestational age: a case-control study. Codas. 2021 Dec 20;34(2):e20200340. doi: 10.1590/2317-1782/20212020340. PMID: 34932658; PMCID: PMC9851191. | Duplicate |
| Robb, M. P., & Bleile, K. M. (1994). Consonant inventories of young children from 8 to 25 months. Clinical Linguistics and Phonetics, 1994; 8(4), 295 EP - 320. | Gestational age of participants not reported |
| Robokos D. Cognitive, language, and social-emotional development among infants and toddlers in early head start: An examination of the impact of cumulative risk. Dissertation Abstracts International: Section B: The Sciences and Engineering. 2007;68(1):652. | No relevant outcomes reported (speech sound / intelligibility) |
| Rochet-Capellan A, Schwartz JL. An articulatory basis for the labial-to-coronal effect: /pata/ seems a more stable articulatory pattern than /tapa/. J Acoust Soc Am. 2007 Jun;121(6):3740-54. doi: 10.1121/1.2734497. PMID: 17552724. | Gestational age of participants not reported |
| Rodrigues JCL, Gaspardo CM, Linhares MBM. Development of neonatal high-risk preterm infants in comparison to full-term counterparts. Appl Neuropsychol Child. 2022 Oct-Dec;11(4):840-849. doi: 10.1080/21622965.2021.1988601. Epub 2021 Oct 18. PMID: 34663137. | No relevant outcomes reported (speech sound / intelligibility) |
| Rothgänger H. Analysis of the sounds of the child in the first year of age and a comparison to the language. Early Hum Dev. 2003 Dec;75(1-2):55-69. doi: 10.1016/j.earlhumdev.2003.09.003. PMID: 14652159. | No preterm participants |
| Roug, L., Landberg, I., & Lundberg, L. J. Phonetic development in early infancy: a study of four Swedish children during the first eighteen months of life. Journal of Child Language, 1989; 16(1), 19 EP - 40. | Gestational age of participants not reported |
| Rvachew S, Alhaidary A, Mattock K, Polka L. Emergence of the corner vowels in the babble produced by infants exposed to Canadian English or Canadian French. Journal of Phonetics. 2008;36(4):564-577. doi: 10.1016/j.wocn.2008.02.001 | Gestational age of participants not reported |
| Rvachew S, Mattock K, Polka L, Ménard L. Developmental and cross-linguistic variation in the infant vowel space: the case of Canadian English and Canadian French. J Acoust Soc Am. 2006 Oct;120(4):2250-9. doi: 10.1121/1.2266460. PMID: 17069320. | No preterm participants |
| Rvachew S. Children with speech sound disorders. The handbook of language and speech disorders (2nd ed.) 2021:313-33. doi: 10.1002/9781119606987.ch15 | Gestational age of participants not reported |
| Sajaniemi N, Hakamies-Blomqvist L, Mäkelä J, Avellan A, Rita H, von Wendt L. Cognitive development, temperament and behavior at 2 years as indicative of language development at 4 years in pre-term infants. Child Psychiatry Hum Dev. 2001 Summer;31(4):329-46. doi: 10.1023/a:1010238523628. PMID: 11227991. | No relevant outcomes reported (speech sound / intelligibility) |
| Saleh M, Shoeib R, Hegazi M, Ali P. Early phonological development in Arabic Egyptian children: 12-30 months. Folia Phoniatr Logop. 2007;59(5):234-40. doi: 10.1159/000104461. PMID: 17726326. | Gestational age of participants not reported |
| Salokorpi, T., Sajaniemi, N., Rajantie, I., Hallback, H., Hamalainen, T., Rita, H., & Von Wendt, L. Neurodevelopment until the adjusted age of 2 years in extremely low birth weight infants after early intervention - A case-control study. Pediatric Rehabilitation, 1998; 2(4), 157 EP - 163. https://doi.org/doi:https://dx.doi.org/10.3109/17518429809060947 | No relevant outcomes reported |
| Sansavini A, Guarini A, Alessandroni R, Faldella G, Giovanelli G, Salvioli G. Are early grammatical and phonological working memory abilities affected by preterm birth? J Commun Disord. 2007 May-Jun;40(3):239-56. doi: 10.1016/j.jcomdis.2006.06.009. Epub 2006 Jul 24. PMID: 16860818. | No relevant outcomes reported (speech sound / intelligibility) |
| Sansavini A, Guarini A, Justice LM, Savini S, Broccoli S, Alessandroni R, Faldella G. Does preterm birth increase a child's risk for language impairment? Early Hum Dev. 2010 Dec;86(12):765-72. doi: 10.1016/j.earlhumdev.2010.08.014. Epub 2010 Sep 16. PMID: 20846796. | No relevant outcomes reported (speech sound / intelligibility) |
| Scattoni ML, Costantino A, Villani A, Mosca F, Zanetto F, Biasci P, Missaglia R. Development of the active surveillance for the neurodevelopmental disorders' early detection: The collaborative approach at the Istituto Superiore di Sanita. Italian Journal of Pediatrics. 2019;45. doi: 10.1186/s13052-019-0746-3 | Wrong publication type |
| Schirmer CR, Portuguez MW, Nunes ML. Clinical assessment of language development in children at age 3 years that were born preterm. Arq Neuropsiquiatr. 2006 Dec;64(4):926-31. doi: 10.1590/s0004-282x2006000600007. PMID: 17220997. | No relevant outcomes reported (speech sound / intelligibility) |
| Scotch AW. As they grow: let's talk. Parents. 2005;80(11):215-216. | Unable to source |
| Seidl A, Cristià A, Bernard A, Onishi KH. (2009). Allophonic and Phonemic Contrasts in Infants’ Learning of Sound Patterns. *Language Learning and Development*, *5*(3), 191–202. https://doi.org/10.1080/15475440902754326 | No preterm participants |
| Seidman, S., Allen, R., & Wasserman, G. A. Productive language of premature and physically handicapped two-year-olds. Journal of communication disorders, 1986; 19(1), 49 EP - 61. https://doi.org/doi: | No relevant outcomes reported |
| Selby JC, Robb MP, Gilbert HR. (2000). Normal vowel articulations between 15 and 36 months of age. *Clinical Linguistics & Phonetics*, *14*(4), 255–265. https://doi.org/10.1080/02699200050023976 | Gestational age of participants not reported |
| Serenius F, Källén K, Blennow M, Ewald U, Fellman V, Holmström G, Lindberg E, Lundqvist P, Maršál K, Norman M, Olhager E, Stigson L, Stjernqvist K, Vollmer B, Strömberg B; EXPRESS Group. Neurodevelopmental outcome in extremely preterm infants at 2.5 years after active perinatal care in Sweden. JAMA. 2013 May 1;309(17):1810-20. doi: 10.1001/jama.2013.3786. PMID: 23632725. | No relevant outcomes reported (speech sound / intelligibility) |
| Serkhane JE, Schwartz JL, BoÃ LJ, Davis BL, Matyear CL. Infants' vocalizations analyzed with an articulatory model: A preliminary report. Journal of Phonetics. 2007;35(3):321-340. doi: 10.1016/j.wocn.2006.10.002 | Gestational age of participants not reported |
| Shahshahani S, Vameghi R, Sajedi F, Biglarian A. Comparing the results of developmental screening of 4 to 60-month-old children in Tehran using parents evaluation of developmental status and ages and stages questionnaires. Iranian Journal of Pediatrics. 2017;27(2):e6179. doi: 10.5812/ijp.6179 | Gestational age of participants not reported |
| Shasidhar RY, Raja Madhusudhan Rao S. Assessing developmental milestones in preterm infants: A prospetive cohort study. International Journal of Medicine and Public Health. 2024;14(2):861. doi: 10.5530/ijmedph.2024.2.166 | No relevant outcomes reported (speech sound / intelligibility) |
| Shriberg, L. D., Kwiatkowski, J., & Gruber, F. A. Developmental phonological disorders. II. Short-term speech-sound normalization. Journal of Speech and Hearing Research, 1994; 37(5), 1127-1150. https://doi.org/doi:https://dx.doi.org/10.1044/jshr.3705.1127 | Gestational age of participants not reported |
| Sikka G, Allman A, Evans J, Sharma A, Goel N, Banerjee S, Kenny C. Two year outcome of infants born >=32 weeks gestation and <=1500g. Archives of Disease in Childhood. 2019;104:A83. doi: 10.1136/archdischild-2019-rcpch.199 | Wrong publication type |
| Silva C, Cadime I, Ribeiro I, Acosta V, Lima R, Viana FL. Communicative development of Portuguese infants aged between 8 and 15 months. Revista de Logopedia, Foniatria y Audiologia. 2017;37(3):121-129. doi: 10.1016/j.rlfa.2016.12.001 | No preterm participants |
| Silva MHD, Wertzner HF, Haynes C, Azoni CAS. Characterization of the phonological system in low-income Brazilian preschoolers. Codas. 2023 Sep 15;35(6):e20220189. doi: 10.1590/2317-1782/20232022189. PMID: 37729319; PMCID: PMC10723580. | Gestational age of participants not reported |
| Smith A, Mason K, Pua E, Nyswonger J, Riski J. Evaluation of speech and velopharyngeal function at 1 year of age as a predictor for outcomes at 5 years of age. Cleft Palate-Craniofacial Journal. 2018;55(1):80. doi: 10.1177/1055665618761376 | Wrong publication type |
| Smith, B. L., Brown-Sweeney, S., & Stoel-Gammon, C. A quantitative analysis of reduplicated and variegated babbling. First Language, 1989; 9(26), 175-189. https://doi.org/doi:https://doi.org/10.1177/014272378900900605 | Gestational age of participants not reported |
| Snedeker J, Geren J, Shafto CL. Disentangling the effects of cognitive development and linguistic expertise: a longitudinal study of the acquisition of English in internationally-adopted children. Cogn Psychol. 2012 Aug;65(1):39-76. doi: 10.1016/j.cogpsych.2012.01.004. Epub 2012 Mar 12. PMID: 22417632. | Gestational age of participants not reported |
| Soli SD, Zheng Y, Meng Z, Li G. Clinical assessment of early language development: a simplified short form of the Mandarin communicative development inventory. Int J Pediatr Otorhinolaryngol. 2012 Sep;76(9):1255-64. doi: 10.1016/j.ijporl.2012.05.015. Epub 2012 Jun 20. PMID: 22727407. | No relevant outcomes reported (speech sound / intelligibility) |
| Somefun OA, Lesi FE, Danfulani MA, Olusanya BO. Communication disorders in Nigerian children. Int J Pediatr Otorhinolaryngol. 2006 Apr;70(4):697-702. doi: 10.1016/j.ijporl.2005.09.003. Epub 2005 Oct 6. PMID: 16213599. | Gestational age of participants not reported |
| Sotto CD, Redle E, Bandaranayake D, Neils-Strunjas J, Creaghead NA. Fricatives at 18 months as a measure for predicting vocabulary and grammar at 24 and 30 months. J Commun Disord. 2014 May-Jun;49:1-12. doi: 10.1016/j.jcomdis.2014.02.003. Epub 2014 Feb 22. PMID: 24674713. | Gestational age of participants not reported |
| Spencer-Smith MM, Spittle AJ, Lee KJ, Doyle LW, Anderson PJ. Bayley-III Cognitive and Language Scales in Preterm Children. Pediatrics. 2015 May;135(5):e1258-65. doi: 10.1542/peds.2014-3039. Epub 2015 Apr 20. PMID: 25896835. | No relevant outcomes reported (speech sound / intelligibility) |
| Sreedevi N, Jyothi S, Nair SK. Phonetic characteristics of vowels in babbling. Journal of the All India Institute of Speech & Hearing. 2012;21:64-67. | Gestational age of participants not reported |
| Srinivasjois R, Silva D, Maina K. Preschool outcomes of late preterm infants-a systematic review. Journal of Paediatrics and Child Health. 2014;50:109. | Wrong publication type |
| Stark, R. E., Bernstein, L. E., & Demorest, M. E. Vocal communication in the first 18 months of life. Journal of Speech and Hearing Research, 1993; 36(3), 548 EP - 558. https://doi.org/doi:https://dx.doi.org/10.1044/jshr.3603.548 | Gestational age of participants not reported |
| Steeve RW, Moore CA, Green JR, Reilly KJ, Ruark McMurtrey J. Babbling, chewing, and sucking: oromandibular coordination at 9 months. J Speech Lang Hear Res. 2008 Dec;51(6):1390-404. doi: 10.1044/1092-4388(2008/07-0046). Epub 2008 Jul 29. PMID: 18664699; PMCID: PMC2890217. | Gestational age of participants not reported |
| Steeve RW. Babbling and Chewing: Jaw Kinematics from 8 to 22 months. J Phon. 2010 Jul 1;38(3):445-458. doi: 10.1016/j.wocn.2010.05.001. PMID: 20725590; PMCID: PMC2921855. | Gestational age of participants not reported |
| Stene-Larsen K, Brandlistuen RE, Lang AM, Landolt MA, Latal B, Vollrath ME. Communication impairments in early term and late preterm children: a prospective cohort study following children to age 36 months. J Pediatr. 2014 Dec;165(6):1123-8. doi: 10.1016/j.jpeds.2014.08.027. Epub 2014 Sep 23. PMID: 25258153. | No relevant outcomes reported (speech sound / intelligibility) |
| Stern E, Cohen N, Odom E, Stroustrup A, Gupta S, Saltzman DH, Rebarber A, Fox NS. Long-term outcomes of twins based on gestational age at delivery. J Matern Fetal Neonatal Med. 2018 Dec;31(23):3102-3107. doi: 10.1080/14767058.2017.1364725. Epub 2017 Aug 17. PMID: 28783997. | No relevant outcomes reported (speech sound / intelligibility) |
| Stoel-Gammon C. Intervocalic consonants in the speech of typically developing children: emergence and early use. Clin Linguist Phon. 2002 Apr-May;16(3):155-68. doi: 10.1080/02699200110112204. PMID: 12064025. | Gestational age of participants not reported |
| Stokes SF, Surendran D. Articulatory complexity, ambient frequency, and functional load as predictors of consonant development in children. J Speech Lang Hear Res. 2005 Jun;48(3):577-91. doi: 10.1044/1092-4388(2005/040). PMID: 16197274. | Gestational age of participants not reported |
| Stokes SF, Wong IM. Vowel and diphthong development in Cantonese-speaking children. Clin Linguist Phon. 2002 Dec;16(8):597-617. doi: 10.1080/0269920021000034967. PMID: 12596428. | Gestational age of participants not reported |
| Sumanasena SP, Vipulaguna DV, Mendis MM, Gunawardena NS. Beyond survival: 5-year neurodevelopmental follow-up of a cohort of preterm infants in Colombo, Sri Lanka. Paediatr Int Child Health. 2018 May;38(2):128-136. doi: 10.1080/20469047.2017.1380944. Epub 2017 Oct 18. PMID: 29043919. | No relevant outcomes reported (speech sound / intelligibility) |
| Swingley D. Phonetic detail in the developing lexicon. Lang Speech. 2003;46(Pt 2-3):265-94. doi: 10.1177/00238309030460021001. PMID: 14748447. | Gestational age of participants not reported |
| Teixeira ER, Davis BL. Early sound patterns in the speech of two Brazilian Portuguese speakers. Lang Speech. 2002 Jun;45(Pt 2):179-204. doi: 10.1177/00238309020450020401. PMID: 12613560. | Gestational age of participants not reported |
| Thiessen ED. (2012). Effects of Inter- and Intra-modal Redundancy on Infants’ Rule Learning. *Language Learning and Development*, *8*(3), 197–214. https://doi.org/10.1080/15475441.2011.583610 | No relevant outcomes reported (speech sound / intelligibility) |
| To CKS, McLeod S, Sam KL, Law T. Predicting Which Children Will Normalize Without Intervention for Speech Sound Disorders. J Speech Lang Hear Res. 2022 May 11;65(5):1724-1741. doi: 10.1044/2022_JSLHR-21-00444. Epub 2022 Apr 5. PMID: 35381182. | Gestational age of participants not reported |
| Tommiska V, Heinonen K, Kero P, Pokela ML, Tammela O, Järvenpää AL, Salokorpi T, Virtanen M, Fellman V. A national two year follow up study of extremely low birthweight infants born in 1996-1997. Arch Dis Child Fetal Neonatal Ed. 2003 Jan;88(1):F29-35. doi: 10.1136/fn.88.1.f29. PMID: 12496223; PMCID: PMC1756010. | No relevant outcomes reported (speech sound / intelligibility) |
| Torrington Eaton C, Newman RS, Ratner NB, Rowe ML. Non-word repetition in 2-year-olds: Replication of an adapted paradigm and a useful methodological extension. Clin Linguist Phon. 2015 Jul;29(7):523-35. doi: 10.3109/02699206.2015.1029594. Epub 2015 Apr 20. PMID: 25894670. | Gestational age of participants not reported |
| Tripathi T, Dusing SC. (2015). Long-term neurodevelopmental outcomes of infants born late preterm: a systematic review. *Research and Reports in Neonatology*, *5*, 91–111. https://doi.org/10.2147/RRN.S44062 | Wrong publication type |
| Tsuji S, Mazuka R, Cristia A, Fikkert P. Even at 4 months, a labial is a good enough coronal, but not vice versa. Cognition. 2015 Jan;134:252-6. doi: 10.1016/j.cognition.2014.10.009. Epub 2014 Nov 20. PMID: 25460397. | No preterm participants |
| Ueda I, Davis S. Promotion and demotion of phonological constraints in the acquisition of the Japanese liquid. Clin Linguist Phon. 2001;15(1-2):29-33. doi: 10.3109/02699200109167626. PMID: 21269094. | Wrong publication type |
| van Batenburg-Eddes T, Henrichs J, Schenk JJ, Sincer I, de Groot L, Hofman A, Jaddoe VW, Verhulst FC, Tiemeier H. Early infant neuromotor assessment is associated with language and nonverbal cognitive function in toddlers: the Generation R Study. J Dev Behav Pediatr. 2013 Jun;34(5):326-34. doi: 10.1097/DBP.0b013e3182961e80. PMID: 23708382. | Gestational age of participants not reported |
| van Kessel-Feddema B, Sondaar M, de Kleine M, Verhaak C, van Baar A. Concordance between school outcomes and developmental follow-up results of very preterm and/or low birth weight children at the age of 5 years. Eur J Pediatr. 2007 Jul;166(7):693-9. doi: 10.1007/s00431-006-0309-7. Epub 2006 Nov 16. PMID: 17109165; PMCID: PMC2190787. | No relevant outcomes reported (speech sound / intelligibility) |
| van Noort-van der Spek IL, Franken MC, Weisglas-Kuperus N. Language functions in preterm-born children: a systematic review and meta-analysis. Pediatrics. 2012 Apr;129(4):745-54. doi: 10.1542/peds.2011-1728. Epub 2012 Mar 19. PMID: 22430458. | Wrong publication type |
| van Noort-van der Spek IL, Franken MC, Weisglas-Kuperus N. Language functions in preterm children: A systematic review and meta-analysis. Acta Paediatrica, International Journal of Paediatrics. 2011;100:16-17. doi: 10.1111/j.1651-2227.2011.02484.x | Wrong publication type |
| Velleman SL. Lexical and phonological development in children with childhood apraxia of speech--a commentary on Stoel-Gammon's 'Relationships between lexical and phonological development in young children'. J Child Lang. 2011 Jan;38(1):82-6. doi: 10.1017/S0305000910000498. Epub 2010 Oct 18. PMID: 20950498. | Wrong publication type |
| Viegas F, Viegas D, Baeck HE. Frequency measurement of vowel formants produced by Brazilian children aged between 4 and 8 years. J Voice. 2015 May;29(3):292-8. doi: 10.1016/j.jvoice.2014.08.001. Epub 2014 Dec 12. PMID: 25510161. | Gestational age of participants not reported |
| Vieira ME, Linhares MB. Developmental outcomes and quality of life in children born preterm at preschool- and school-age. J Pediatr (Rio J). 2011 Jul-Aug;87(4):281-91. English, Portuguese. doi: 10.2223/JPED.2096. Epub 2011 Jul 8. PMID: 21743941. | Wrong publication type |
| Vierhaus M, Lohaus A, Kolling T, Teubert M, Keller H, Fassbender I, … Schwarzer G. (2010). The development of 3- to 9-month-old infants in two cultural contexts: Bayley longitudinal results for Cameroonian and German infants. *European Journal of Developmental Psychology*, *8*(3), 349–366. https://doi.org/10.1080/17405629.2010.505392 | No preterm participants |
| Vogel Sosa A, Stoel-Gammon C. Patterns of intra-word phonological variability during the second year of life. J Child Lang. 2006 Feb;33(1):31-50. doi: 10.1017/s0305000905007166. PMID: 16566319. | Gestational age of participants not reported |
| Vohr, B. R., Garcia-Coll, C., Oh, W., Vohr, B. R., Garcia-Coll, C., & Oh, W. Language and neurodevelopmental outcome of low-birthweight infants at three years. Developmental Medicine & Child Neurology, 1989; 31(5), 582-590. https://doi.org/doi:10.1111/j.1469-8749.1989.tb04043.x | No relevant outcomes reported |
| Vohr, B. R., Garcia Coll, C., & Oh, W. Language development of low-birthweight infants at two years. Developmental medicine and child neurology, 1988; 30(5), 608-615. https://doi.org/doi:https://dx.doi.org/10.1111/j.1469-8749.1988.tb04798.x | No relevant outcomes reported |
| Wake M, Tobin S, Girolametto L, Ukoumunne OC, Gold L, Levickis P, Sheehan J, Goldfeld S, Reilly S. Outcomes of population based language promotion for slow to talk toddlers at ages 2 and 3 years: Let's Learn Language cluster randomised controlled trial. BMJ. 2011 Aug 18;343:d4741. doi: 10.1136/bmj.d4741. PMID: 21852344; PMCID: PMC3191855. | No relevant outcomes reported (speech sound / intelligibility) |
| Walker, J. F., Archibald, L. M., Cherniak, S. R., & Fish, V. G. Articulation rate in 3- and 5-year-old children. Journal of Speech and Hearing Research, 1992; 35(1), 4-13. https://doi.org/doi:https://dx.doi.org/10.1044/jshr.3501.04 | Gestational age of participants not reported |
| Warlaumont AS, Finnegan MK. Learning to Produce Syllabic Speech Sounds via Reward-Modulated Neural Plasticity. PLoS One. 2016 Jan 25;11(1):e0145096. doi: 10.1371/journal.pone.0145096. PMID: 26808148; PMCID: PMC4726623. | Wrong publication type |
| Werwach A, Mürbe D, Schaadt G, Männel C. Infants' vocalizations at 6 months predict their productive vocabulary at one year. Infant Behav Dev. 2021 Aug;64:101588. doi: 10.1016/j.infbeh.2021.101588. Epub 2021 Jun 3. PMID: 34091421. | No preterm participants |
| Wetherby AM, Goldstein H, Cleary J, Allen L, Kublin K. Early Identification of Children With Communication Disorders: Concurrent and Predictive Validity of the CSBS Developmental Profile. Infants & Young Children 16(2):p 161-174, April 2003. | Gestational age of participants not reported |
| Whalen DH, Levitt AG, Goldstein LM. VOT in the babbling of French- and English-learning infants. J Phon. 2007 Jul 1;35(3):341-352. doi: 10.1016/j.wocn.2006.10.001. PMID: 19641636; PMCID: PMC2717044. | Gestational age of participants not reported |
| Whalen, D. H., Levitt, A. G., Hsiao, P. L., & Smorodinsky, I. Intrinsic F0 of vowels in the babbling of 6-, 9-, and 12-month-old French- and English-learning infants. Journal of the Acoustical Society of America, 1995; 97(4), 2533 EP - 2539. https://doi.org/doi:https://dx.doi.org/10.1121/1.411973 | Gestational age of participants not reported |
| White, C. P. A multivariate model of language development in preterm infants from birth to 30 months. Dissertation Abstracts International Section A: Humanities and Social Sciences, 1996; 57(1), 0190-0190. | Unable to source |
| Wiethan FM, Mota HB, Moraes AB. Correlations between vocabulary and phonological acquisition: number of words produced versus acquired consonants. Codas. 2016 Jul-Aug;28(4):379-87. English, Portuguese. doi: 10.1590/2317-1782/20162015108. Epub 2016 Aug 4. PMID: 27509396. | Gestational age of participants not reported |
| Wiethan FM, Nóro LA, Mota HB. Early lexical and phonological acquisition and its relationships. Codas. 2014 Jul-Aug;26(4):260-4. English, Portuguese. doi: 10.1590/2317-1782/201420140030. PMID: 25211683. | Gestational age of participants not reported |
| Winter EL, Caemmerer JM, Trudel SM, deLeyer-Tiarks J, Bray MA, Dale BA, Kaufman AS. Does the Degree of Prematurity Relate to the *Bayley-4* Scores Earned by Matched Samples of Infants and Toddlers across the Cognitive, Language, and Motor Domains? J Intell. 2023 Nov 8;11(11):213. doi: 10.3390/jintelligence11110213. PMID: 37998712; PMCID: PMC10671985. | No relevant outcomes reported (speech sound / intelligibility) |
| Wocadlo C, Rieger I. Phonology, rapid naming and academic achievement in very preterm children at eight years of age. Early Hum Dev. 2007 Jun;83(6):367-77. doi: 10.1016/j.earlhumdev.2006.08.001. Epub 2006 Sep 18. PMID: 16979856. | No relevant outcomes reported (speech sound / intelligibility) |
| Wong HS, Cowan FM, Modi N. The validity of standardised two-year neurodevelopmental status assessed during routine nhs follow-up of children born <30 weeks' gestation. Archives of Disease in Childhood. 2015;100:A47-48. doi: 10.1136/archdischild-2015-308599.109 | Wrong publication type |
| Wong WW, Stokes SF. Cantonese consonantal development: towards a nonlinear account. J Child Lang. 2001 Feb;28(1):195-212. doi: 10.1017/s0305000900004645. PMID: 11258004. | No relevant outcomes reported (speech sound / intelligibility) |
| Wong H, Humphreys A, D’Amore A. 1694 Comparison of two-year neurodevelopmental assessments of infants born <32 weeks gestation by Bayley-III and PARCA-R, pre and during the COVID-19 pandemic. Archives of Disease in Childhood 2021;106:A459-A460. | Wrong publication type |
| Woodward LJ, Moor S, Hood KM, Champion PR, Foster-Cohen S, Inder TE, Austin NC. Very preterm children show impairments across multiple neurodevelopmental domains by age 4 years. Arch Dis Child Fetal Neonatal Ed. 2009 Sep;94(5):F339-44. doi: 10.1136/adc.2008.146282. Epub 2009 Mar 22. PMID: 19307223. | No relevant outcomes reported (speech sound / intelligibility) |
| Yaari M, Mankuta D, Harel-Gadassi A, Friedlander E, Bar-Oz B, Eventov-Friedman S, Maniv N, Zucker D, Yirmiya N. Early developmental trajectories of preterm infants. Res Dev Disabil. 2018 Oct;81:12-23. doi: 10.1016/j.ridd.2017.10.018. Epub 2017 Nov 4. PMID: 29113755. | No relevant outcomes reported (speech sound / intelligibility) |
| Yamashita Y, Nakajima Y, Ueda K, Shimada Y, Hirsh D, Seno T, Smith BA. Acoustic analyses of speech sounds and rhythms in Japanese- and english-learning infants. Front Psychol. 2013 Feb 28;4:57. doi: 10.3389/fpsyg.2013.00057. PMID: 23450824; PMCID: PMC3584442. | Gestational age of participants not reported |
| Yavaş M. Patterns of cluster reduction in the acquisition of #sC onsets: are bilinguals different from monolinguals? Clin Linguist Phon. 2011 Nov;25(11-12):981-8. doi: 10.3109/02699206.2011.616643. PMID: 22106888. | Gestational age of participants not reported |
| Yeh LL, Liu CC. Comparing the Informativeness of Single-Word Samples and Connected Speech Samples in Assessing Speech Sound Disorders. J Speech Lang Hear Res. 2021 Nov 8;64(11):4071-4084. doi: 10.1044/2021_JSLHR-20-00172. Epub 2021 Oct 7. PMID: 34618552. | Gestational age of participants not reported |
| Yoshida KA, Pons F, Maye J, Werker JF. Distributional Phonetic Learning at 10 Months of Age. Infancy. 2010 Jul;15(4):420-433. doi: 10.1111/j.1532-7078.2009.00024.x. Epub 2010 Jan 19. PMID: 32693519. | Gestational age of participants not reported |
| Yung Song J, Sundara M, Demuth K. Phonological constraints on children's production of English third person singular -s. J Speech Lang Hear Res. 2009 Jun;52(3):623-42. doi: 10.1044/1092-4388(2008/07-0258). Epub 2008 Oct 24. PMID: 18952857. | Gestational age of participants not reported |
| Zamuner TS, Gerken L, Hammond M. Phonotactic probabilities in young children's speech production. J Child Lang. 2004 Aug;31(3):515-36. doi: 10.1017/s0305000904006233. PMID: 15612388. | Gestational age of participants not reported |
| Zerbeto AB, Cortelo FM, C Filho ÉB. Association between gestational age and birth weight on the language development of Brazilian children: a systematic review. J Pediatr (Rio J). 2015 Jul-Aug;91(4):326-32. doi: 10.1016/j.jped.2014.11.003. Epub 2015 Apr 23. PMID: 25913048. | No relevant retained papers |
| Zhang M, Gazimbi MM, Chen Z, Zhang B, Chen Y, Yu Y, Tang J. Association between birth weight and neurodevelopment at age 1-6 months: results from the Wuhan Healthy Baby Cohort. BMJ Open. 2020 Jan 2;10(1):e031916. doi: 10.1136/bmjopen-2019-031916. PMID: 31900268; PMCID: PMC6955482. | No relevant outcomes reported (speech sound / intelligibility) |
| Zimmerman E. Do Infants Born Very Premature and Who Have Very Low Birth Weight Catch Up With Their Full Term Peers in Their Language Abilities by Early School Age? J Speech Lang Hear Res. 2018 Jan 22;61(1):53-65. doi: 10.1044/2017_JSLHR-L-16-0150. PMID: 29255846. | Wrong publication type |
| Zuccarini M, Guarini A, Gibertoni D, Suttora C, Aceti A, Corvaglia L, Bello A, Caselli MC, Sansavini A. Describing communication profiles of low-risk preterm and full-term late talkers. J Commun Disord. 2023 Jul-Aug;104:106336. doi: 10.1016/j.jcomdis.2023.106336. Epub 2023 May 29. PMID: 37257297. | Gestational age of participants not reported |
