# Supplementary material for: Characteristics of Speech Sound Development in Preterm‐Born Children: A Scoping Review
Source: Int J Lang Commun Disord. 2026 Oct 16;61(6):e70347. doi: 10.1111/1460-6984.70347 (PMC13674721; doi:10.1111/1460-6984.70347)
Supplement: Supplementary file 2 [file JLCD-61-0-s002.docx]

**Supplementary Material 1. Example search strategy**

Ovid MEDLINE(R) ALL <1946 to April 08, 2026>

1	(preterm* or "pre-term*" or prematur* or "pre-matur*" or (low adj3 ("birth weight*" or birthweight*)) or (age adj3 gestation*) or neonat* or "neo-nat*" or infant* or newborn* or "new* born" or "preemie" or "preemies" or "premies" or "premie" or "VLBW" or "VLBWI" or "VLBW‐I" or "VLBWs" or "LBW" or "LBWI" or "LBWs" or "ELBW" or "ELBWI" or "ELBWs" or "NICU*" or babe or babes or baby or babies).ti,ab,kf.

2	exp Infant/

3	or/1-2

4	((speech or speak* or speech or sound* or articulat* or phonolog* or language or phonetic* or phonomic*) adj5 (impair* or disorder* or dysfunction* or difficult* or retard* or delay* or disabilit* or handicap* or problem* or develop* or produc* or traject* or aware*)).ti,ab,kf.

5	(apraxia or dyspraxia or articulat* or dysarthri* or mute or mutism or "non-speak*" or "non-speech*" or nonspeak* or nonspeech).ti,ab,kf.

6	exp Speech Sound Disorder/

7	exp Speech/

8	exp Speech Disorders/

9	Apraxias/ and exp Speech/

10	or/4-9

11	and/3,10

12	(child* or boy* or girl* or juvenil* or toddler* or infan* or baby or babies).ti,ab,kf.

13	exp child/ or exp infant/

14	or/12-13

15	and/11,14

16	limit 15 to english language
