# Supplementary material for: Characteristics of Speech Sound Development in Preterm‐Born Children: A Scoping Review
Source: Int J Lang Commun Disord. 2026 Oct 16;61(6):e70347. doi: 10.1111/1460-6984.70347 (PMC13674721; doi:10.1111/1460-6984.70347)
Supplement: Supplementary file 3 [file JLCD-61-0-s003.docx]

**Supplementary Material 5. Summary of outcome assessors from included studies**

|  | **Speech and Language Pathologist** | **Psychologist** | **Paediatrician** | **Student** | **Not specified** |
| --- | --- | --- | --- | --- | --- |
| **Brown et al., 1986^68^** | Y | - | - | Y | - |
| **D'Odorico, et al., 2011^50^** | - | - | - | - | Y |
| **Filgueira de Souza e Souza et al., 2019^64^** | - | - | - | - | Y |
| **Goldfield, 2000^65^** | - | - | - | - | Y |
| **Holm and Crosbie, 2010^72^** | Y | - | - | - | - |
| **Hong et al., 2025^51^** | Y | - | - | - | - |
| **Imgrund, Krueger and Getejanc, 2023^52^** | Y | - | - | - | - |
| **Jennische and Sedin, 1998^53^** | Y | - | - | - | - |
| **Jennische and Sedin, 1999a^54^** | Y | - | - | - | - |
| **Jennische and Sedin, 1999b^70^** | Y | - | - | - | - |
| **Jennische and Sedin, 2001^55^** | Y | - | - | - | - |
| **Largo et al., 1986^56^** | - | - | - | - | Y |
| **Largo et al., 1990^69^** | - | - | Y | - | - |
| **Lewis et al., 2002^59^** | Y | - | - | - | - |
| **Luoma et al., 1998^60^** | - | - | - | - | Y |
| **Milankov and Mikov, 2009^61^** | - | - | - | - | Y |
| **Oller et al., 1994^62^** | - | - | - | - | - |
| **Rvachew et al., 2005^47^** | Y | - | - | - | - |
| **Sanchez, et al., 2020^12^** | Y | - | - | - | - |
| **Shahramnia, et al., 2023^66^** | - | - | - | Y | - |
| **Törölä, et al., 2011^48^** | Y | - | - | - | - |
| **Törölä, et al., 2012^49^** | Y | - | - | - | - |
| **van Blankenstein, et al., 2023^57^** | - | - | - | - | Y |
| **van Noort-van der Spek et al., 2010^67^** | Y | - | - | - | - |
| **van Noort-van der Spek et al., 2022a^22^** | Y | - | - | - | - |
| **van Noort-van der Spek et al., 2022b^63^** | Y | - | - | - | - |
| **Wolke and Meyer, 1999^17^** | - | Y | - | - | - |
| **Wolke et al., 2008^58^** | - | Y | Y |  | - |
| **Total** | **9** | **1** | **1** | **1** | **6** |
