# Supplementary material for: Characteristics of Speech Sound Development in Preterm‐Born Children: A Scoping Review
Source: Int J Lang Commun Disord. 2026 Oct 16;61(6):e70347. doi: 10.1111/1460-6984.70347 (PMC13674721; doi:10.1111/1460-6984.70347)
Supplement: Supplementary file 4 [file JLCD-61-0-s004.docx]

**Supplementary Material 3. Summary of outcome measurement tools from included studies**

|  | **Spontaneous speech transcription** | **Validated clinical assessment** | **Parent-reported measure** | **Other** |
| --- | --- | --- | --- | --- |
| **Brown et al., 1986^68^** | **-** | **-** | **-** | **Y** |
| **D'Odorico, et al., 2011^50^** | ✓ | - | - | - |
| **Filgueira de Souza e Souza et al., 2019^64^** |  | ABFW Child Language Test Protocol (validated) | - | - |
| **Goldfield, 2000^65^** | ✓ | - | - | - |
| **Holm and Crosbie, 2010^72^** | - | DEAP Diagnostic Screen | - | - |
| **Hong et al., 2025^51^** | - | U-TAP | - | - |
| **Imgrund, Krueger and Getejanc, 2023^52^** | ✓ | - | - | - |
| **Jennische and Sedin, 1998^53^** | Y | - | - | - |
| **Jennische and Sedin, 1999a^54^** | - | - | Y | - |
| **Jennische and Sedin, 1999b^70^** | - | - | - | Y |
| **Jennische and Sedin, 2001^55^** | - | - | - | View of Assessment of Speech and Language Skills in Children |
| **Largo et al., 1986^56^** | - | - | - | Y |
| **Largo et al., 1990^69^** | - | Swiss German Articulation Assessment | - | - |
| **Lewis et al., 2002^59^** | - | GFTA | - | - |
| **Luoma et al., 1998^60^** | - | NEPSY | - | - |
| **Milankov and Mikov, 2009^61^** | - | Global Articulation Test | - | - |
| **Oller et al., 1994^62^** | Y | - | - | - |
| **Rvachew et al., 2005^47^** | ✓ | - | - | - |
| **Sanchez, et al., 2020^12^** | - | (DEAP) | - | - |
| **Shahramnia, et al., 2023^66^** | - | Persian Test of Speech Sound Production | - | - |
| **Törölä, et al., 2011^48^** | ✓ | - | - | - |
| **Törölä, et al., 2012^49^** | ✓ | - | - | - |
| **van Blankenstein, et al., 2023^57^** | - | Bayley Scales of Infant Development | - | Non-standardised |
| **van Noort-van der Spek et al., 2010^67^** | ✓ | Dutch Assessment of Phonological Development | - | - |
| **van Noort-van der Spek et al., 2022a^22^** | ✓ | Dutch Assessment of Phonological Development | - | - |
| **van Noort-van der Spek et al., 2022b^63^** | ✓ | Dutch Assessment of Phonological Development; Dutch Logo Articulation Assessment | - | - |
| **Wolke and Meyer, 1999^17^** | - | - | - | Y |
| **Wolke et al., 2008^58^** | - | Phonological Abilities Test | - | - |
| **Total** | **11** | **14** | **2** | **5** |

*Note:* ABFW – Child Language Test[REF]; DEAP – Diagnostic Evaluation of Articulation and Phonology; U-TAP - Urimal Test of Articulation and Phonology; GFTA – Goldman-Fristoe Test of Articulation; NEPSY – Developmental Neuropsychological Assessment.
