# Supplementary material for: Characteristics of Speech Sound Development in Preterm‐Born Children: A Scoping Review
Source: Int J Lang Commun Disord. 2026 Oct 16;61(6):e70347. doi: 10.1111/1460-6984.70347 (PMC13674721; doi:10.1111/1460-6984.70347)
Supplement: Supplementary file 6 [file JLCD-61-0-s006.docx]

**Supplementary Material 4. Summary of outcome measures from included studies**

|  | **Vowels** | **Prelinguistic vocalisation** | **Babble (early syllable structure)** | **First words** | **Joining words** | **Phonetic inventory** | **Phonological processes** | **Phonological awareness** | **PCC score** | **Articulation errors** | **Speech motor function** | **Intelligibility** | **Derived outcome** |
| --- | --- | --- | --- | --- | --- | --- | --- | --- | --- | --- | --- | --- | --- |
| **Brown et al., 1986^68^** | - | - | - | - | - | - | - | - | - | - | - | - | ✓ |
| **D'Odorico, et al., 2011^50^** | - | - | ✓ | ✓ | - | ✓ | - | - | - | - | - | - | - |
| **Filgueira de Souza e Souza et al., 2019^64^** | - | - | - | - | - | - | ✓ | - | - | - | - | - | - |
| **Goldfield, 2000^65^** | ✓ | - | - | - | - | - | - | - | - | - | - | - | - |
| **Holm and Crosbie, 2010^72^** | - | - | - | - | - | - | - | - | - | - | - | - | ✓ |
| **Hong et al., 2025^51^** | - | - | - | - | - | - | - | - | ✓ | - | ✓ | - | - |
| **Imgrund, Krueger and Getejanc, 2023^52^** | - | - | - | - | - | - | - | - | ✓ | - | - | - | - |
| **Jennische and Sedin, 1998^53^** | - | - | - | - | - | - | ✓ | - | - | - | ✓ | - | - |
| **Jennische and Sedin, 1999a^54^** | - | - | ✓ | ✓ | ✓ | - | - | - | - | - | - | ✓ | - |
| **Jennische and Sedin, 1999b^70^** | - | - | - | - | - | ✓ | - | - | - | - | ✓ | - | - |
| **Jennische and Sedin, 2001^55^** | - | - | - | - | - | - | ✓ | - | - | - | ✓ | - | - |
| **Largo et al., 1986^56^** |  | ✓ | ✓ | ✓ | ✓ | - | - | - | - | ✓ | - | - | - |
| **Largo et al., 1990^69^** | - | - | - | - | - | - | ✓ | - | - | - | - | - | - |
| **Lewis et al., 2002^59^** | - | - | - | - | - | - | - | - | - | ✓ | - | - | - |
| **Luoma et al., 1998^60^** | - | - | - | - | - | - | - | - | - | ✓ | ✓ | - | - |
| **Milankov and Mikov, 2009^61^** | - | - | - | - | - | - | ✓ | - | - | - | - | - | - |
| **Oller et al., 1994^62^** | ✓ | - | ✓ | - | - | - | - | - | - | - | - | - | - |
| **Rvachew et al., 2005^47^** | - | - | ✓ | ✓ | - | - | - | - | - | - | - | - | - |
| **Sanchez, et al., 2020^12^** | - | - | - | - | - | ✓ | ✓ | - | ✓ | - | ✓ | - | - |
| **Shahramnia, et al., 2023^66^** | - | - | - | - | - | ✓ | ✓ | - | - | - | ✓ | - | - |
| **Törölä, et al., 2011^48^** | - | - | ✓ | - | - | - | - | - | - | - | - | - | - |
| **Törölä, et al., 2012^49^** | - | - | ✓ | ✓ | - | - | - | - | - | - | - | - | - |
| **van Blankenstein, et al., 2023^57^** | - | - | - | ✓ | - | - | - | - | - | - | - | - | - |
| **van Noort-van der Spek et al., 2010^67^** | - | - | - | - | - | ✓ | ✓ | - | - | - | - | - | - |
| **van Noort-van der Spek et al., 2022a^22^** | - | - | - | - | - | ✓ | - | - | - | - | - | - | - |
| **van Noort-van der Spek et al., 2022b^63^** | - | - | - | - | - | ✓ | ✓ | ✓ | ✓ | - | - | - | - |
| **Wolke and Meyer, 1999^17^** | - | - | - | - | - | - | - | - | - | ✓ | - | - | - |
| **Wolke et al., 2008^58^** | - | - | - | - | - | ✓ | ✓ | ✓ | - | - | - | - | - |
| **Total** | **2** | **1** | **7** | **6** | **2** | **8** | **9** | **1** | **4** | **5** | **7** | **1** | **2** |

*Note*: PCC – percentage consonants correct
