# Supplementary material for: Characteristics of Speech Sound Development in Preterm‐Born Children: A Scoping Review
Source: Int J Lang Commun Disord. 2026 Oct 16;61(6):e70347. doi: 10.1111/1460-6984.70347 (PMC13674721; doi:10.1111/1460-6984.70347)
Supplement: Supplementary file 7 [file JLCD-61-0-s007.docx]

**Supplementary Material 6. Characteristics of speech sound development up to age 2 years in preterm children compared to children born at term**

***Note****:* Green highlighting - no difference between PT and FT outcome; orange highlighting – PT obtained poorer outcome compared to FT children.
